# Amplified Recognition of Basic Anions Induced by Cooperative Interaction of Ureido-Binding Sites Preorganized by Azacalix[4]arene Skeleton

**DOI:** 10.1021/acs.joc.5c02079

**Published:** 2026-01-16

**Authors:** Karolína Salvadori, Pavel Matějka, Pavel Lhoták, Olivier Siri

**Affiliations:** † Department of Analytical Chemistry, University of Chemistry and Technology, Prague (UCTP), Technicka 5, Prague 6 166 28, Czech Republic; ‡ Aix-Marseille Université, CNRS UMR 7325 Centre Interdisciplinaire de Nanoscience de Marseille (CINaM), Campus de Luminy, Marseille cedex 09 13288, France; § Institute of Chemical Process Fundamentals of Czech Academy of Sciences v.v.i., Rozvojová 135, Prague 6 16502, Czech Republic; ∥ Department of Physical Chemistry, University of Chemistry and Technology, Prague (UCTP), Technicka 5, Prague 6 166 28, Czech Republic; ⊥ Department of Organic Chemistry, University of Chemistry and Technology, Prague (UCTP), Technicka 5, Prague 6 166 28, Czech Republic

## Abstract

The reaction of tetranitropolyamino carriers with 4-*tert*-butylphenyl isocyanate gives rise to ureido-based receptors. These receptors vary in the acidity of their bridging N*H* groups on supporting skeletons, significantly affecting their complexation ability. While introducing nonbasic anions leads to the independent action of ureido-binding sites, accomplished with low binding efficiency for all studied compounds, the results for basic anions depend on the system used. Here, due to a different electron density distribution on supporting skeletons, the interaction with basic anions may cause unwanted deprotonation (acyclic systems **5**) or lead to anion complexation (macrocycles **6** and **7**). Moreover, in the case of tetraureido azacalix[4]­arene **6**, the addition of carboxylates and phosphate induces system cooperativity, which changes the complex stoichiometry from 1:4 to a 1:2 ratio (receptor: anion) and positively improves binding efficiency.

## Graphical abstract

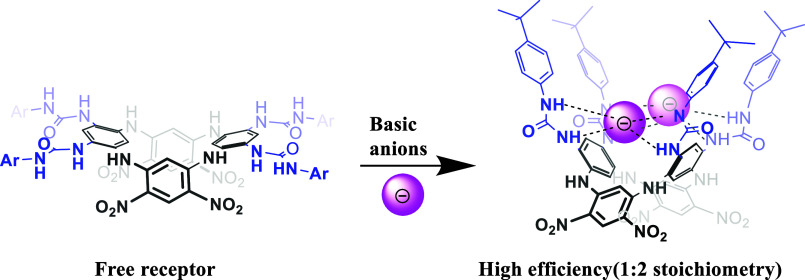

## Introduction

The synthesis of artificial receptors represents the cornerstone of contemporary supramolecular chemistry.^1^ The issue of anion recognition remains one of the hot research topics, especially due to the potential use^2^ of these systems in medicine, environmental chemistry, material recovery, or catalysis. It is therefore not surprising that the research and development of new receptors, together with a detailed analysis of their properties, is gaining importance, as can be seen from a number of articles and reviews dealing with this issue.^3^

Anion complexation employs many different basic strategies and approaches. However, the key point for the construction of any receptor is related to the choice of its binding site. In this regard, both charged and neutral motifs are frequently used. Among the positively charged motifs, ammonium, guanidinium, or amidinium groups occupy a prominent position.^1a^ However, it should be noted that the charged receptors usually suffer from several limitations. They rely on electrostatic interactions, which, in principle, are not very directional. Moreover, these binding sites are usually more sensitive to the pH value of the solution under study. Since it is necessary to maintain charge neutrality, the influence of the original counteranion must be taken into account. For these purposes, charged motifs are often replaced by neutral ones, which can be based on highly directional interactions,^3c^ including hydrogen bonding,^1b,4^ halogen bonding,^5^ or acidic CH bonds,^6^ to name a few.

In the context of this work, the urea-binding site was selected as the preferred motif over other hydrogen-bond (HB) donors. This choice was based on previous research,^7^ confirming the better binding ability of ureido N*H* groups over amidic motifs. Another favorable benefit is the lower tendency of the ureido motif to deprotonate in the presence of basic anions, compared to the sulfonamidic motif^8^ or thioureas.^9^ The history of simple urea-based receptors started in the early 1990s,^10^ and since then, a number of anion receptors comprising this motif have been synthesized. The very first receptors of this type were diphenyl urea derivatives, whose complexation capabilities are well-documented.^11^ Later, it was shown that the efficiency and/or selectivity of ureido interactions can be further enhanced by more sophisticated receptor design, e.g., by using preorganized ureido groups.^12,13^ For this purpose, various molecular platforms providing a suitable arrangement leading to multiple binding events have been tested. This strategy becomes popular, especially in the chemistry of calixarenes,^14,15^ where different conformers have been decorated on their upper or lower rims. Among them, several tetraureido receptors can be found (Figure 1). One of the first derivatives is compound **A**, which is well-known for its self-association into an egg-shaped dimer in nonpolar solvents.^16^ Although this study was developed 30 years ago, the synthesis of new tetraureido derivatives is still vibrant. In this context, Cvetnić et al.^17^ reported in 2024 a detailed description of the binding properties of flexible ureido derivative **B** in acetonitrile. Another interesting tetraureido derivative is compound **C**. Here, the *1,3-alternat*e calix[4]­arene conformation leads to a negative allosteric effect, as a result of which the receptor coordinates exclusively only one anion in a mixture of CDCl_3_ and ACN (4.1; *v*:*v*).^18^

**Figure 1:**
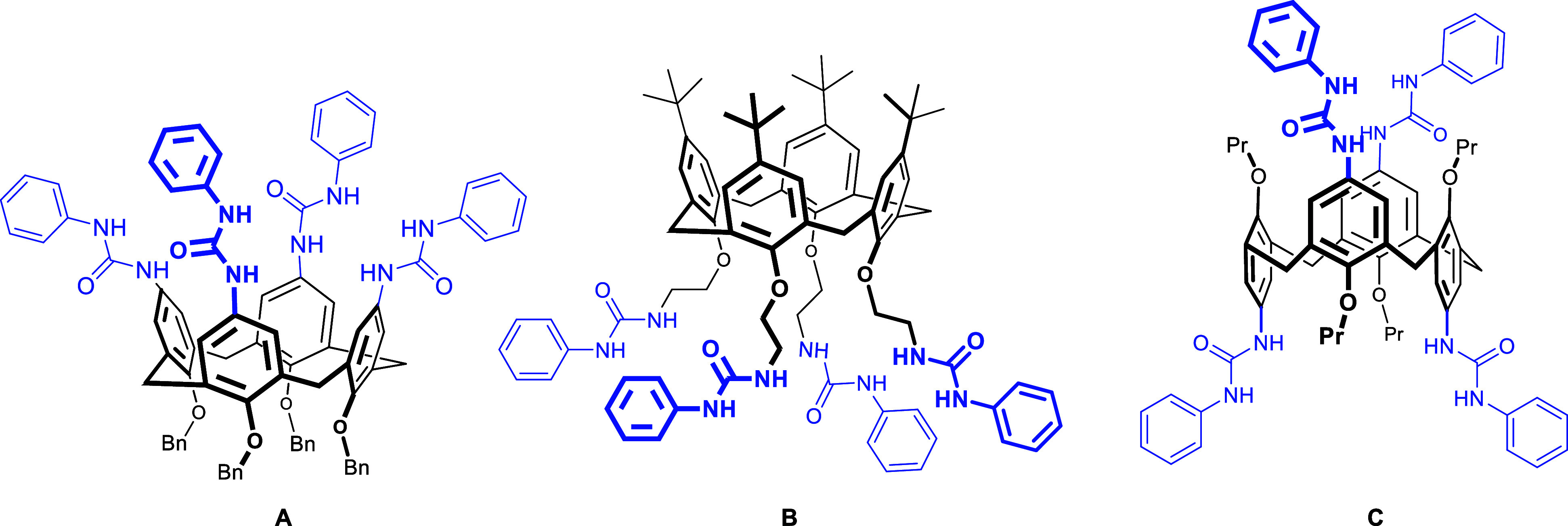
Few examples of tetraureido receptors preorganized by calix[4]­arene.

In addition to the classical calix[4]­arene, this class of compounds is currently represented by many different structures varying in size, bridging atoms, and 3D shapes. In 2008, the calixarene family was extended with a new member—tetranitroazacalix[4]­arene;^19^ its one-pot metal-free synthesis represents a simple and robust way to construct a macrocyclic skeleton (Figure 2a). This macrocycle was found to adopt a stable *1,3-alternate* conformation both in solution and in the solid state and to have a flat cavity.^20^ The study was soon followed by numerous investigations related to several analogues and detailed evaluations of their properties (e.g., redox behavior and/or pH dependence).^21,22^ Regarding the issue of supramolecular receptors, the electron-deficient cavities of cyclophanes for anion recognition have been studied.^23^ However, the use of azacalix[4]­arene for the preorganization of binding sites is still marginal, although several advantages in comparison to classical calixarene might be gained (including distinct positions for skeleton substitution, possible derivatization of bridging nitrogen, or the fact that the scaffold is itself colorful). Only in 2016, tetranitro-azacalix[4]­arene **E** equipped with four amido branches (Figure 2b) was reported as an effective receptor for chlorides in nonpolar solvent.^24^ This study provides valuable information about the complexation properties. However, to the best of our knowledge, no urea receptors have been prepared yet that utilize preorganization via the azacalix[4]­arene skeleton. Therefore, here we wish to report the synthesis and evaluation of the binding properties of the tetraureido azacalix[4]­arene macrocycle **6** and its comparison with model systems equipped with ureido motifs suitable for anion recognition even in an HB-competitive solvent.

**Figure 2:**
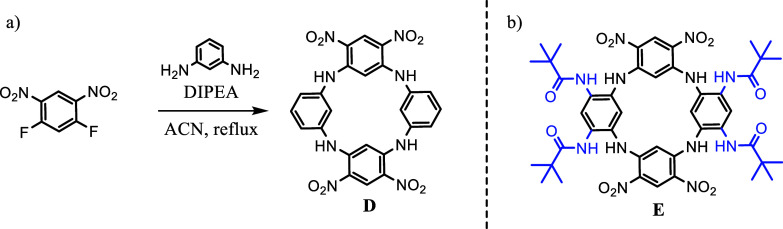
a) Approach to the synthesis of tetranitro-azacalix[4]­arene macrocycle **D** based on nucleophilic aromatic substitution. b) Tetraamido-azacalix[4]­arene receptor; compound suitable for the recognition of chlorides in 1,2-dichloroethane.

## Results and Discussion

The synthesis of tetraureido receptor **6** and its acyclic analogues **5** began with the preparation of suitable scaffolds (Scheme 1). Starting from the nucleophilic aromatic substitution of fluorine in DNFB (1-fluoro-2,4-dinitrobenzene) **1a** or DFDNB (1,5-difluoro-2,4-dinitrobenzene) **1b** with tetraaminobenzene tetrahydrochloride **2** in the presence of DIPEA (*N*,*N*-diisopropylethylamine), a [2 + 1] or [2 + 2] condensation was carried out. After completion of the reaction, the corresponding intermediates **3a**-**b** or the tetraamino-tetranitro azacalix[4]­arene **4a** were isolated as precipitates formed in chilled EtOH in auspicious yields. In the next step, the urea structural motif was introduced by a reaction of amino groups with 4-*tert*-butylphenyl isocyanate (2 molar equiv per one -NH_2_ group). The resulting ureido products were separated from the crude reaction mixtures by preparative TLC on silica gel or trituration, which provided suitable procedures for their purification (yields ∼ 70%).

**Scheme 1:**
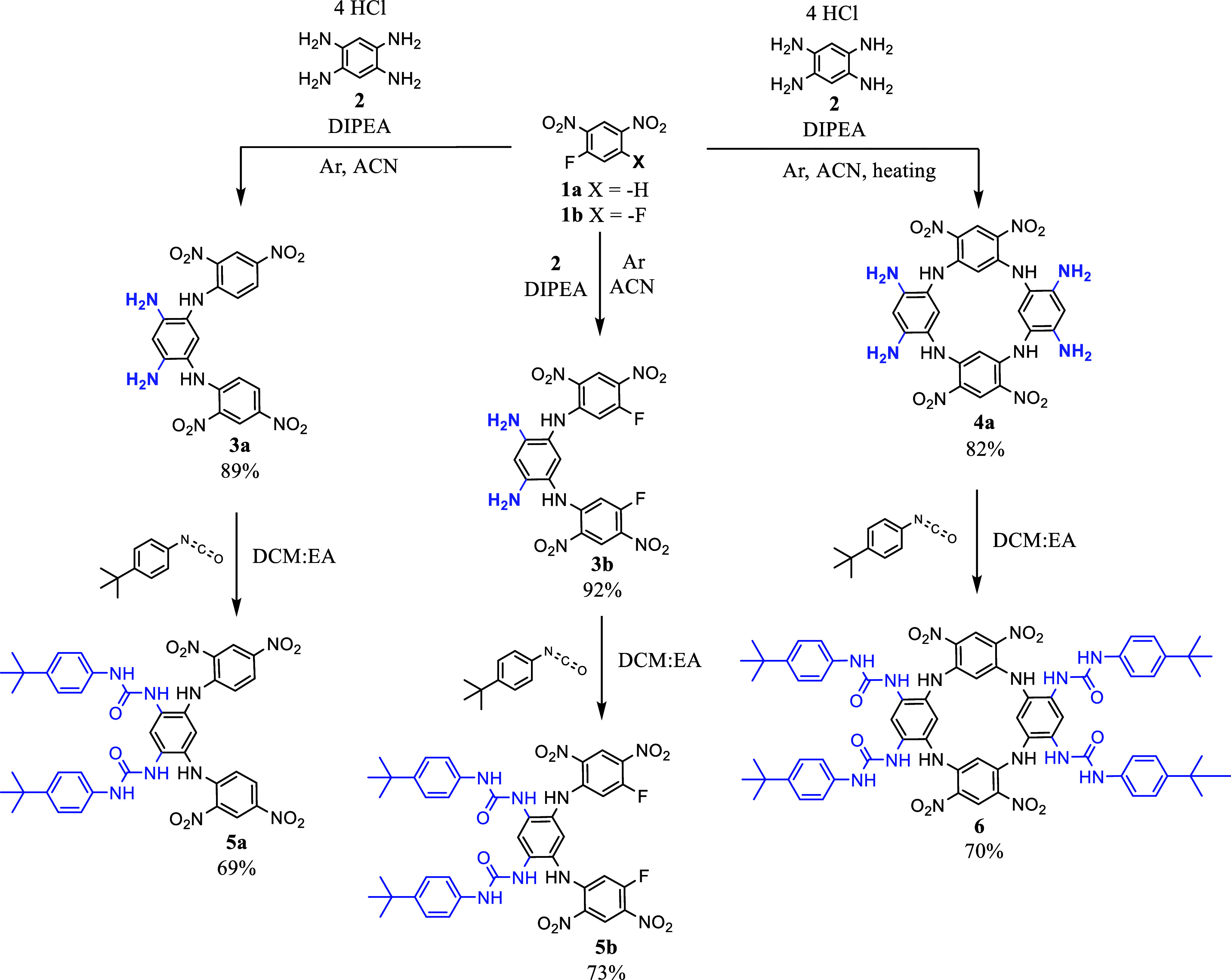
Synthesis of Ureido Azacalix[4]­arene **6** and its Acyclic Analogues **5**

The synthesis and purification steps were followed by confirmation of the structures of receptor **6** and the model systems (**5a**, **5b**). Thus, the HRMS ESI^+^ of **6** showed a peak at *m*/*z* 1327.5404, which is in good agreement with the predicted mass for [M + Na]^+^ (1327.5408; C_68_H_72_N_16_O_12_+Na). The choice of solvent played a crucial role in the NMR analysis. The receptor was practically insoluble in chlorinated solvents (CDCl_3_, CD_2_Cl_2_). In more polar solvents, including acetonitrile-*d*_3_ and acetone-*d*_6_, the compound was soluble; however, its signals were broad and lacked sufficient details of the splitting pattern (Figure S1). Therefore, we decided to use DMSO-*d*_6_ for characterization and measurements. The spectrum of highly symmetrical compound **6** (Figure S2) showed a singlet at 9.53 ppm belonging to the N*H* bridges. In addition, four singlets corresponding to the azacalixarene skeleton were present (9.09, 8.67, 7.02, and 5.42 ppm). Furthermore, singlets at 8.87 and 8.18 ppm (ureido N*H* protons) together with two doublets at 7.29 and 7.20 and a sharp signal at 1.21 ppm reflect the presence of 4-*tert*-butylphenylureido motifs in the structure.

After characterization, the evaluation of supramolecular behavior of the prepared compounds began by monitoring their potential self-aggregation ability. Interestingly, we found out that although DMSO is a highly HB-competitive solvent, the ^1^H NMR spectra are concentration-dependent. The urea’s N*H* showed upfield shifts with decreasing concentration, indicating aggregate decay (Figures S5–S7). The obtained data from dilution experiments were fitted to both cooperative equal (CoEK) and dimer aggregation models in the freely available program Bindfit.^25,26^ Here, a better fit was achieved by the NMR dimer aggregation model, with the obtained equilibrium constants summarized in Table S1. Interestingly, the studied solutions showed remarkable color changes during our investigation. While concentrated solutions showed a reddish hue, increasing dilution resulted in a yellowish color (see Figure S6).

Once we had an insight into the self-aggregation properties, the binding ability toward the anions was monitored. To avoid changes in spectral records caused by dilution, all subsequent experiments were performed at a constant receptor concentration. To understand in more detail the nature of the interaction between receptor **6** (or model **5**) and basic anions (i.e., H_2_PO_4_^–^ or carboxylates), we decided to first monitor their interaction by UV–vis and to compare the result with those obtained in the presence of a strong non-nucleophilic base (1,8-diazabicycloundec-7-ene = DBU). Here, we confirmed that the interaction between receptor **6** and the anion (H_2_PO_4_^–^) was a complexation rather than a simple deprotonation, as only slight modifications of the original receptor band were obtained (see Figure S8). The addition of DBU was associated with a more dramatic change of both the color and absorbance record, as the formation of a new band in the visible region was observed (Figure S10). Surprisingly, a similar response for basic anions and DBU was observed in the case of acyclic derivatives **5**, as a new band above 450 nm was formed (Figures S9–S12). The deprotonation–complexation equilibrium was also evident for acyclic derivatives **5** during the ^1^H NMR titration, where the introduction of H_2_PO_4_^–^ induced intense signal broadening (Figure S13) instead of a simple ureido signal shift observed with Cl^–^ (Figure S14). To determine which groups could deprotonate, we compared the ^1^H NMR spectra of compounds bearing the 4-*tert*-butylphenylureido fragment with respect to the position of the exchangeable N*H* protons (Figure 3). Here, the ureido hydrogens were practically at the same part of the spectra for all studied structures; therefore, we presumed they would not be deprotonated. On the other hand, significant changes observed for N*H* bridges (e.g., 10.13 ppm for **5b** vs 9.53 ppm for **6**) suggest that this might be related to the different electron distribution on the supporting skeletons, leading to higher N*H* acidity for both acyclic systems.

**Figure 3:**
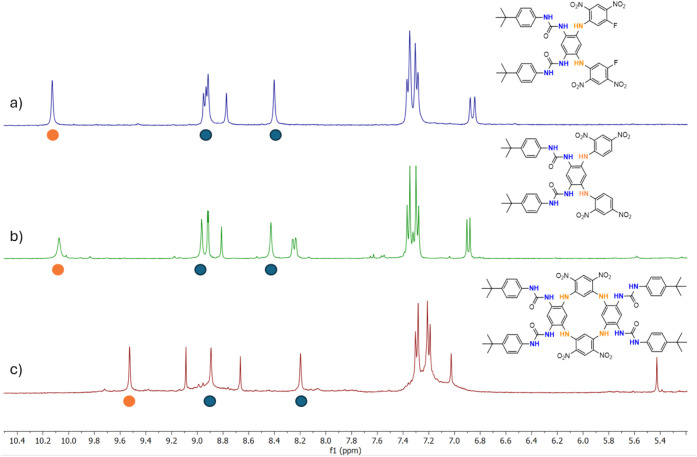
Comparison of the aromatic and N*H* parts in ^1^H NMR of 4-*tert*-butylphenyl ureido compounds (DMSO-*d*_6_, 400 MHz): a) acyclic ureido derivative **5b**; b) acyclic ureido derivative **5a**; c) tetraureido receptor **6**. Blue spot—ureido hydrogens; orange spot—N*H* bridges.

Regarding the previous results, we focused exclusively on macrocyclic receptor **6**. Since the changes in the UV–vis region were moderate upon the addition of basic anions to receptor **6**, we focused on monitoring the interaction between the target guests and the receptor using ^1^H NMR. Starting from a rapid screening of the interactions, a set of anions in the form of tetrabutylammonium (TBA) salts was added to the solution of receptor **6**. Among them, PF_6_^–^, ClO_4_^–^, HSO_4_^–^, Br^–^, and NO_3_^–^ provided only negligible changes, indicating a low attraction toward the ureido binding sites. A somewhat more pronounced change was achieved with Cl^–^. However, the most promising results were observed with basic anions, which are known to be attracted by ureido N*H*-bonds.^13^ A Job plot analysis was performed with selected anions to obtain the stoichiometry of the complexes (Figure S18a,b).^27^ In the case of the basic anion (e.g., benzoate), a maximum was found at 0.33, which corresponds to a stoichiometry of 1:2 (**6**:anion). On the other hand, the nonbasic chloride gave a maximum at 0.20, corresponding to a stoichiometry of 1:4. Moreover, the significant differences obtained for diagnostic signals (C–H between nitro groups) of the azacalixarene skeleton also point to a specific behavior, depending on the anion used. The cooperative binding interaction between the two ureido functions on the opposing aromatic rings with all studied basic anions induces a change in the geometry of the macrocycle (e.g., Figure 4), while no similar effect was observed upon the addition of chloride (the diagnostic signal remains in its initial positions, Figure S16). This suggests that the binding sites operate independently. The efficiency of the interaction was significantly weaker since much more concentrated solutions were required to reach saturation. Thus, the chloride probably does not have the appropriate shape and size for cooperative bonding.

**Figure 4:**
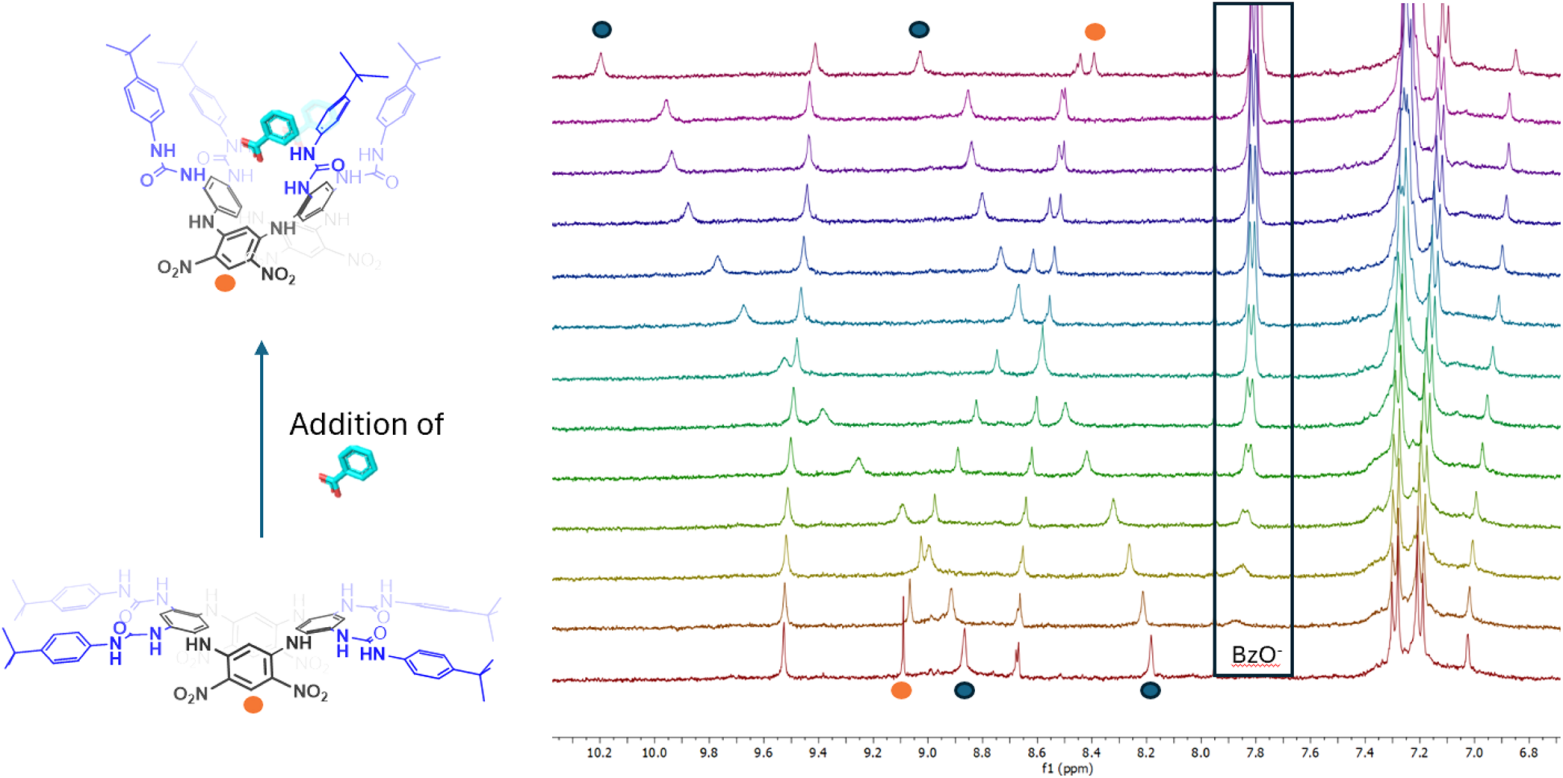
Part of the aromatic and N*H* region during ^1^H NMR titration of receptor **6** (1.20 mM DMSO-*d*_6_, 400 MHz, 298 K) with TBABzO (final concentration of anion 9.90 mM) with highlighted urea N*H* hydrogens (blue) and diagnostic aromatic signals corresponding to the macrocycle (orange; spatially distant from the binding site, making direct electronic effects less likely). For more details regarding concentrations, see Page S17 of the ESI.

Since the stoichiometry of the complexes differs for the individual anions tested, we decided to start evaluating the binding efficiency with a less conventional approach. Instead of using numerical values of the binding constants *K*, which are incomparable for different stoichiometries and highly dependent on the choice of mathematical description, we decided to initially display the raw data graphically. Here, the percentage of the *CIS* (Complexation Induced Shift) value^28^ was advantageously applied; the same receptor concentration was used in the presence of the same amount of added anion (relative to one urea unit). Figure 5a shows four different anions interacting with **6**. At first glance, the results confirmed a much more promising efficiency for basic anions than for Cl^–^. From this point of view, we also indirectly confirm our claim about the cooperativity of the receptor. Under the assumption of independent and identical binding sites with noncooperative behavior, a maximum of 25% chemical shift change (% *CIS*) would be expected at 0.25 equiv of anion per binding site (i.e., 1 equiv of anion per **6**). Notably, the observed % *CIS* values for basic anions significantly exceed this theoretical value, indicating the presence of cooperative effects and deviations from the simple independent action of binding sites. Another interesting phenomenon related to the observed binding preference of receptor **6** suggests that factors beyond basicity contribute to complex stability. It seems that additional noncovalent interactions with the aromatic frameworks are possible (explaining preferences for BzO^–^ over AcO^–^). Moreover, there is a notable difference between BzO^–^ and H_2_PO_4_^–^. The initial part of the record provides more or less the same efficiency for both anions. On the other hand, when adding aliquots exceeding 0.25 equiv per binding site, the receptor begins to significantly prefer phosphates over carboxylates. A possible explanation for this increased stabilization is the dimerization of H_2_PO_4_^–^ inside the supramolecular complex, which has been reported for well-preorganized systems.^29^ To assess the reliability of the conclusions, the evaluation of binding events by classical curve fitting was also used (Figure 5b,c). Here, the values of apparent association constants for stoichiometry 1:2 (receptor: anion) are summarized in Table 1.

**Figure 5:**
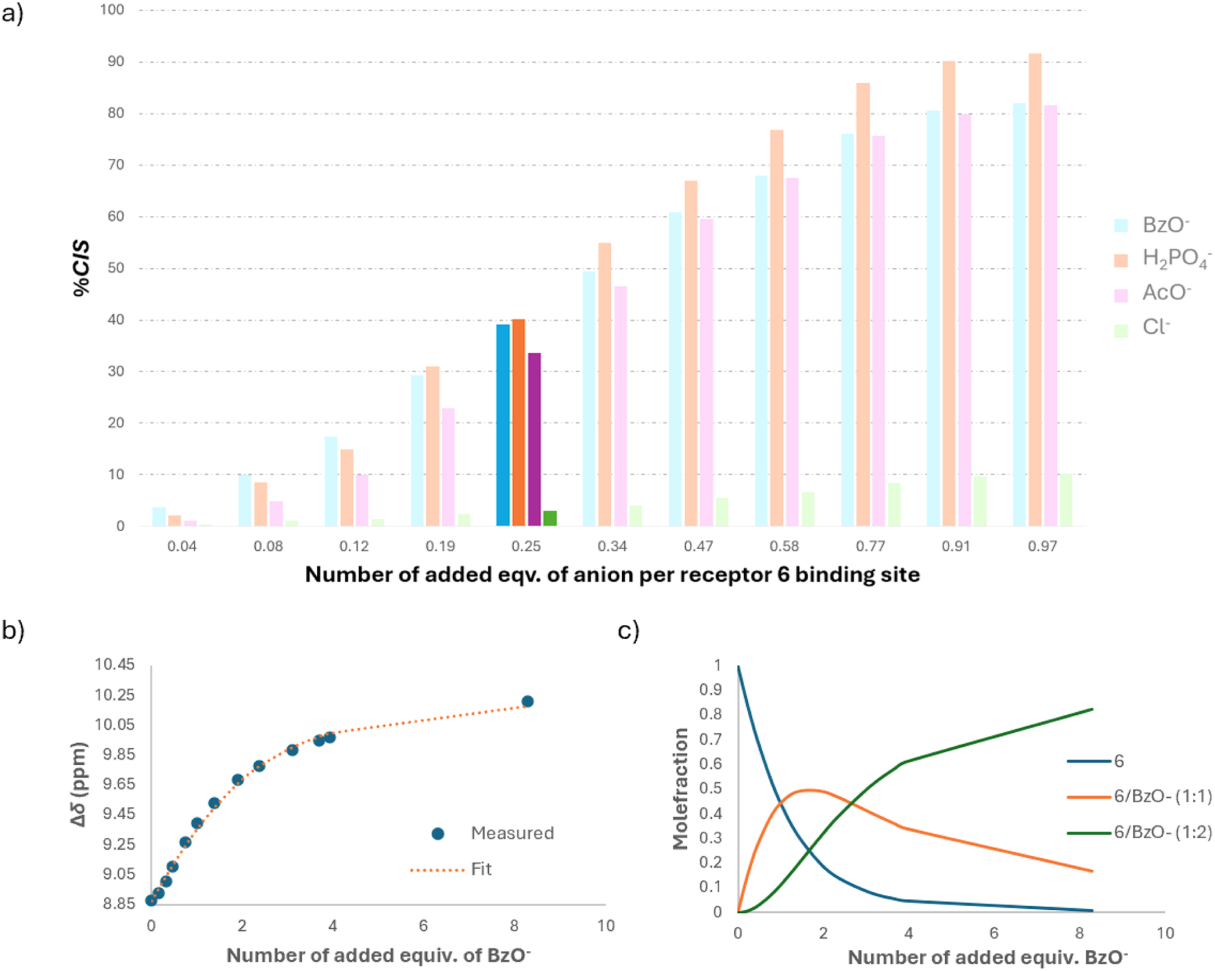
a) Binding efficiency of receptor **6** (1.2 mM, DMSO-*d*_6_) expressed as a percentage of the maximal value of *CIS* depending on the added equivalent of anion per binding unit. The highlighted data correspond to 1.0 molar equiv of the tested anion toward the receptor. b) Δδ of N*H* during the titration of receptor **6** with BzO^–^ displayed together with the fit to experimental data. c) Molar fractions of particular species during the titration.

**Table 1:** Summarizing Information about Binding Events of Receptors **5b** and **6** Together with Apparent Association Constants *K*_As_ (1:1) and Overall Association Constants β, which Were Determined by ^1^H NMR in DMSO-*d*_*6*_ with a Series of Anions in the Form of TBA Salts

**Receptor**	**Anion**	**Description**	***K***_**As**_ ^a^	**β** ^b^
**5b**	H_2_PO_4_^–^	deprotonation	-	-
	Cl^–^	complexation(1:2)	45	5.06 × 10^2^
**6**	H_2_PO_4_^–^	complexation(1:2)	5 060	6.40 × 10^6^
	BzO^–^	complexation(1:2)	2 520	1.59 × 10^6^
	AcO^–^	complexation(1:2)	1 950	9.50 × 10^5^
	Cl^–^	complexation(1:4)	More complex equilibria	

a Error, when estimated, was <20%.

b Evaluated for 1:2 stoichiometry, β = *K*_As_ (1:1) × *K*_As_ (1:2), and *K*_As_ (1:2) = *K*_As_ (1:1)/4.

Finally, to indirectly confirm the positive effect of tetraureido receptor preorganization on the complexation, we attempted to prepare a different kind of model system. This time, compound **7** equipped with ureido functions at the same site of the macrocyclic skeleton, was considered. The synthesis of this derivative is shown in Scheme 2. Briefly, **4b** was prepared by a stepwise approach via acyclic intermediates **3c** (or **3b**), which were subsequently closed with an appropriate aromatic amine. In both cases, macrocycle **4b** was successfully isolated as a brown precipitate in 68% (or 43%) yields, respectively. Then, the crude product was used without any further purification for the introduction of ureido-binding sites by adding 2 molar equiv of 4-*tert*-butylphenyl isocyanate per one -NH_2_ group. Compound **7** was separated on a preparative TLC plate (silica gel), and its structure was confirmed by HRMS and NMR. Here, the ^1^H NMR spectra confirmed the expected *C*_2_ symmetry, as reflected by the presence of 14 signals. Then, dilution experiments in DMSO-*d*_6_ were performed. The obtained equilibrium constants (**5a** > **6** > **7**) indicated a lower self-aggregation ability of compound **7** compared to those of both the tetraureido macrocycle and acyclic derivative.

**Scheme 2:**
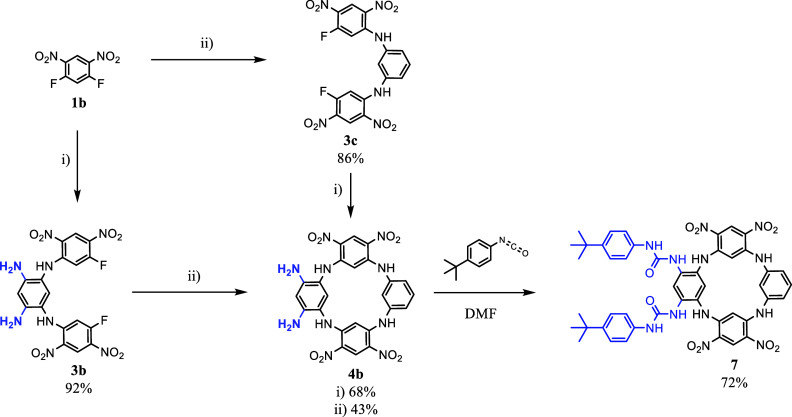
Synthesis of Receptor **7**. (i) Tetraaminobenzene Tetrahydrochloride **2**, DIPEA, ACN, Ar; (ii) 1,3-Diaminobenzene, DIPEA, ACN

The following study of the complexation behavior of compound **7** revealed a 1:2 stoichiometry (**7**:BzO^–^) for the benzoate (Figure S18c), suggesting independent action of both ureido groups. The absence of a cooperative binding partner was clearly evident during the monitoring of the interaction between the receptor and the anion as the apparent constants in DMSO-*d*_6_ correspond to *K*_*A*s_(1:1) = 660 ± 5% and β = 1.09·10^5^. Moreover, it is clear from Figure S17 (^1^H NMR titration of compound **7**) that the introduction of the anion does not induce a geometric change of the macrocyclic platform since the diagnostic proton signal of the macrocycle (the C–H bond between nitro groups, distant from the binding sites) remains at its initial position. We also decided to perform a direct comparison of receptors **6** and **7**. For this purpose, a 2.4 mM solution of compound **7** (exact concentration of binding sites as for **6**) with the same added aliquots of TBABzO was used. In Figure 6, a significant decrease in complexation efficiency is observed, confirming the undisputable role of binding site cooperation for receptor efficiency.

**Figure 6:**
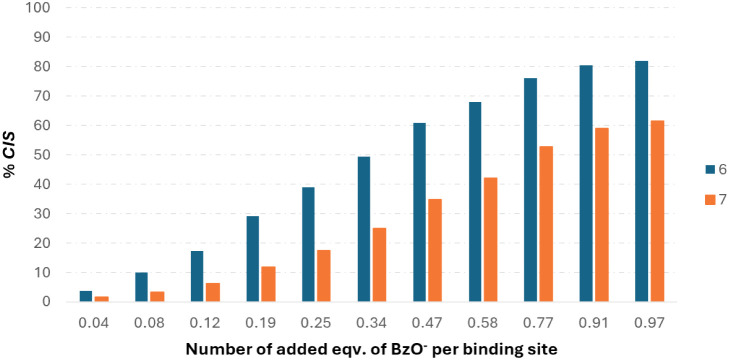
Comparison of the binding efficiency of receptors **6** (1.2 mM, DMSO-*d*_6_) and **7** (2.4 mM, DMSO-*d*_6_) for recognition of TBABzO expressed as a percentage of the maximal value of *CIS*, depending on the added equivalent of anion per ureido binding unit.

## Conclusions

In conclusion, a series of ureido derivatives, including macrocyclic receptors and acyclic model systems, was prepared. We confirmed the formation of self-aggregates even in the presence of highly competitive solvents (e.g., DMSO). Although the compounds aggregate, they still interact with anions. The ^1^H NMR and UV–vis titrations demonstrated that the interaction with basic anions can cause unwanted deprotonation (acyclic systems **5**) or lead to their complexation (macrocycles **6** and **7**). In the case of tetraureido derivative **6**, the complexation ability toward basic anions was further enhanced by a cooperative binding effect (stoichiometry 1:2 (**6**:anion)). On the other hand, upon the addition of nonbasic anions (e.g., Cl^–^), an independent action of ureido motifs (1:4 stoichiometry (**6**:anion)) with low complexation ability was observed. The study revealed the indispensable role of molecular design principles and supramolecular chemistry methods for obtaining receptors with superior efficiency. Moreover, it was demonstrated that decorated tetranitro-azacalix[4]­arene skeletons can be used as a suitable platform for the preorganization of the corresponding binding sites.

## Experimental Section

### General Information

All chemicals were purchased from commercial sources and were used without further purification, except for dried solvents. Anhydrous solvents were dried and stored according to standard procedures. Analytical TLC was carried out on foil sheets coated with silica gel containing a fluorescent indicator 60 F254 (Merck), used for monitoring the reaction progress during synthesis. The analyte was detected by UV light (wavelength, 254 nm). Urea derivatives were isolated from the crude reaction mixtures containing impurities by using trituration or preparative TLC chromatography, which was carried out on self-made glass plates (20 × 20 cm) covered by silica gel 60 PF254 for preparative layer chromatography (Merck).

All prepared compounds were fully characterized by using multinuclear NMR (^1^H NMR, ^13^C NMR, and ^19^F NMR), IR spectroscopy, and mass spectrometry. This information is summarized in the ESI.

The ^1^H (400.1 MHz), ^13^C (100.6 MHz), and ^19^F (376.5 MHz) spectra were recorded using a Bruker Avance 400 spectrometer or JEOL ECS 400 Hz spectrometer at 25 °C. Deuterated solvents used, some of them containing an internal standard (TMS: tetramethylsilane), are indicated in each case. The ^1^H and ^13^C NMR spectra were referenced to the line of the used solvent (δ/ppm; δ_H_/δ_C_: DMSO-*d*_6_, 2.50/39.52 ppm), which was stored over molecular sieves. The ^19^F spectra were referenced to the line of standard hexafluorobenzene (δ_F_/ppm; −163.00). Chemical shifts (δ) are reported in parts per million (ppm), and coupling constants (*J*) are given in Hertz (Hz). Data are reported as follows: chemical shift, multiplicity (s: singlet, d: doublet, t: triplet, dd: doublet of doublet, m: multiplet, brs: broad signal), integration, and coupling constant. Spectra were processed using MestReNova 15.0.1.

The FTIR analysis was performed on a Nicolet iS50 spectrometer (Thermo-Nicolet, USA) connected with a GladiATR diamond placed outside the conventional sample compartment, equipped with a DTGS KBr detector. Reflectance data were acquired with the following parameters: spectral range: 4000–400 cm^–1^; resolution: 4 cm^–1^; number of spectra accumulations: 64; apodization: Happ-Genzel. The spectra were collected and processed by Omnic 9 (Thermo-Nicolet Instruments Co., USA), including baseline correction and a Savitzky–Golay smoothing filter (set to the number of 11 points used in the algorithm).

The HRMS spectra were recorded on a MicrOtof III (Bruker Daltonik, Bremen, Germany) with ESI or APCI ionization sources (in positive mode). For calibration of accurate masses, an ESI-APCI Low Concentration Tuning Mix (Agilent) was used. The samples were delivered into the ion source in methanol or acetonitrile solution.

Melting points were measured on a Heiztisch Mikroskop-Polytherm A (Wagner and Munz, Germany) and are not corrected.

### Titration Measurements

All prepared urea-based receptors were studied by dilution experiments, which were performed in DMSO-*d*_6_ at concentrations covering the range from 25.5 to 0.5 mM. The changes in spectra were monitored by ^1^H NMR. In most cases, urea N*H* signals of receptors revealed significant upfield shifts with decreasing compound concentration. The data from appropriate measurements (see Figure S5–S7) were fitted to both cooperative equal and dimer-aggregation models using the freely available program Bindfit.^25^ The choice between those models was based on the goodness of the fit as reported by Thordarson.^26^

^*1*^ *H NMR titrations for evaluation of binding efficiency by % CIS:* The measurements were performed in DMSO-*d*_6_ at a constant concentration of receptor (1.2 mM for **6**; 2.4 mM for **7**) to which predefined aliquots of anions in the form of their TBA salt were gradually added. According to the preliminary studies, the amount of anion to reach the final *CIS* value was evaluated. Therefore, the following titration experiments covered not only the studied region (displayed in our dependencies in the article above) but also included the whole complexation range to reach binding saturation. The knowledge of the “edge” shifts of ureido hydrogens (initial and ending points of titration equilibria) allowed us to evaluate a % *CIS* at each point, as indicated in several examples (see Figure S20–S24).

^*1*^ *H NMR titrations for evaluation of apparent association constants:* The numerical values of association constants and their errors were evaluated using the freeware program Bindfit.^25^ All of the data relating to the calculations of those constants can be accessed online through the links given for each association event in ESI.

*UV–vis titrations:* The UV–vis titrations were performed in DMSO (HPLC grade 99.9%, Merck), using a Shimadzu double beam UV-1800 spectrophotometer. Titrations were standardly performed at a constant concentration of receptor (specific for individual measurements). All spectra were recorded in the wavelength region from 270 to 800 nm, with steps of 1 nm, using cuvettes with path lengths of 1 mm.

### Synthetic Procedures

#### Synthetic Procedures for Supporting Skeletons

##### Acyclic Precursor **3a**

The amount of 0.106 g (0.37 mmol) of compound **2** (tetraaminobenzene tetrahydrochloride) was dissolved in 50 mL of dry acetonitrile (ACN). To the solution, 100 μL (0.80 mmol) of 1-fluoro-2,4-dinitrobenzene **1a** was added. Before the addition of the base, oxygen was removed from the solution by a stream of argon. Then, 0.5 mL (2.87 mmol) of DIPEA was added dropwise under stirring. The reaction was left overnight at ambient temperature. The next day, the solvent was evaporated, and the solid residue was repeatedly treated with cooled EtOH (100 mL). The product was obtained as a brown powder (0.156 g, 89%). mp 215–216 °C. ^1^H NMR (400 MHz, DMSO-*d*_6_) δ: 9.56 (s, 2H, N*H*); 8.88 (d, 2H, Ar*H*, *J* = 2.7 Hz); 8.22 (dd, 2H, Ar*H*, *J*_1_ = 9.6 Hz, *J*_2_ = 2.7 Hz); 6.82 (d, 2H, Ar*H*, *J* = 9.5 Hz); 6.82 (s, 1H, Ar*H*); 6.21 (s, 1H, Ar*H*); 5.23 (s, 4H, NH_2_) ppm. ^13^C­{^1^H} (100 MHz, DMSO-*d*_6_) δ: 148.9; 145.5; 135.5; 130.8; 129.5; 128.2; 123.2; 117.0; 110.7; 100.0 ppm. IR (ATR) 3473; 3436; 3375; 3332; 3207; 3106; 2984; 2936; 1615; 1581; 1514 cm^–1^. HRMS (ESI) *m*/*z*: [M]^+^ Calcd for C_18_H_14_N_8_O_8_ 470.0929; Found 470.0939.

##### Acyclic Precursor **3b**

The amount of 0.202 g (0.70 mmol) of compound **2** (tetraaminobenzene tetrahydrochloride) was dissolved in 50 mL of dry ACN. To the stirred solution, 0.287 g (1.41 mmol) of 1,5-difluoro-2,4-dinitrobenzene **1b** was added. Before the addition of the base, oxygen was removed from the solution by a stream of argon. Then, 1 mL (5.74 mmol) of DIPEA was added dropwise under stirring. The reaction mixture was left overnight at room temperature. The next day, the solvent was evaporated, and the solid residue was repeatedly treated with cooled EtOH (100 mL). The product was obtained as a russet powder (0.328 g, 92%). mp 201–204 °C. ^1^H NMR (400 MHz, DMSO-*d*_6_) δ: 9.65 (s, 2H, N*H*); 8.90 (d, 2H, Ar*H*, *J* = 7.9 Hz); 6.80 (s, 1H, Ar*H*); 6.53 (d, 2H, Ar*H*, *J* = 14.5 Hz); 6.20 (s, 1H, Ar*H*); 5.28 (s, 4H, NH_2_) ppm. ^19^F NMR (376 MHz, DMSO-*d*_6_) δ: −109.05 ppm. ^13^C­{^1^H} NMR (100 MHz, DMSO-*d*_6_) δ: 158.7 (d, *J* = 263.9 Hz); 150.0 (d, *J* = 13.2 Hz); 145.4; 128.05; 128.03; 127.0; 125.2 (d, *J* = 9.8 Hz); 110.5; 103.1 (d, *J* = 27.2 Hz); 100.0 ppm. IR (ATR) 3436; 3359; 3264; 3099; 1626; 1579; 1514 cm^–1^. HRMS (APCI) *m*/*z*: [M]^+^ Calcd for C_18_H_12_F_2_N_8_O_8_ 506.0741; Found 506.0742, *m*/*z*: [M + Na]^+^ Calcd for C_18_H_12_F_2_N_8_O_8_Na 529.0638; Found 529.0641.

##### Acyclic Precursor **3c**

The amount of 0.101 g (0.92 mmol) of 1,3-diaminobenzene was dissolved in 50 mL of dry ACN. To the stirred solution, 0.420 g (2.06 mmol) of 1,5-difluoro-2,4-dinitrobenzene was added. Before the addition of the base, oxygen was removed from the solution by a stream of argon. Then, 1.3 mL (7.46 mmol) of DIPEA was added dropwise under stirring. The reaction was left overnight at room temperature. The next day, the solvent was evaporated, and the solid residue was repeatedly treated with cooled EtOH (100 mL). The product was obtained as a yellowish powder (0.379 g, 86%). mp 218–210 °C. ^1^H NMR (400 MHz, DMSO-*d*_6_) δ: 10.23 (s, 2H, N*H*); 8.92 (d, 2H, Ar*H*, *J* = 7.9 Hz); 7.62 (t, 1H, Ar*H*, *J* = 8.0 Hz); 7.42 (t, 1H, Ar*H*, *J* = 1.9 Hz); 7.38 (dd, 2H, Ar*H*, *J*_1_ = 7.9 Hz, *J*_2_ = 2.0 Hz); 7.10 (d, 2H, ArH, *J* = 14.2 Hz) ppm. ^19^F NMR (376 MHz, DMSO-*d*_6_) δ: −107.47 ppm. ^13^C­{^1^H} NMR (100 MHz, DMSO-*d*_6_) δ: 158.7 (d, *J* = 265.2 Hz); 147.4 (d, *J* = 13.5 Hz); 138.8; 131.1; 128.6; 127.2; 126.3 (d, *J* = 9.6 Hz); 123.9; 122.3; 103.9 (d, *J* = 27.4 Hz) ppm. IR (ATR) 3347; 3311; 3293; 3099; 3083; 3064; 1625; 1572; 1502 cm^–1^. HRMS (ESI) *m*/*z*: [M+NH_4_]^+^ Calcd for C_18_H_14_F_2_N_7_O_8_ 494.0866; Found 494.0854, *m*/*z*: [M + Na]^+^ Calcd for C_18_H_10_F_2_N_6_O_8_Na 499.0420; Found 499.0406 [M + Na]^+^.

##### Tetraamino-tetranitroazacalix­[4]­arene **4a**

The procedure was described in the literature.^22^ The product was obtained as a brown powder in 82% yield (0.435 g). mp >300 °C. ^1^H NMR (400 MHz, DMSO-*d*_6_) δ: 8.99 (s, 2H, Ar*H*); 8.88 (s, 4H, N*H*); 6.52 (s, 2H, Ar*H*); 6.06 (s, 2H, Ar*H*); 5.69 (s, 2H, Ar*H*); 5.00 (brs, 8H, NH_2_) ppm. ^13^C­{^1^H} (100 MHz, DMSO-*d*_6_) δ: 149.5; 145.4; 128.6; 128.0; 124.7; 110.7; 100.2; 93.6 ppm. IR (ATR): 3451; 3338; 3196; 3095; 2970; 1606; 1562; 1513 cm^–1^. HRMS (ESI) *m*/*z*: [M + H]^+^ Calcd for C_24_H_21_N_12_O_8_ 605.1600; Found 605.1593.

##### Diamino-tetranitroazacalix­[4]­arene **4b**

Procedure (i): The synthesis starts with the dissolution of acyclic precursor **3c** 0.0998 g (0.21 mmol) together with 0.0644 g (0.23 mmol) of compound **2** (tetraaminobenzene tetrahydrochloride) in 40 mL of dry ACN. Then, oxygen was removed from the solution by a stream of argon. To this solution, 0.2 mL (1.15 mmol) of DIPEA was added dropwise under stirring. The reaction mixture was left at ambient temperature. The next day, the solvent was evaporated, and the solid residue was repeatedly treated with cooled EtOH (100 mL).

Procedure (ii): The synthesis starts with the dissolution of acyclic precursor **3b** 0.0870 g (0.17 mmol) together with 0.0204 g (0.19 mmol) of 1,3-diaminobenzene in 40 mL of dry ACN. Then, the oxygen was removed from the solution by a stream of argon. To this solution, 0.2 mL (1.15 mmol) of DIPEA was added dropwise under stirring. The reaction was left at ambient temperature. The next day, the solvent was evaporated, and the solid residue was repeatedly treated with cooled EtOH (100 mL).

The product was obtained as a brown powder in 68% (0.082 g; procedure (i)) or 43% (0.042 g; procedure (ii)) yields. mp 293–297 °C. ^1^H NMR (400 MHz, DMSO-*d*_6_) δ: 9.59 (s, 2H, N*H*); 9.01 + 8.99 (s+s, 2H, Ar*H* + 2H, N*H*); 7.51 (t, 1H, Ar*H*, *J* = 8.2 Hz); 7.18–7.12 (d+s, 3H, Ar*H*); 6.49 (s, 1H, Ar*H*); 5.99 (s, 1H, Ar*H*); 5.52 (s, 2H, Ar*H*); 5.03 (brs, 4H, NH_2_) ppm. ^13^C­{^1^H} (100 MHz, DMSO-*d*_6_) δ: 149.7; 147.6; 145.6; 139.2; 131.6; 128.8; 128.0; 126.80; 126.76; 125.1; 124.1; 110.6; 99.7; 95.0 ppm. IR (ATR) 3466; 3382; 3336; 3199; 3099; 2976; 1622; 1567; 1518 cm^–1^. HRMS (ESI) *m*/*z*: [M + H]^+^ Calcd for C_24_H_19_N_10_O_8_ 575.1382; Found 575.1377.

### General Procedure for Synthesis of Di- and Tetra-Ureido Derivatives

Corresponding supporting skeleton (**3** or **4**) was dissolved in 2 mL of a mixture of dichloromethane (DCM) and ethyl acetate (EA) (3:1 (*v*:*v*)) or in 0.5 mL of dry dimethylformamide (DMF). Then, to the solution, an appropriate amount of *tert*-butylphenyl isocyanate (2 molar equiv per free NH_2_ group) was added dropwise. The reaction was stirred at ambient temperature for 3 days. Then, 5 mL of MeOH was added to the solution. The crude mixture was evaporated. The obtained solid residue was separated by preparative thin-layer chromatography on silica gel. The products were usually stacked on a baseline, and if necessary, the TLC plate was eluted several times.

#### Acyclic Bis-Ureido Derivatives **5a**

Compound **5a** was prepared according to the general procedure above by the reaction of acyclic precursor **3a** (0.052 mg; 0.11 mmol), which was dissolved in 2 mL of a mixture (DCM:EA). The precursor was reacted with 75 μL of *tert*-butylphenyl isocyanate (0.41 mmol). The product was purified by using repetitive preparative TLC (eluent DCM). The product was obtained as a red powder (0.0626 g, 69%). ^1^H NMR (400 MHz, DMSO-*d*_6_) δ: 10.08 (s, 2H, N*H*); 8.98 (s, 2H, N*H*); 8.92 (d, 2H, Ar*H*, *J* = 2.7 Hz); 8.82 (s, 1H, Ar*H*); 8.44 (s, 2H, N*H*); 8.25 (dd, 2H, ArH, *J*_1_ = 9.6 Hz; *J*_2_ = 2.7 Hz); 7.38–7.33 (m, 5H, Ar*H*); 7.29 (d, 4H, Ar*H*, *J* = 8.8 Hz); 6.90 (d, 2H, Ar*H*, *J* = 9.6 Hz); 1.25 (s, 18H, −CH_3_) ppm. ^13^C­{^1^H} NMR (100 MHz, DMSO-*d*_6_) δ: 152.5; 147.8; 144.5; 136.6; 136.4; 136.0; 131.3; 130.0; 127.8; 125.5; 123.2; 122.8; 118.2; 117.1; 113.9; 33.9; 31.2 ppm. IR (ATR) 3310; 3196; 3106; 2959; 2906; 2857; 1697; 1616; 1588; 1511 cm^–1^. HRMS (ESI) *m*/*z*: [M + H]^+^ Calcd for C_40_H_41_N_10_O_10_ 821.3002; Found 821.2994, *m*/*z*: [2M + H]^+^ Calcd for C_80_H_81_N_20_O_20_ 1641.5936; Found 1641.5839.

#### Acyclic Bis-Ureido Derivatives **5b**

Compound **5b** was prepared according to the general procedure above by the reaction of acyclic precursor **3b** (0.050 mg, 0.09 mmol), which was dissolved in 2 mL of a mixture (DCM:EA). The precursor was reacted with 70 μL of *tert*-butylphenyl isocyanate (0.38 mmol). The product was purified by trituration with a small amount of acetone. The product was obtained as an orange powder (0.0544 g, 73%). mp 261–265 °C. ^1^H NMR (400 MHz, DMSO-*d*_6_) δ: 10.13 (s, 2H, N*H*); 8.95 (d, 2H, Ar*H*, *J* = 7.9 Hz); 8.91 (s, 2H, N*H*); 8.79 (s, 1H, Ar*H*); 8.40 (s, 2H, N*H*); 7.39–7.33 (m, 5H, Ar*H*); 7.30 (d, 4H, Ar*H*, *J* = 8.8 Hz); 6.87 (d, 2H, Ar*H*, *J* = 14.2 Hz); 1.26 (s, 18H, −CH_3_) ppm. ^19^F NMR (376 MHz, DMSO-*d*_6_) δ: −107.56 ppm. ^13^C­{^1^H} NMR (100 MHz, DMSO-*d*_6_) δ: 158.8 (*J* = 266.0 Hz); 152.5; 149.0 (*J* = 13.3 Hz); 144.6; 136.6; 136.1; 128.2; 127.3; 127.1; 126.2 (*J* = 9.5 Hz); 125.5; 122.6; 118.2; 114.2; 103.9 (*J* = 27.4 Hz); 33.9; 31.2 ppm. IR (ATR): 3296; 3198; 3104; 3064; 2960; 2906; 2868; 1704; 1630; 1580; 1511 cm^–1^. HRMS (ESI) *m*/*z*: [M + Na]^+^ Calcd for C_40_H_38_F_2_N_10_O_10_Na 879.2632; Found 879.2629, *m*/*z*: [2M + Na]^+^ Calcd for C_80_H_76_F_4_N_20_O_20_Na 1735.537; Found 1735.5357.

#### Tetraureido Azacalix[4]­arene **6**

Tetraureido derivative **6** was prepared according to the general procedure above by the reaction of macrocyclic precursor **4a** 0.048 g (0.08 mmol), which was dissolved in 2 mL of a mixture (DCM:EA). The precursor was reacted with 120 μL of *tert*-butylphenyl isocyanate (0.65 mmol). The product was purified by using repetitive preparative TLC (eluent DCM). The product was obtained as an orange-brown powder (0.0726 mg, 70%). ^1^H NMR (400 MHz, DMSO-*d*_6_) δ: 9.53 (s, 4H, N*H*); 9.09 (s, 2H, Ar*H*); 8.93 (s, 4H, N*H*); 8.66 (s, 2H, Ar*H*); 8.22 (s, 4H, N*H*); 7.31 (d, 8H, Ar*H*, *J* = 8.7 Hz); 7.20 (d, 8H, Ar*H*, *J* = 8.8 Hz); 7.03 (s, 2H, Ar*H*); 5.43 (s, 2H, Ar*H*); 1.21 (s, 36H, CH_3_) ppm. ^13^C­{^1^H} NMR (100 MHz, DMSO-*d*_6_) δ: 152.3; 148.5; 144.3; 136.7; 136.4; 128.6; 128.0; 125.3; 125.0; 122.4; 118.4; 113.7; 95.4; 33.8; 31.2 ppm. IR 3328; 3194; 3100; 3062; 2960; 2905; 2868; 1676; 1626; 1602; 1568; 1510 cm^–1^. HRMS (ESI) *m*/*z*: [M + Na]^+^ Calcd for C_68_H_72_N_16_O_12_Na 1327.5408; Found 1327.5404.

#### Bis-Ureido Azacalix[4]­arene **7**

Ureido derivative **7** was prepared according to the general procedure above by reaction of macrocyclic precursor **4b** 0.0665 g (0.12 mmol), which was dissolved in 0.5 mL of DMF. The precursor reacted with 90 μL of *tert-*butylphenyl isocyanate (0.49 mmol). After reaction competition, the product was precipitated in MeOH, filtered, and cleaned. The product was purified by using repetitive preparative TLC (eluent DCM:MeOH 40:1 (*v*:*v*)). The product was obtained as a dark brown powder (0.0771 g, 72%). mp 228 – 230 °C. ^1^H NMR (400 MHz, DMSO-*d*_6_) δ: 9.68 (s, 2H, N*H*); 9.53 (s, 2H, N*H*); 9.07 (s, 2H, Ar*H*); 8.97 (s, 2H, N*H*); 8.81 (s, 1H, Ar*H*); 8.11 (s, 2H, N*H*); 7.50 (t, 1H, Ar*H*, *J* = 8.0 Hz); 7.35 (d, 4H, Ar*H*, *J* = 8.9 Hz); 7.29 (d, 4H, Ar*H, J* = 9.0 Hz); 7.14–7.07 (m, Ar*H*, 3H); 7.01 (s, 1H, Ar*H*); 5.35 (s, 2H, ArH); 1.25 (s, 18H, −CH_3_) ppm. ^13^C­{^1^H} NMR (100 MHz, DMSO-*d*_6_) δ: 152.2; 148.5; 147.8; 144.4; 139.3; 136.71; 136.68; 131.8; 128.9; 128.1; 126.8; 126.6; 125.4; 124.9; 124.6; 121.5; 118.1; 112.4; 95.7; 33.9; 31.2 ppm. IR (ATR): 3321; 3198; 3100; 2959; 2930; 2907; 2866; 1696; 1627; 1568; 1511 cm^–1^. HRMS (ESI) *m*/*z*: [M + H]^+^ Calcd for C_46_H_45_N_12_O_10_ 925.3376; Found 925.3371, *m*/*z*: [M + Na]^+^ Calcd for C_46_H_44_N_12_O_10_Na 947.3196; Found 947.3179.

## Supplementary Material



## Data Availability

All data underlying this study are available in the published article and its Supporting Information.

## Notes

Copies of ^1^H, ^13^C NMR, IR spectra, and HRMS of new compounds; determination of binding efficiency; dilution experiments; and Job plot analyses (PDF)
