# Supplementary material for: Amplified Recognition of Basic Anions Induced by Cooperative Interaction of Ureido-Binding Sites Preorganized by Azacalix[4]arene Skeleton
Source: J Org Chem. 2026 Jan 16;91(4):1503–12. doi: 10.1021/acs.joc.5c02079 (PMC12865774; doi:10.1021/acs.joc.5c02079)
Supplement: Supplementary file 1 [file jo5c02079_si_001.pdf]

Supporting Information

Amplified recognition of basic anions induced by cooperative interaction of ureido-binding sites preorganised by azacalix[4]arene skeleton

Karolína Salvadori[a,b,c*]; Pavel Matějka[d], Pavel Lhoták[e], Olivier Siri[b*]

a Department of Analytical Chemistry, University of Chemistry and Technology Prague, Technická 5, Prague 6 16628, Czech Republic, salvadok@vscht.cz

b Aix-Marseille Université, CNRS UMR 7325 Centre Interdisciplinaire de Nanoscience de Marseille (CINaM), Campus de Luminy, Marseille cedex 09 13288, France; olivier.siri@univ-amu.fr

c Department of Bioorganic Chemistry and Biomaterials, Institute of Chemical Process Fundamentals of the CAS, v.v.i., Rozvojová 135, Prague 6 16502, Czech Republic

d Department of Physical Chemistry, University of Chemistry and Technology Prague, Technická 5, Prague 6 16628, Czech Republic

e Department of Organic Chemistry, University of Chemistry and Technology Prague, Technická 5, Prague 6 16628, Czech Republic

Contents

1. Evaluation of self-aggregation S2
2. Interaction with anions and DBU S7
   2.1 UV-Vis studies S7
   2.2 NMR Studies S12
   2.3 Stoichiometry of studied complexes S15
   2.4 Binding efficiency S16
3. Spectral records S23
   3.1 Platforms S23
   3.2 Ureido-derivatives S35

1. Evaluation of self-aggregation

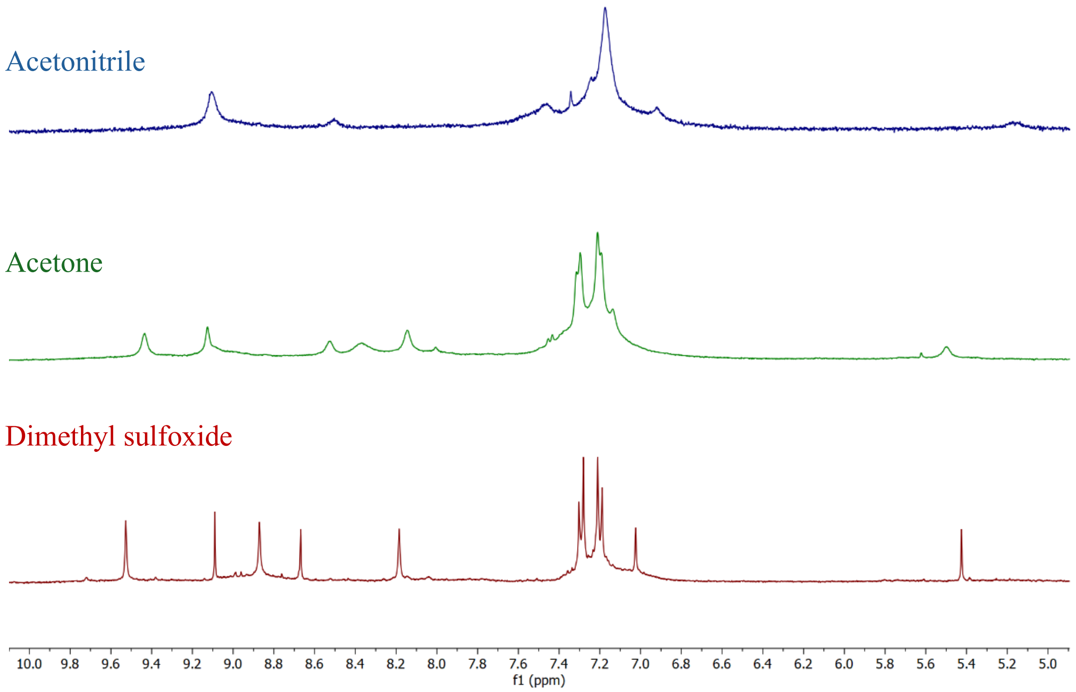

Fig. S1: Comparison of aromatic an N*H* region of receptor **6** (1.3 mM) in different solvents.

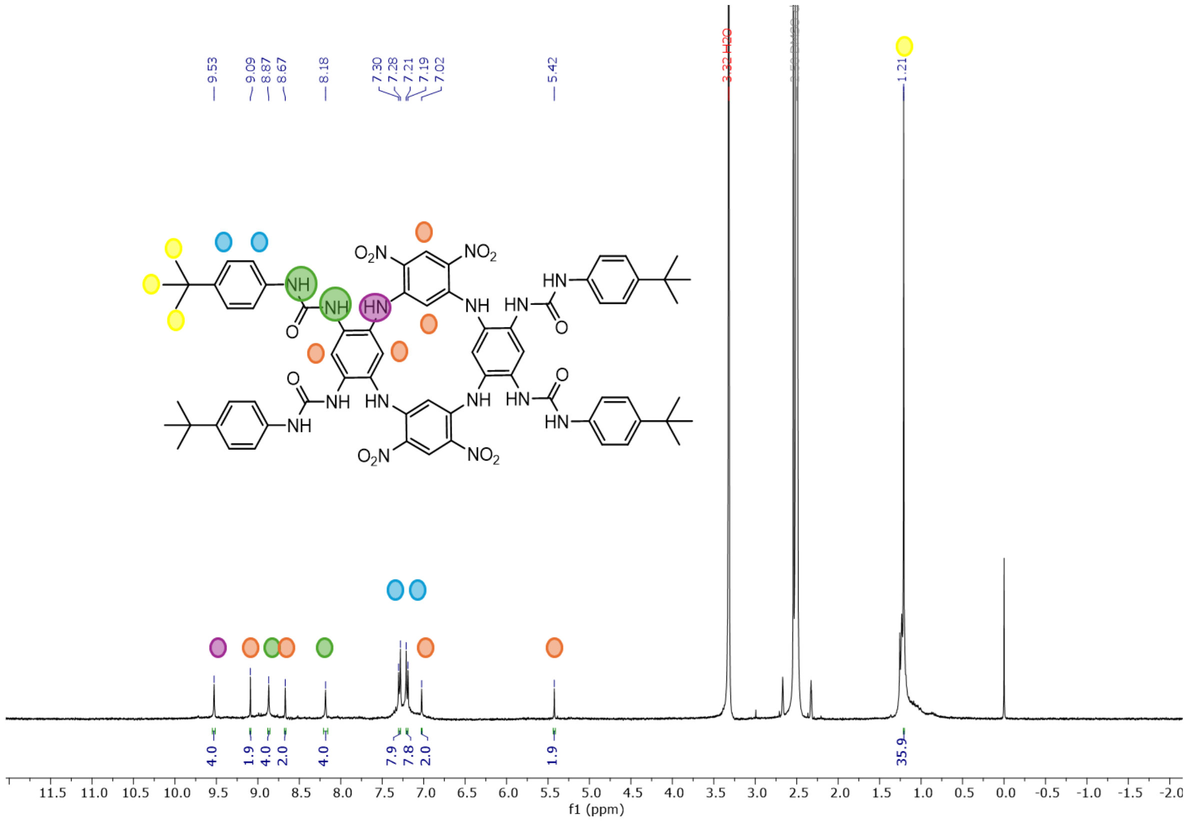

Fig. S2: Discussed ^{1}H NMR of compound **6** (DMSO-d_6, 400 MHz).

Table S1: Values of constants K (K_D - dimerization constant; K_e - elongation constant) corresponding to the studied receptors in DMSO-d_6.

Receptor	K_e [a]	K_D [b]
**5a**	60	30
**6**	36	18
**7**	20	10

[a] Error, when estimated, was < 10%; [b] Error, when estimated, was < 5%.

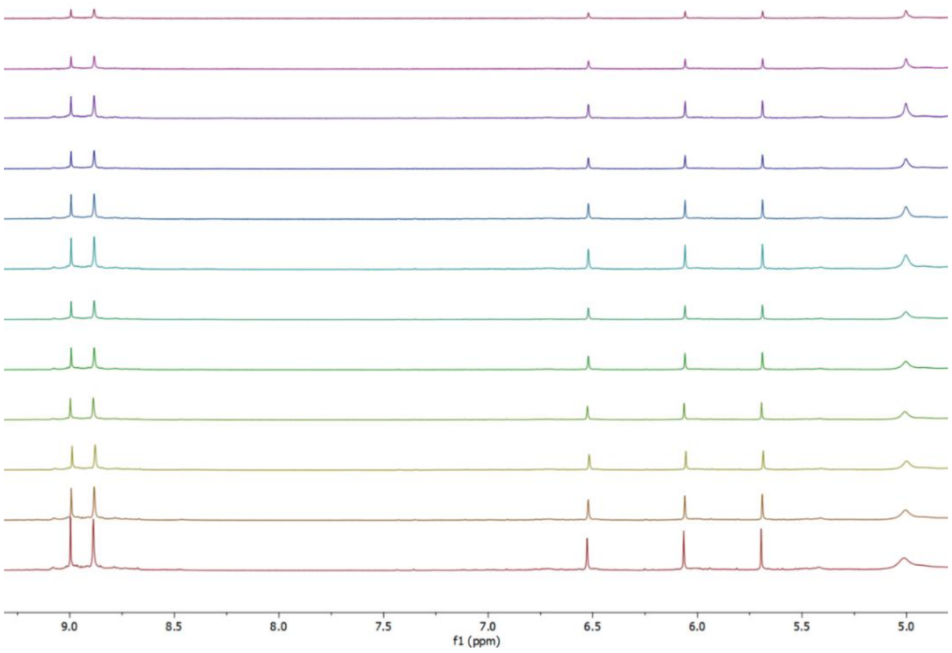

Fig. S3: Part of ^{1}H NMR spectra for N*H* and aromatic region of **4a** in DMSO-d_6. The studied concentration range corresponds to 26.3–0.3 mM.

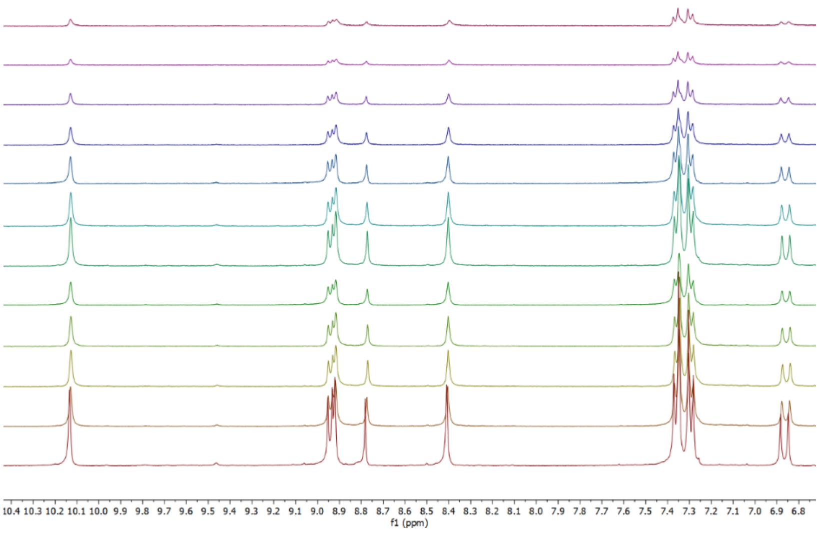

Fig. S4: Part of ^{1}H NMR spectra for urea´s hydrogen N*H* and aromatic region of **5b** in DMSO-d_6. The studied concentration range corresponds to 26.7–0.3 mM.

Table S2: Chemical shift of urea´s N*H* protons during dilution of **5a** in DMSO-d_6.

c (**5a**) mM	δ_{NH-A} (ppm)	δ_{NH-B} (ppm)
26.0	9.0075	8.4702
17.3	8.9968	8.4589
13.0	8.9873	8.4492
10.4	8.9812	8.4429
8.67	8.9762	8.4377
6.94	8.9695	8.4308
5.78	8.9662	8.4273
3.86	8.9576	8.4187
2.57	8.9505	8.4122
1.29	8.9417	8.4041
0.643	8.9364	8.4003
0.321	8.9315	8.3980

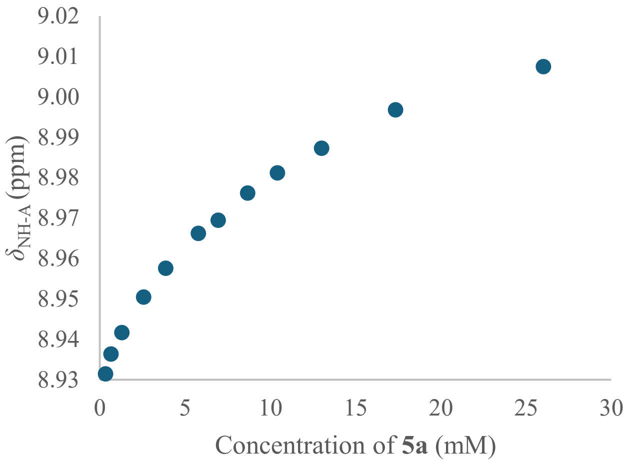

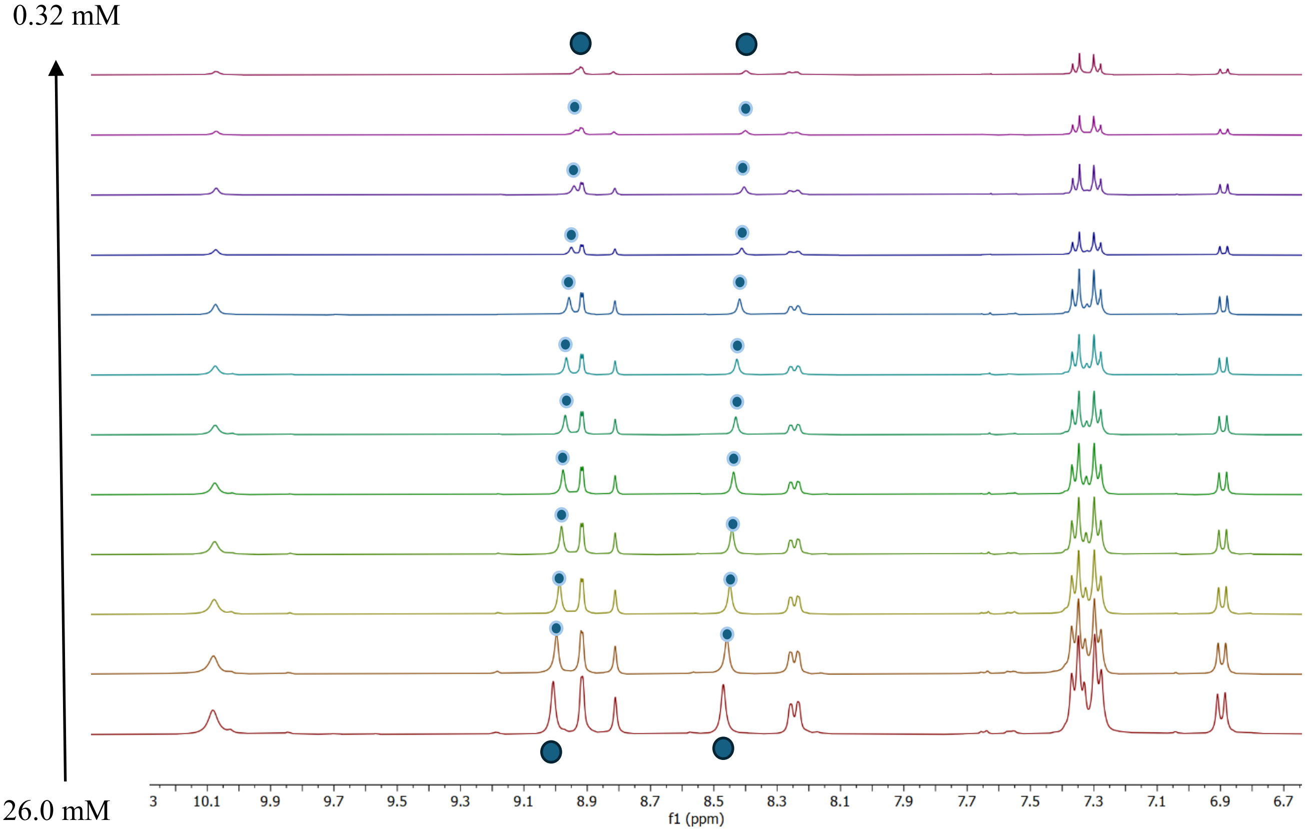

Fig. S5: Part of ^{1}H NMR spectra for urea´s hydrogen N*H* and aromatic region of **5a** in DMSO-d_6. The studied concentration range corresponds to 26–0.3 mM.

Link: http://app.supramolecular.org/bindfit/view/19bdd670-4e77-4ac5-b834-752613dd0321

Table S3: Chemical shift of urea´s N*H* protons during dilution of receptor **6** in DMSO-d_6.

c (**6**) mM	$\delta_{NH\text{-}A}$ (ppm)	$\delta_{NH\text{-}B}$ (ppm)
26.1	8.9470	8.2300
17.4	8.9328	8.2208
13.0	8.9213	8.2137
10.4	8.9130	8.2085
8.70	8.9081	8.2057
6.96	8.8992	8.2006
5.80	8.8933	8.1974
3.87	8.8848	8.1924
2.58	8.8773	8.1887
1.29	8.8708	8.1847
0.64	8.8649	8.1823
0.32	8.8600	8.1789

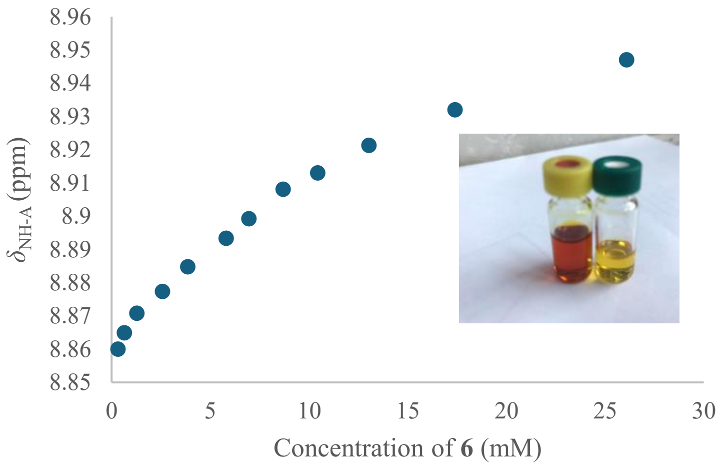

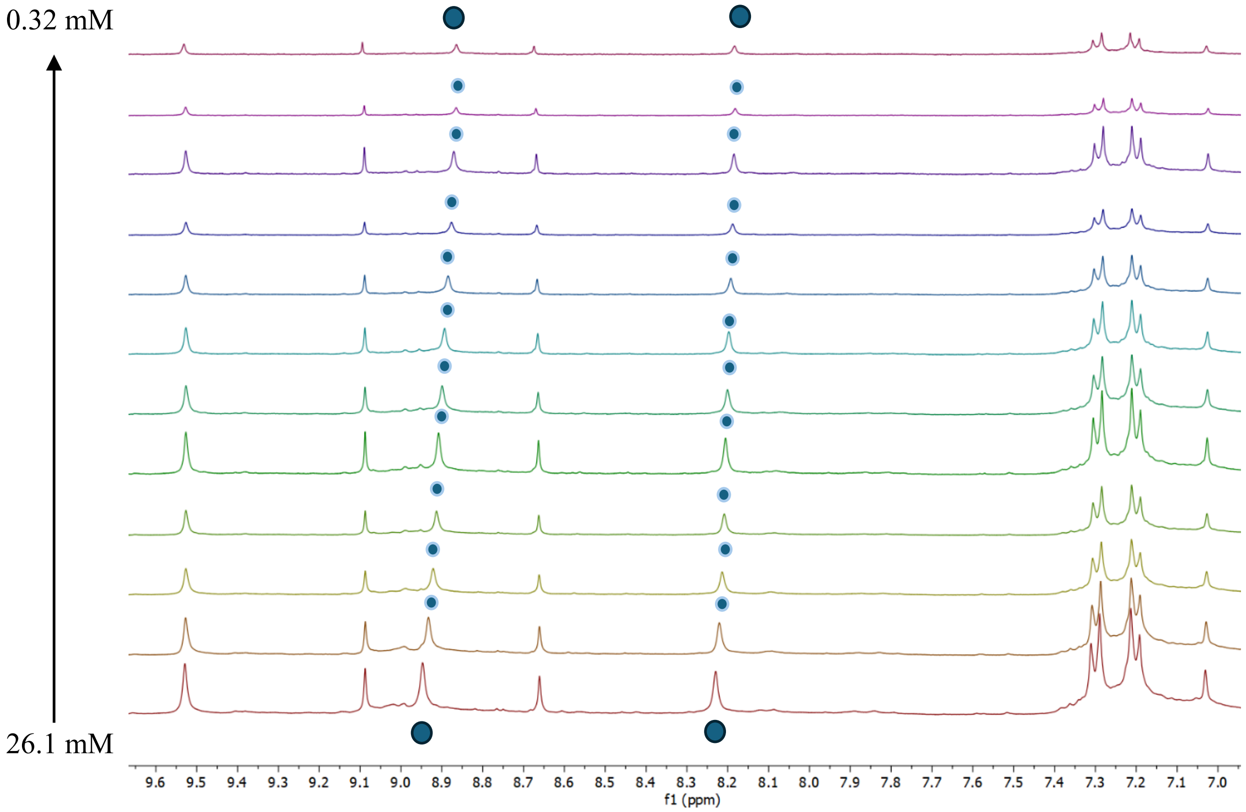

Fig. S6: Part of ^{1}H NMR spectra for urea´s hydrogen N*H* and aromatic region of **6** in DMSO-d_6. The studied concentration range corresponds to 26–0.3 mM.

Link: http://app.supramolecular.org/bindfit/view/991af64a-2e23-4945-b338-ad0b8acea168

Table S4: Chemical shift of urea´s N*H* protons during dilution of receptor **7** in DMSO-d_6.

c (**7**) mM	δ_{NH-A} (ppm)	δ_{NH-B} (ppm)
24.4	9.0510	8.1660
16.3	9.0309	8.1508
12.2	9.0205	8.1426
9.76	9.0133	8.1372
8.13	9.0104	8.1344
6.50	9.0062	8.1315
5.42	9.0020	8.1282
3.61	8.9958	8.1233
2.41	8.9902	8.1191
1.20	8.9834	8.1140
0602	8.9786	8.1100
0.301	8.9750	8.1072

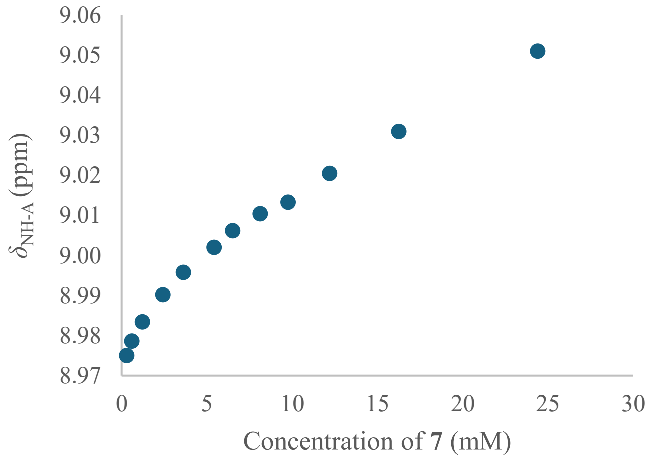

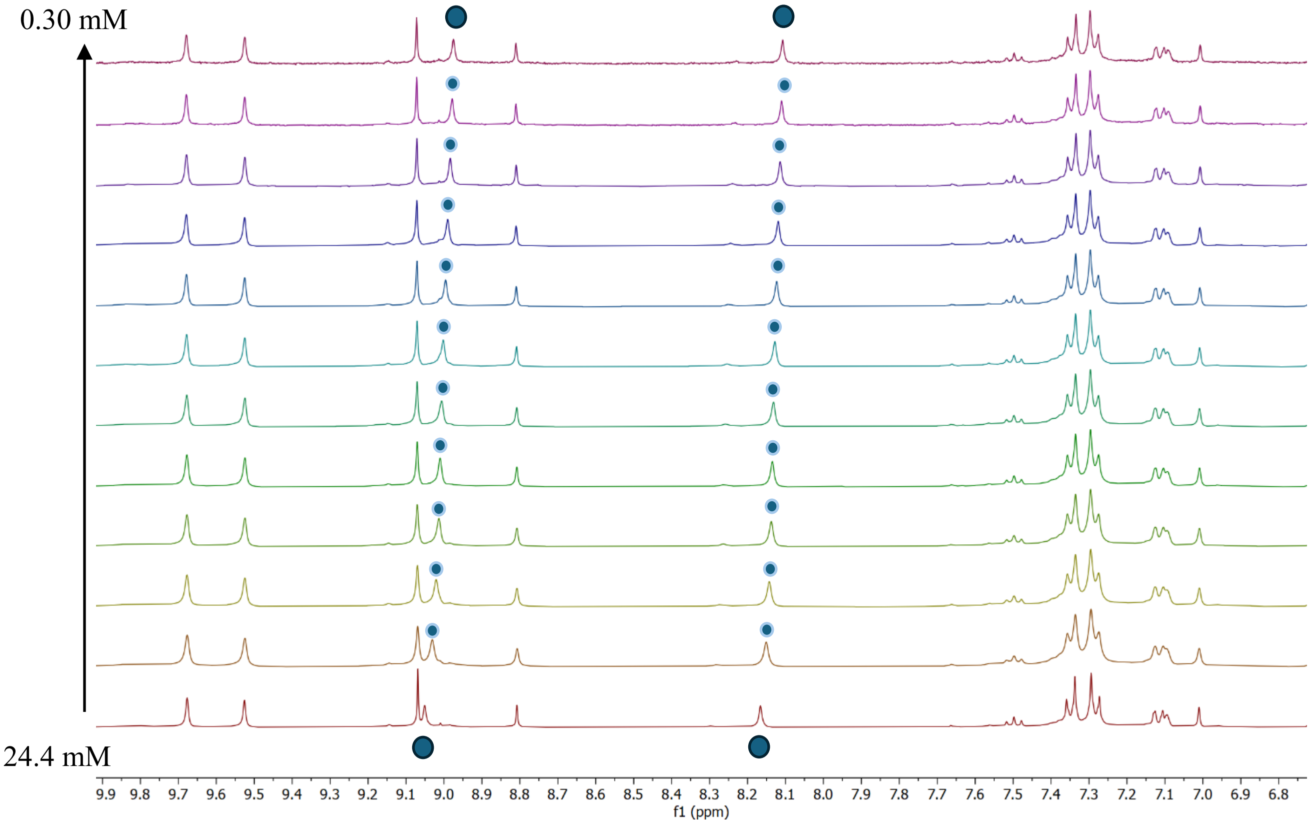

Fig. S7: Part of ^{1}H NMR spectra for urea´s hydrogen N*H* and aromatic region of **7** in DMSO-d_6. The studied concentration range corresponds to 24–0.3 mM.

Link: http://app.supramolecular.org/bindfit/view/b01d708f-5135-4b62-8ade-7e0ea0690731

2. Interaction with anions and DBU

2.1 UV-Vis studies

c (**6**) mM	c ($H_2PO_4^-$) mM	A_{370} (A.U.)
0.224	0	0.921
0.224	0.108	0.894
0.224	0.211	0.875
0.224	0.311	0.863
0.224	0.406	0.857
0.224	0.499	0.850
0.224	0.588	0.843
0.224	0.674	0.839
0.224	0.757	0.837
0.224	0.914	0.829
0.224	1.06	0.824
0.224	1.23	0.815
0.224	1.39	0.812
0.224	1.57	0.809

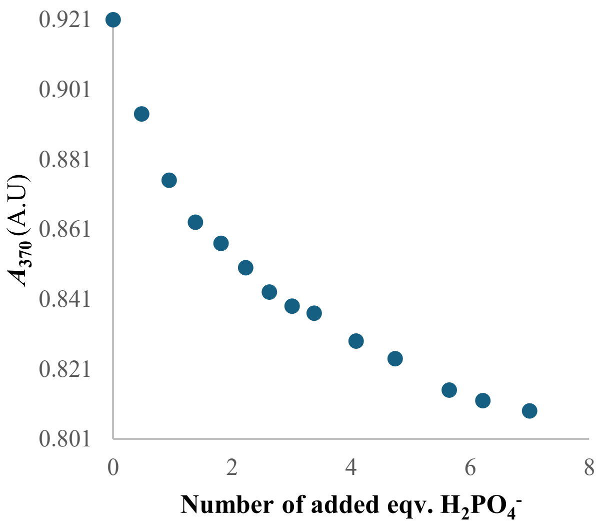

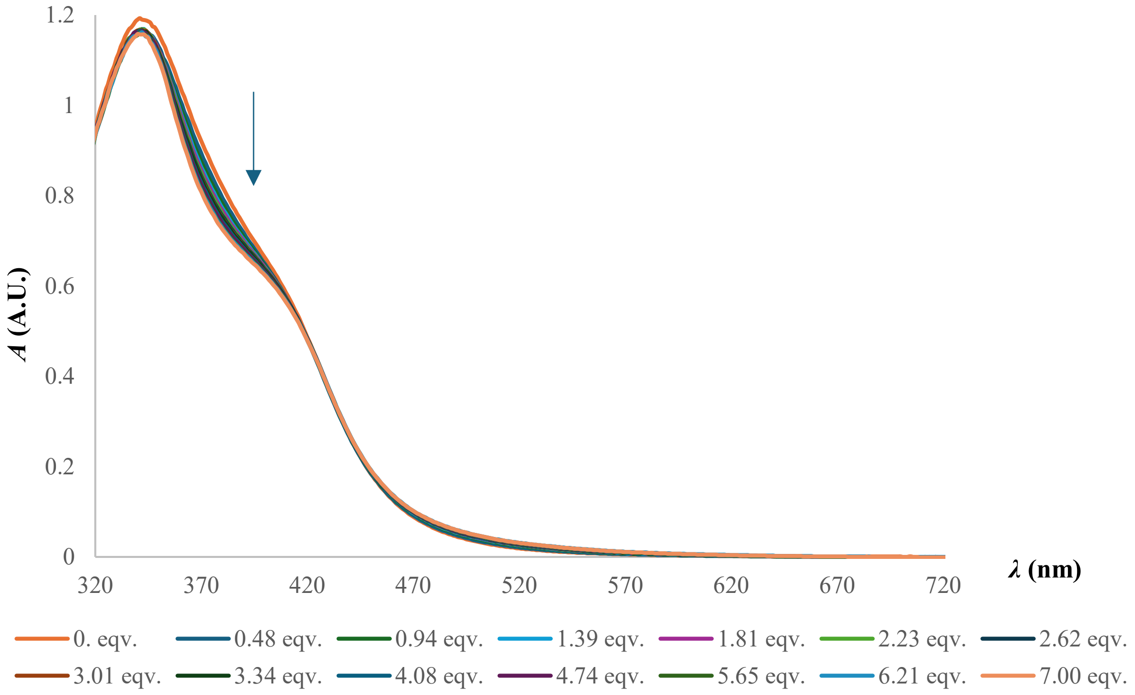

Fig. S8: Records of UV-Vis obtained for receptor **6** (0.22 mM in DMSO) titrated with $TBAH_2PO_4$ (final concentration 1.57 mM) in a cuvette with 1-mm pathlength. The arrow indicates the decrease of absorbance.

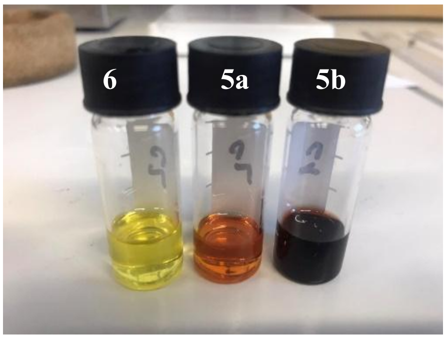

With $H_2PO_4^-$

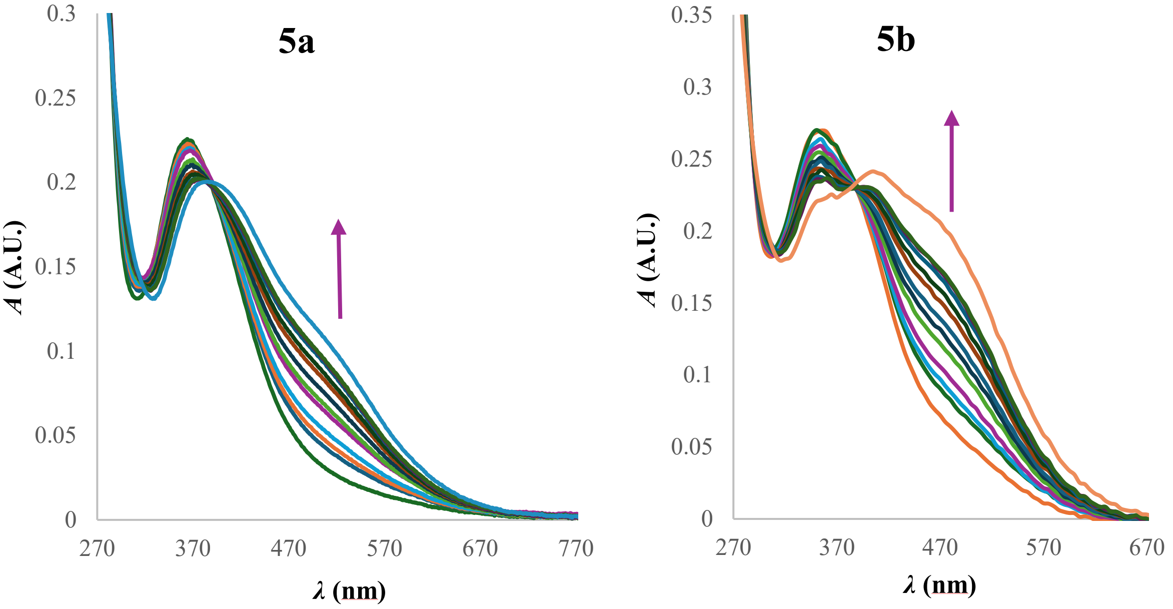

Fig. S9: The differences in UV-Vis records and colour changes induced by adding $H_2PO_4^-$ (in DMSO) obtained for acyclic ureas (**5a** and **5b**).

Records for **5a**: *c*(**5a**) = 0.12 mM $c(H_2PO_4^-)_{final}$ = 2.32 mM in a cuvette with 1-mm pathlength.

Records for **5b**: *c*(**5b**) = 0.11 mM, $c(H_2PO_4^-)_{final}$ = 2.05 mM in a cuvette with 1-mm pathlength.

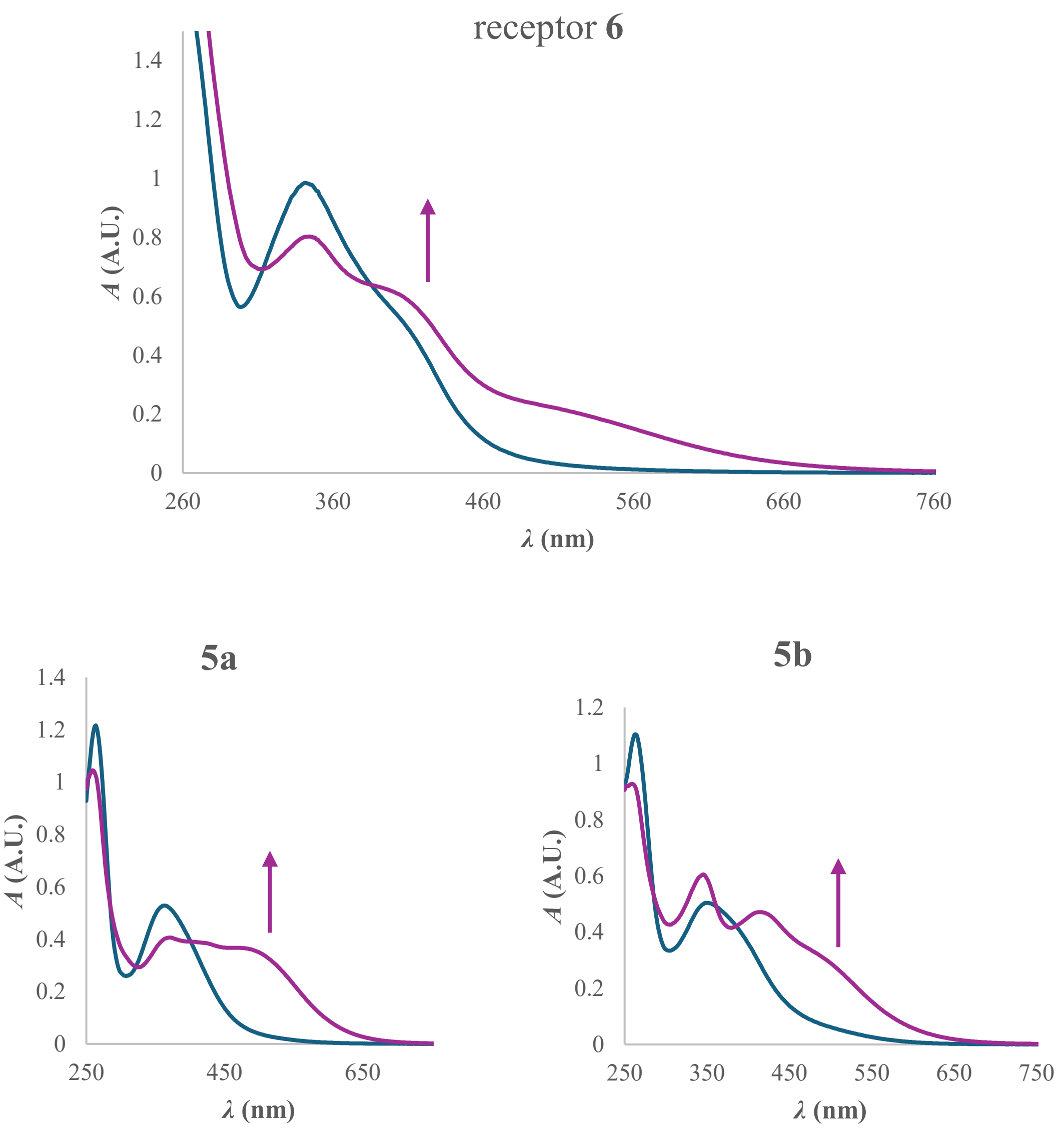

Fig. S10: The UV-Vis records and changes induced by adding DBU (in DMSO) obtained for acyclic ureas (**5a** and **5b**) and tetra-urea derivatives **6**. Blue curve corresponds to the original spectra, pink to the records after the introduction of DBU (in excess).

Records for **6**: *c*(**6**) = 0.20 mM, *c*(DBU) = 1.69 mM in a cuvette with 1-mm pathlength.

Records for **5a**: *c*(**5a**) = 0.22 mM *c*(DBU) = 1.87 mM in a cuvette with 1-mm pathlength.

Records for **5b**: *c*(**5b**) = 0.21 mM, *c*(DBU) = 1.99 mM in a cuvette with 1-mm pathlength.

c (**5a**) mM	c (DBU) mM	A_{500} (A.U.)
0.217	0	0.039
0.217	0.0824	0.093
0.217	0.162	0.135
0.217	0.238	0.171
0.217	0.382	0.230
0.217	0.516	0.276
0.217	0.701	0.320
0.217	0.970	0.350
0.217	1.11	0.358
0.217	1.20	0.361
0.217	1.58	0.376
0.217	1.87	0.431

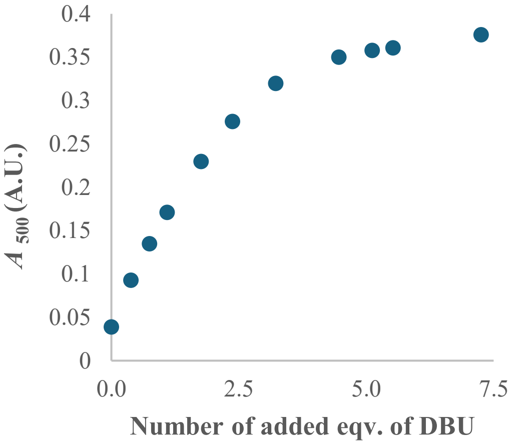

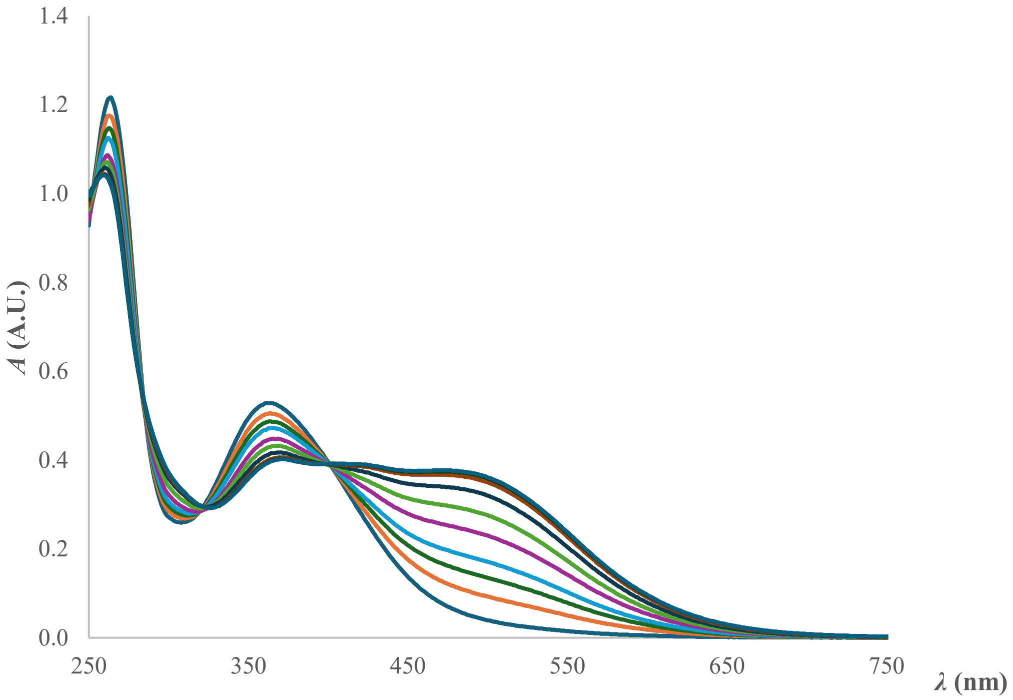

Fig. S11: Records of UV-Vis obtained for **5a** (0.22 mM in DMSO) titrated with DBU (final concentration 1.87 mM) in a cuvette with 1-mm pathlength.

c (**5b**) mM	*c* (DBU) mM	A_{422} (A.U.)
0.207	0	0.246
0.207	0.0876	0.348
0.207	0.172	0.370
0.207	0.253	0.394
0.207	0.406	0.419
0.207	0.549	0.432
0.207	0.744	0.444
0.207	1.03	0.456
0.207	1.18	0.460
0.207	1.28	0.461
0.207	1.53	0.466
0.207	1.99	0.470

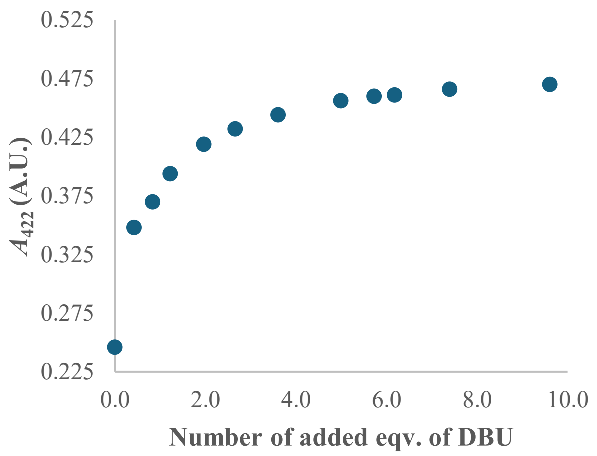

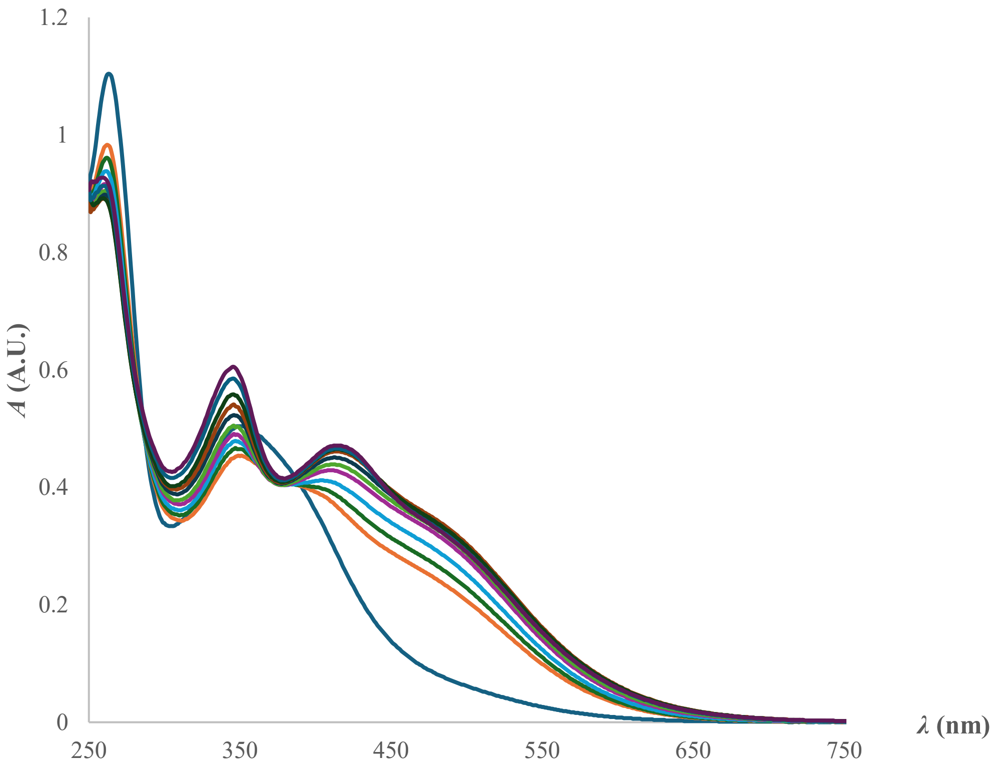

Fig. S12: Records of UV-Vis obtained for **5b** (0.21 mM in DMSO) titrated with DBU (final concentration 1.99 mM) in a cuvette with 1-mm pathlength.

2.2 NMR Studies

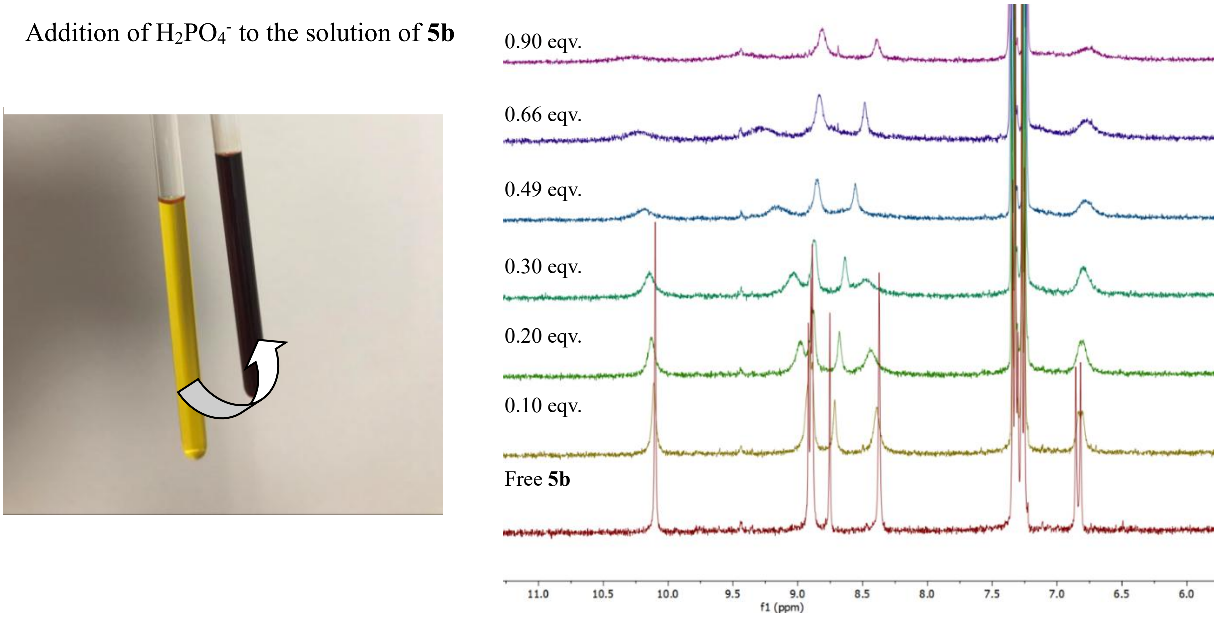

Fig. S13: A series of ^{1}H NMR spectra (DMSO-d_6, 400 MHz – aromatic and N*H* region) of **5b** (1.95 mM) after the addition of $TBAH_2PO_4$ (final concentration 1.76 mM).

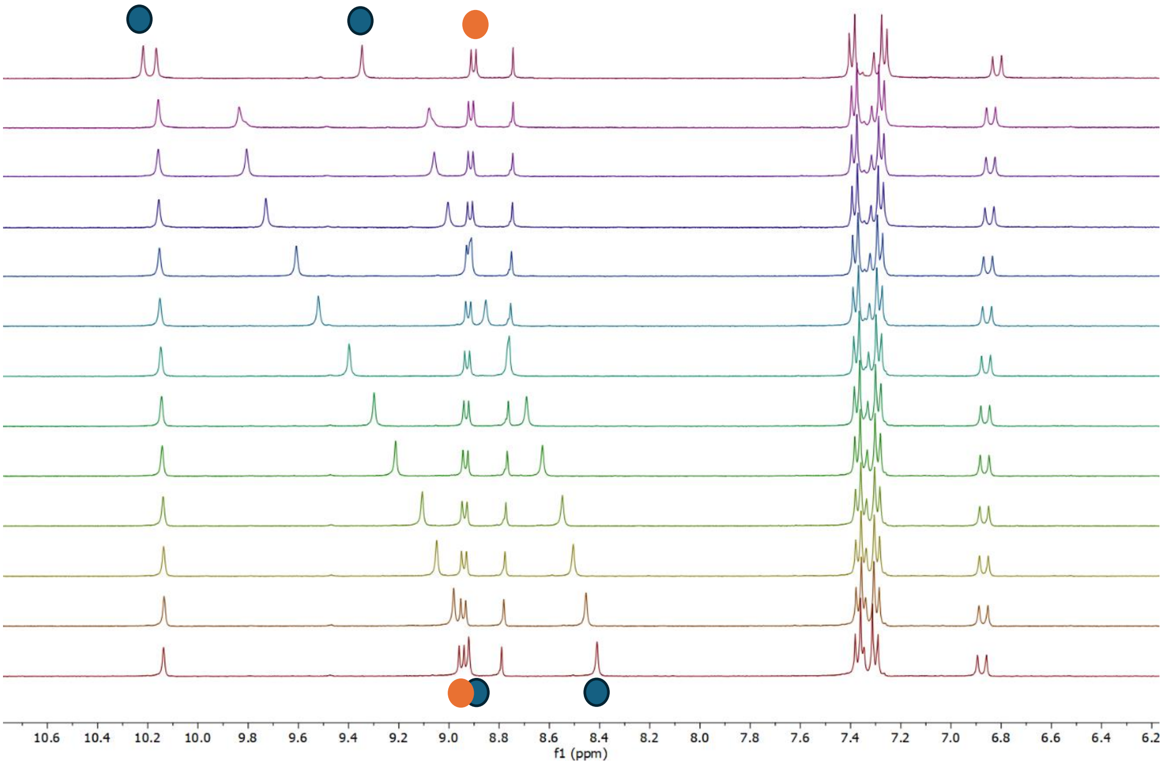

Fig. S14: Part of aromatic and N*H* region during ^{1}H NMR titration of receptor **5b** (4.14 mM) with TBACl (final concentration 86.4 mM) with highlighted urea N*H* hydrogens (blue) and diagnostic aromatic signals corresponding to skeleton (orange).

Link: http://app.supramolecular.org/bindfit/view/b957f31b-71fa-4cee-ab02-65d9ed183741

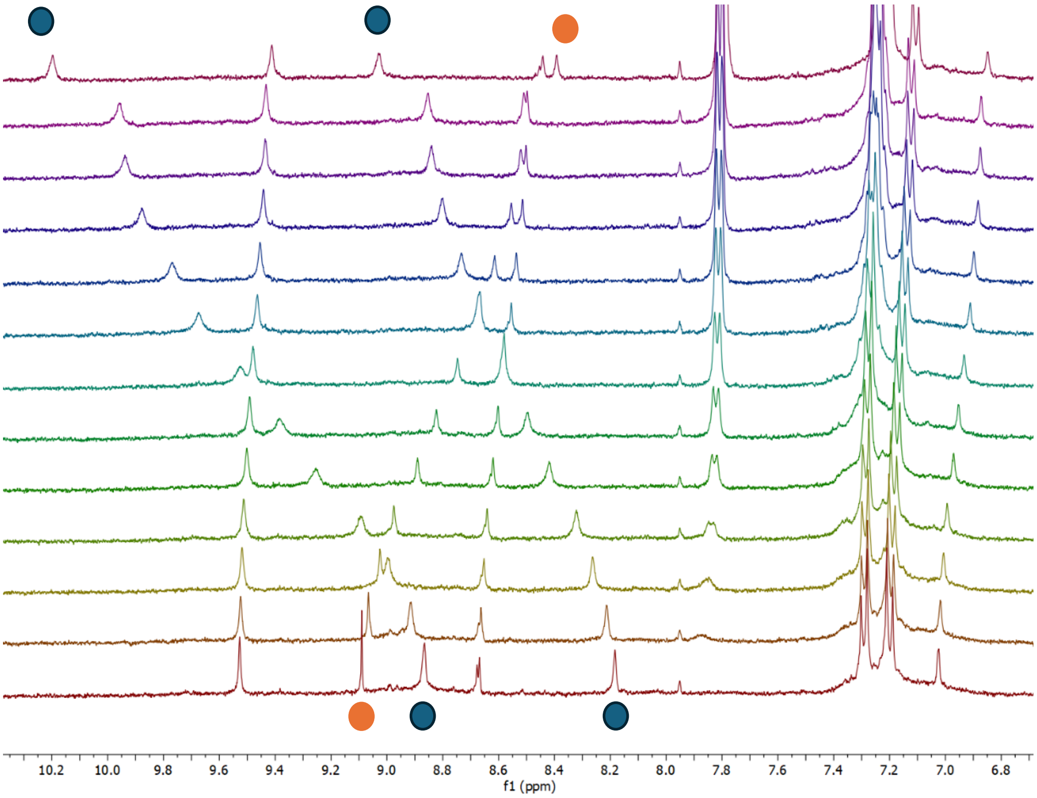

Fig. S15: Part of aromatic and N*H* region during ^{1}H NMR titration of receptor **6** (1.20 mM) with TBABzO^{-} (final concentration 9.90 mM) with highlighted urea N*H* hydrogens (blue) and diagnostic aromatic signals corresponding to macrocycle (orange).

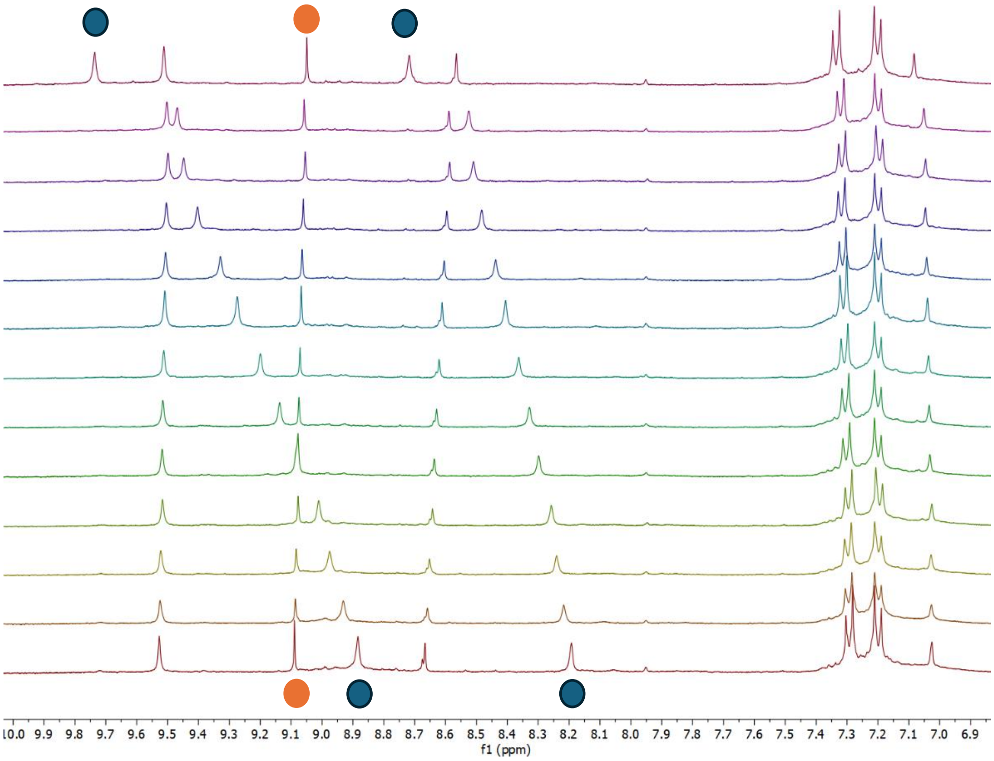

Fig. S16: Part of aromatic and N*H* region during ^{1}H NMR titration of receptor **6** (3.62 mM) with TBACl^{-} (final concentration 100 mM) with highlighted urea N*H* hydrogens (blue) and diagnostic aromatic signals corresponding to macrocycle (orange).

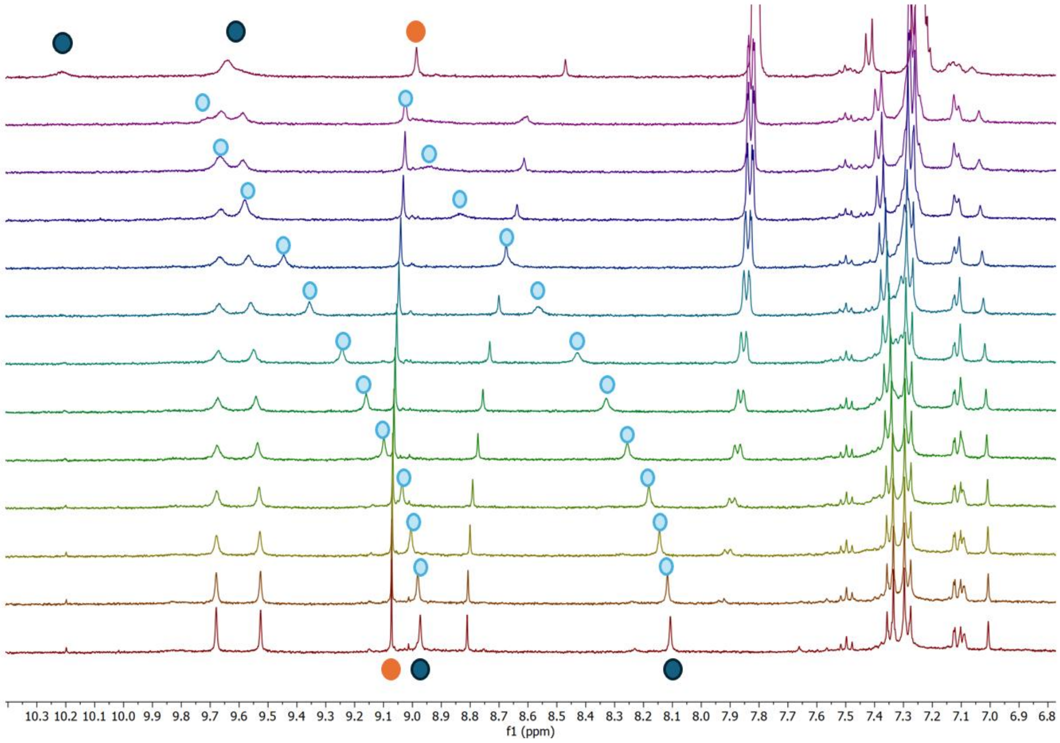

Fig. S17: Part of aromatic and N*H* region during ^{1}H NMR titration of receptor **7** (1.20 mM) with TBABzO^{-} (final concentration 4.84 mM) with highlighted urea N*H* hydrogens (blue) and diagnostic aromatic signals corresponding to macrocycle (orange).

2.3 Stoichiometry of studied complexes

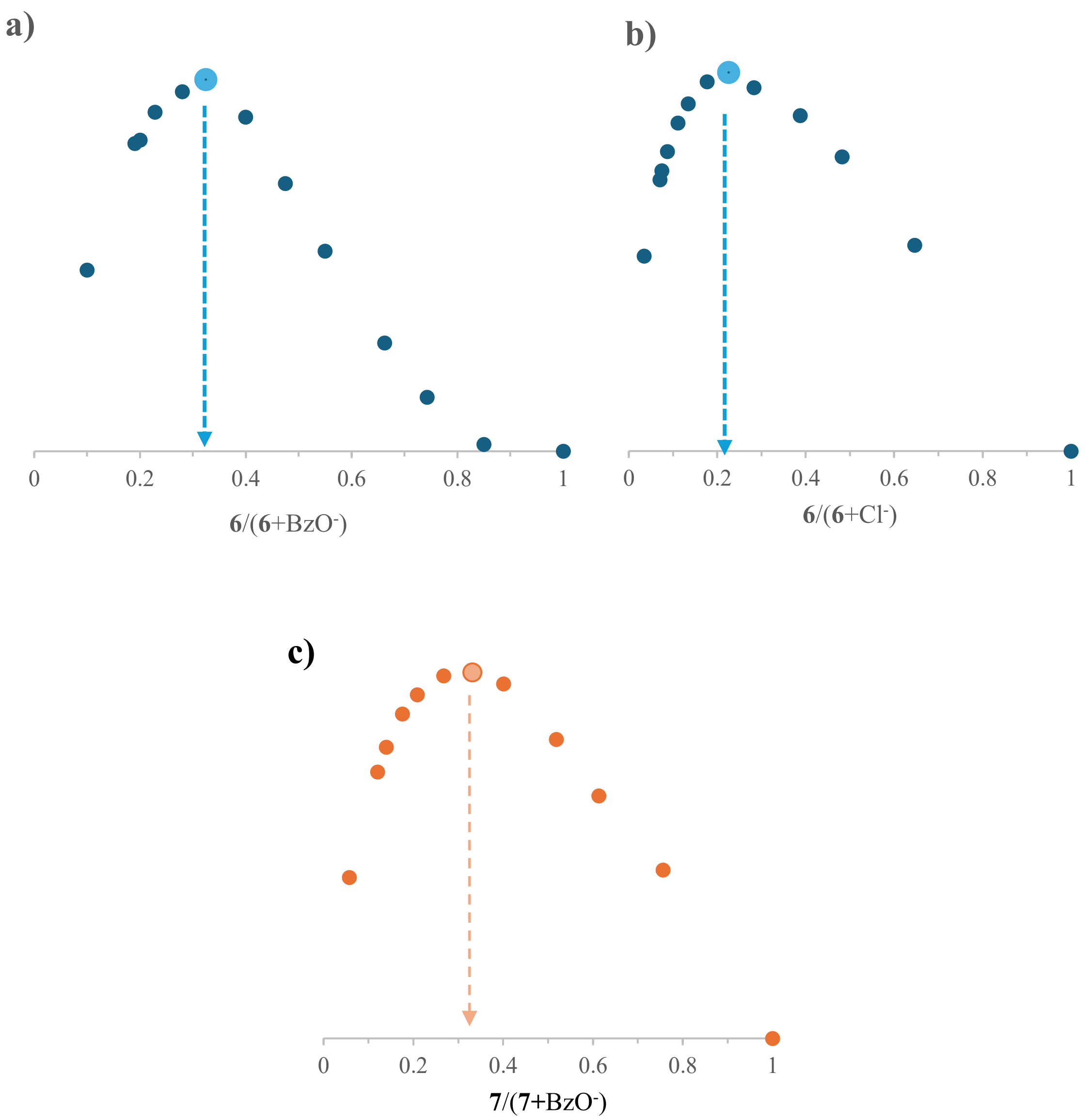

Fig. S18: Job plot (^{1}H NMR, 400 MHz, DMSO-d_6, 298 K) recorded for receptor **6** (a) with BzO^- and (b) with Cl^- anions; and its model macrocyclic precursor **7** with BzO^- (c).

2.4 Binding efficiency

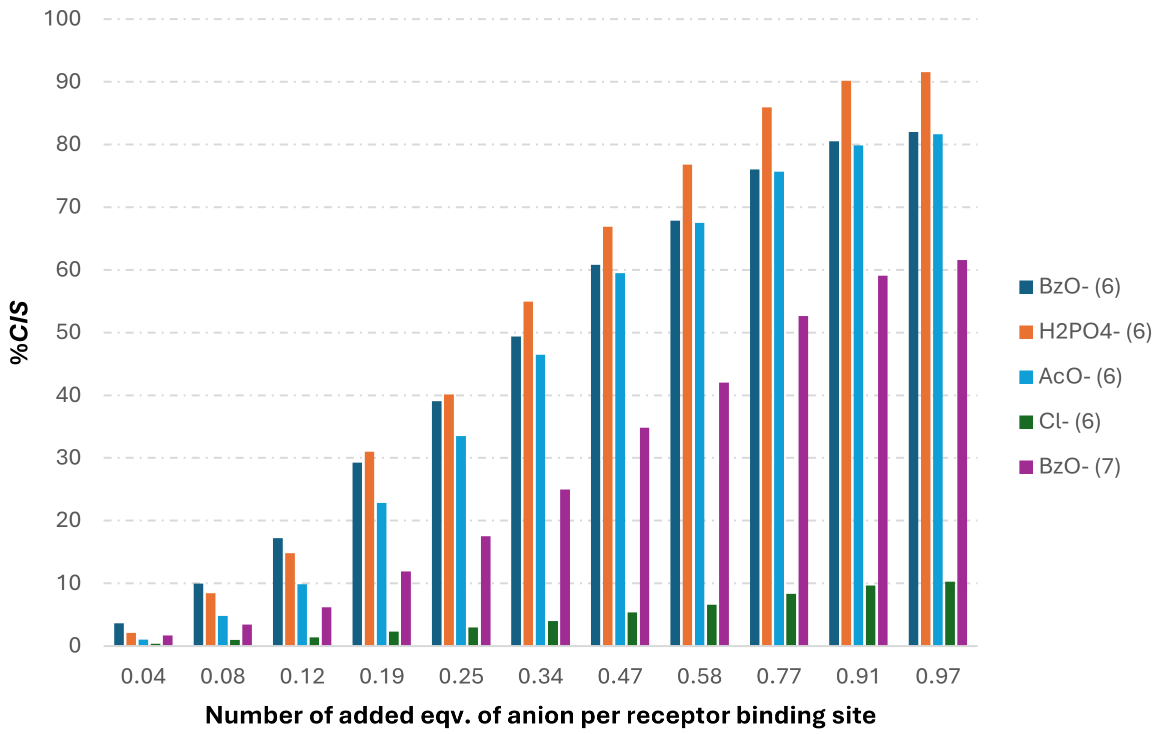

Fig. S19: Comparison of binding efficiency of receptors **6** (1.2 mM, DMSO-d_6) and **7** (2.4 mM, DMSO-d_6) towards several anion in the form of their TBA salts.

Table S5: Summarized values of apparent association constants K_{As} (1:1) and overall association constants β of receptors (**5b**, **6**, and **7**) were determined by ^{1}H NMR in DMSO-d_6 with a series of anions in the form of TBA salts.

Receptor	Anion	Description	K_{As}[a]	β
**5b**	$H_2PO_4^-$	deprotonation	-	--
	Cl^-	complexation (1:2)	45	$5.06 \cdot 10^2$
**6**	$H_2PO_4^-$	complexation (1:2)	5 060	$6.40 \cdot 10^6$
	BzO^-	complexation (1:2)	2 520	$1.59 \cdot 10^6$
	AcO^-	complexation (1:2)	1 950	$9.50 \cdot 10^5$
	Cl^-	complexation (1:4)	More complex equilibria	
**7**	BzO^-	Complexation (1:2)	660	$1.09 \cdot 10^5$

[a] Error, when estimated, was < 20 %.

[b] Were evaluated for 1:2 stoichiometry, where $\beta = K_{As}$ (1:1) × K_{As} (1:2), and K_{As} (1:2) = K_{As} (1:1)/4.

* The results for 1:4 stoichiometry has no suitable mathematical description for fitting.

c (**6**) mM	c (BzO^-) mM	Equiv. per binding site	δ_{NH} (ppm)	% *CIS*
1.20	0	0	8.8731	0
1.20	0.194	0.04	8.9217	3.65
1.20	0.381	0.08	9.0056	9.94
1.20	0.560	0.12	9.1028	17.2
1.20	0.900	0.19	9.2632	29.3
1.20	1.22	0.25	9.3936	39.1
1.20	1.65	0.35	9.5311	49.4
1.20	2.29	0.48	9.6837	60.8
1.20	2.83	0.59	9.7777	67.9
1.20	3.71	0.78	9.8860	76.0
1.20	4.40	0.92	9.9460	80.5
1.20	4.69	0.98	9.9662	82.0
1.20	9.90	2.06	10.2059	100

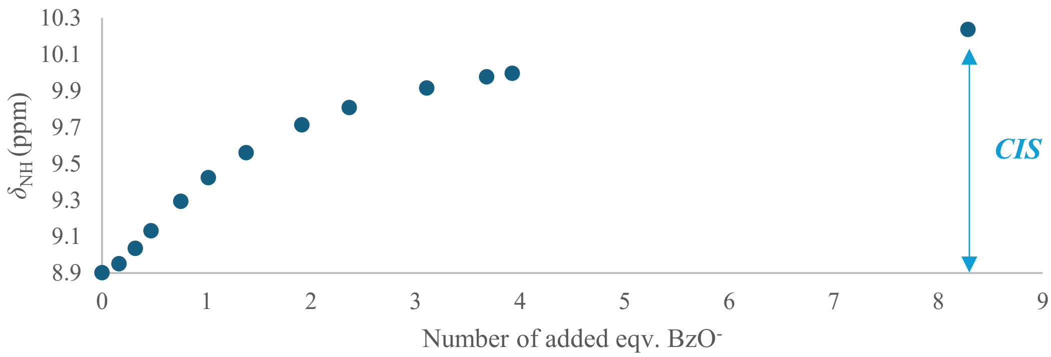

Fig. S20a: Binding isotherm obtained during ^{1}H NMR titration of **6** (1.20 mM, DMSO-d_6) with solution of guest (containing **6**:1.20 mM; TBABzO: 9.90 mM, DMSO-d_6).

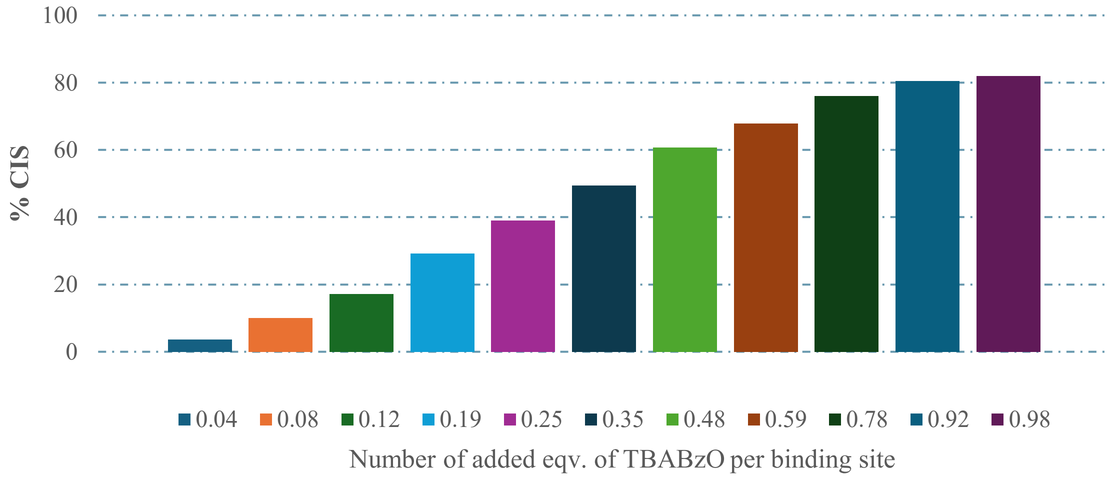

Fig. S20b: The evaluation of binding efficiency of receptor **6** with TBABzO. The complexation ability is expressed by % of *CIS* (y-axes), which depends on number of added equiv. of TBABzO per binding unit.

Link: http://app.supramolecular.org/bindfit/view/797e7a88-908f-46eb-8006-06ede6e0f2ba

c (**6**) mM	c ($H_2PO_4^-$) mM	Equiv. per binding site	δ_{NH} (ppm)	% *CIS*
1.17	0	0	8.87	0
1.17	0.186	0.04	8.90	2.11
1.17	0.365	0.08	8.99	8.45
1.17	0.538	0.11	9.08	14.8
1.17	0.864	0.18	9.31	31.0
1.17	1.17	0.25	9.44	40.1
1.17	1.58	0.34	9.65	54.9
1.17	2.19	0.47	9.82	66.9
1.17	2.72	0.58	9.96	76.8
1.17	3.56	0.76	10.09	85.9
1.17	4.22	0.90	10.15	90.1
1.17	4.50	0.96	10.17	91.5
1.17	9.50	2.03	10.29	100

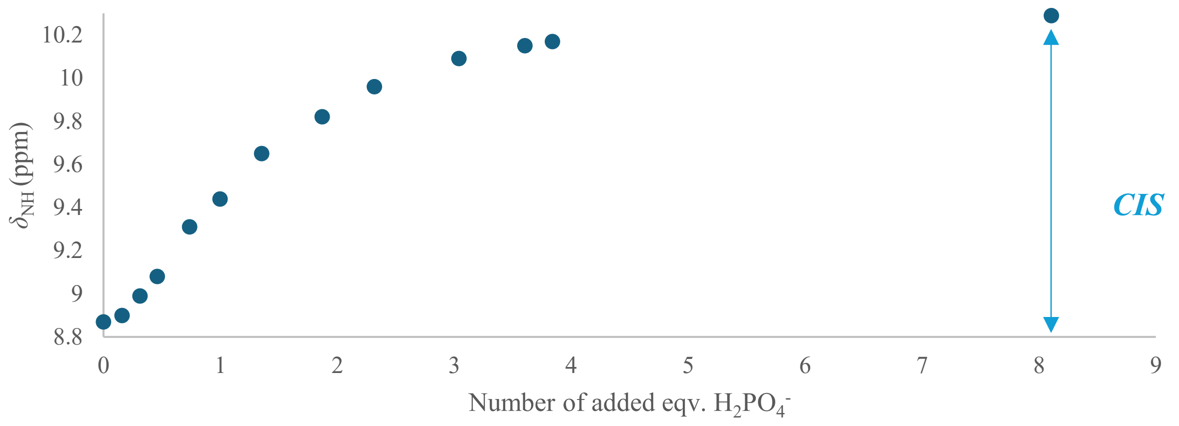

Fig. S21a: Binding isotherm obtained during ^{1}H NMR titration of **6** (1.17 mM, DMSO-d_6) with solution of guest (containing **6**: 1.17 mM; $TBAH_2PO_4$: 9.50 mM, DMSO-d_6).

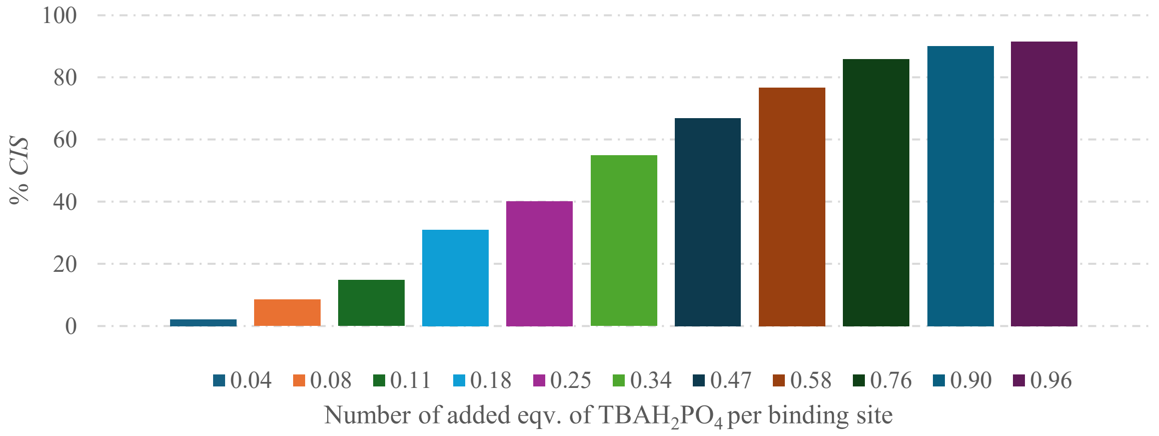

Fig. S21b: The evaluation of binding efficiency of receptor **6** with $TBAH_2PO_4$. The complexation ability is expressed by % of *CIS* (y-axes), which depends on number of added equiv. of $TBAH_2PO_4$ per binding unit.

Link: http://app.supramolecular.org/bindfit/view/4932e711-9dca-4707-b9b2-19f1b17eec06

c (**6**) mM	*c* (AcO^-) mM	Equiv. per binding site	δ_{NH} (ppm)	% *CIS*
1.19	0	0	8.8689	0
1.19	0.187	0.04	8.8820	1.01
1.19	0.367	0.08	8.9309	4,80
1.19	0.541	0.11	8.9960	9.85
1.19	0.868	0.18	9.1638	22.8
1.19	1.17	0.25	9.3013	33.5
1.19	1.59	0.34	9.4687	46.5
1.19	2.20	0.46	9.6368	59.5
1.19	2.73	0.57	9.7398	67.5
1.19	3.58	0.75	9.8455	75.7
1.19	4.25	0.89	9.8996	79.9
1.19	4.52	0.95	9.9225	81.6
1.19	9.55	2.01	10.1596	100

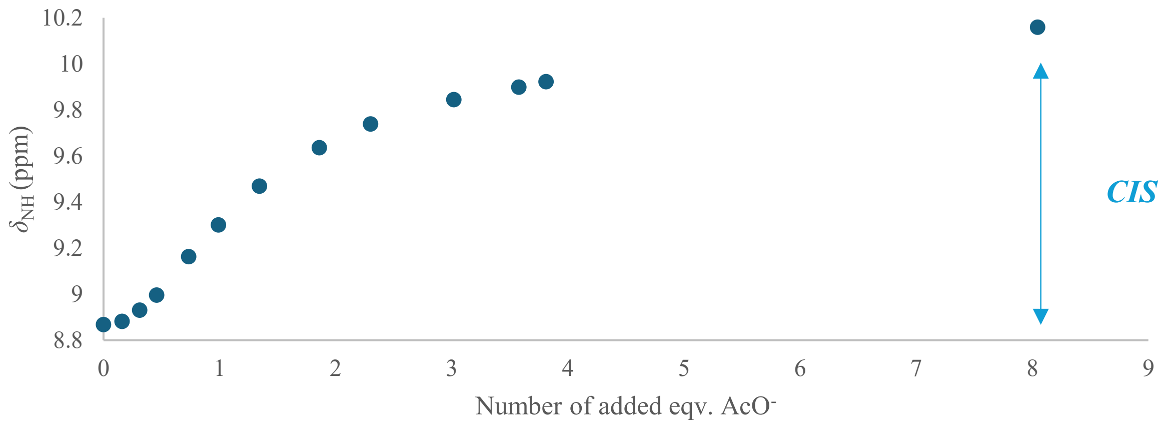

Fig. S22a: Binding isotherm obtained during ^{1}H NMR titration of **6** (1.19 mM, DMSO-d_6) with solution of guest (containing **6**: 1.17 mM; TBAAcO: 9.55 mM, DMSO-d_6).

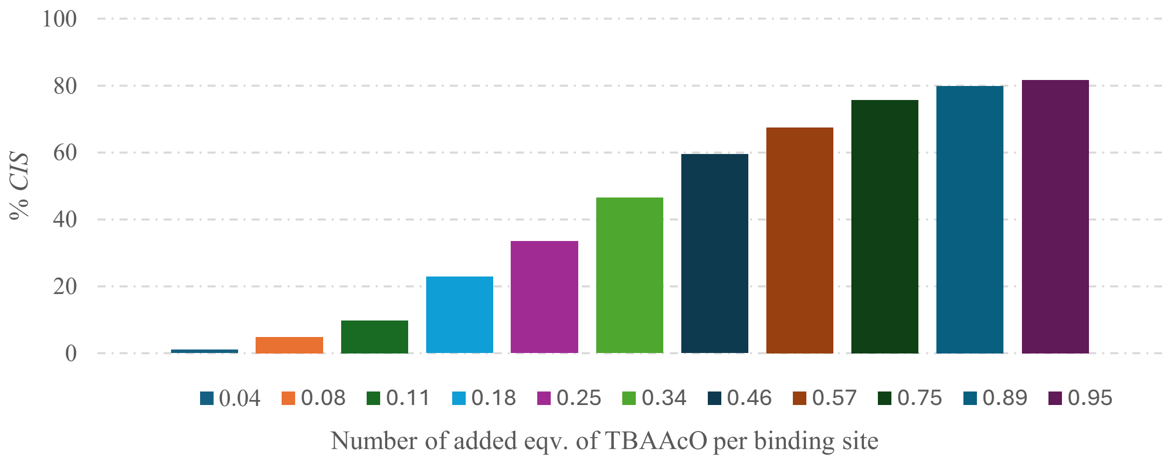

Fig. S22b: The evaluation of binding efficiency of receptor **6** with TBAAcO. The complexation ability is expressed by % of *CIS* (y-axes), which depends on number of added equiv. of TBAAcO per binding unit.

Link: http://app.supramolecular.org/bindfit/view/21022b1e-a665-4e39-b474-84230248bf03

c (**6**) mM	*c* (Cl^-) mM	Equiv. per binding site	δ_{NH} (ppm)	% *CIS*
1.21	0	0	8.8687	0
1.21	0.198	0.04	8.8726	0.386
1.21	0.387	0.08	8.8787	0.990
1.21	0.570	0.12	8.8827	1.39
1.21	0.916	0.19	8.8921	2.32
1.21	1.24	0.26	8.8987	2.97
1.21	1.68	0.35	8.9090	3.99
1.21	2.32	0.48	8.9231	5.39
1.21	2.88	0.60	8.9351	6.58
1.21	3.78	0.78	8.9529	8.34
1.21	4.48	0.93	8.9660	9.64
1.21	4.77	0.99	8.9722	10.2
1.21	10.1	2.09	9.0608	19.0
1.21	14.7	3.0	9.1536	28.2
1.21	29.8	6.2	9.3168	44.4
1.21	39.4	8.2	9.3944	52.1
1.21	51.1	10.6	9.4807	60.6
1.21	149	30.9	9.8785	100

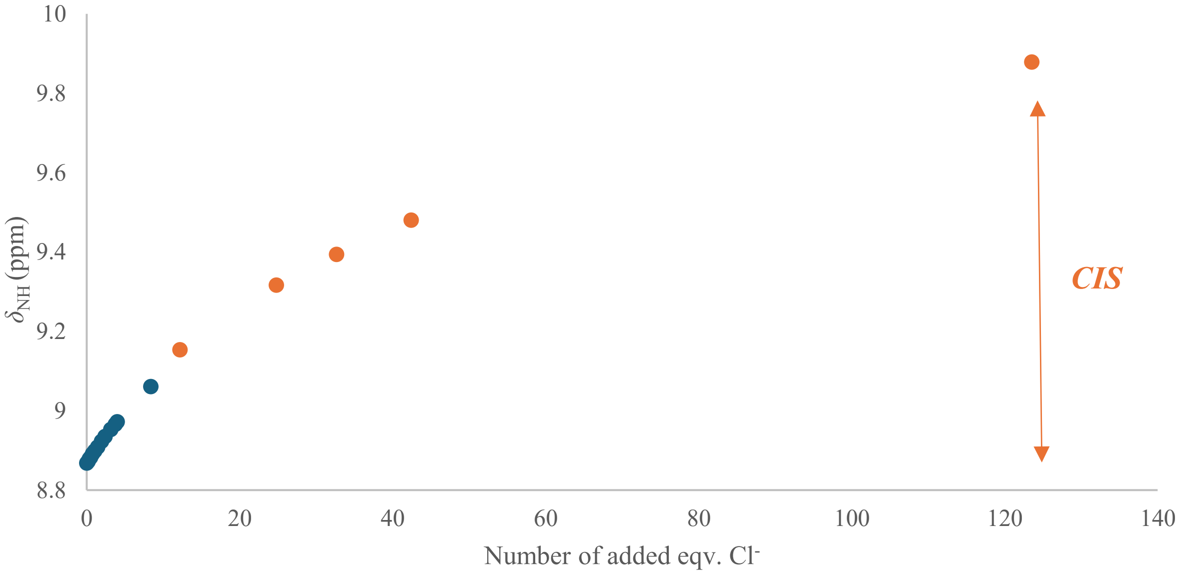

Fig. S23a: Binding isotherm obtained during ^{1}H NMR titration of **6** (1.21 mM, DMSO-d_6) with solution of guest (containing **6**:1.21 mM; TBACl: 149 mM, DMSO-d_6).

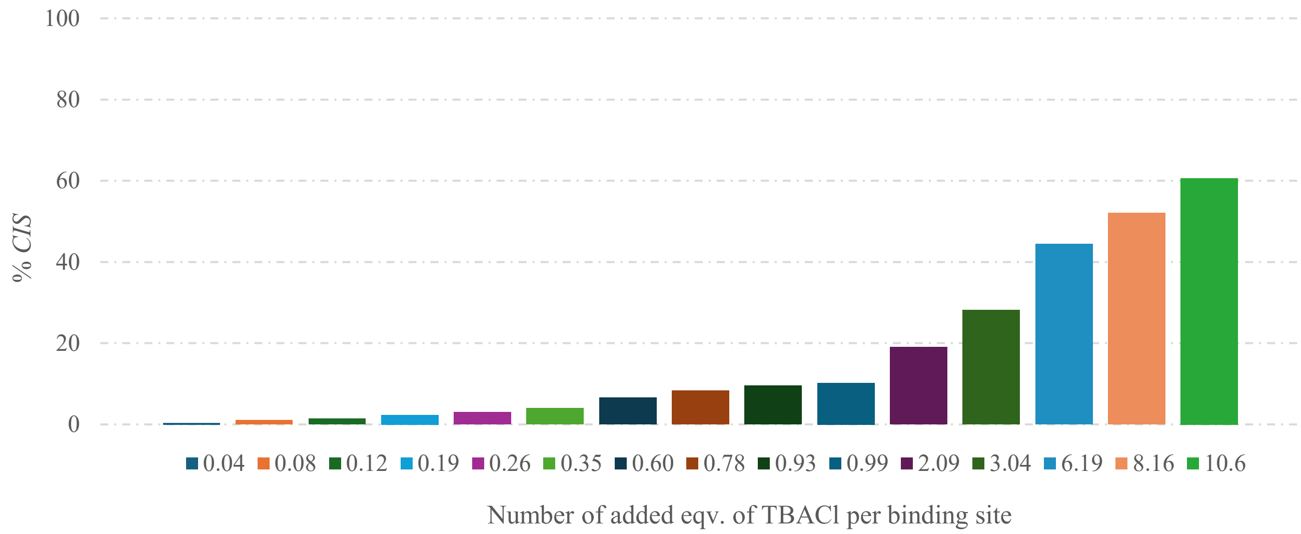

Fig. S23b: The evaluation of binding efficiency of receptor **6** with TBACl. The complexation ability is expressed by % of *CIS* (y-axes), which depends on number of added equiv. of TBACl per binding unit.

c (**7**) mM	*c* (BzO^-) mM	Equiv. per binding site	δ_{NH} (ppm)	% *CIS*
2.43	0.00	0	8.9829	0
2.43	0.19	0.04	8.9915	1.70
2.43	0.38	0.08	9.0207	3.42
2.43	0.55	0.11	9.0667	6.19
2.43	0.89	0.18	9.1624	11.9
2.43	1.20	0.25	9.2563	17.5
2.43	1.63	0.34	9.3798	24.9
2.43	2.26	0.46	9.5446	34.8
2.43	2.80	0.58	9.6643	42.0
2.43	3.67	0.75	9.8419	52.7
2.43	4.35	0.89	9.9487	59.1
2.43	4.64	0.95	9.9902	61.5
2.43	5.71	1.17	10.1212	69.1
2.43	6.30	1.29	10.1901	73.2
2.43	9.79	2.01	10.6311	100

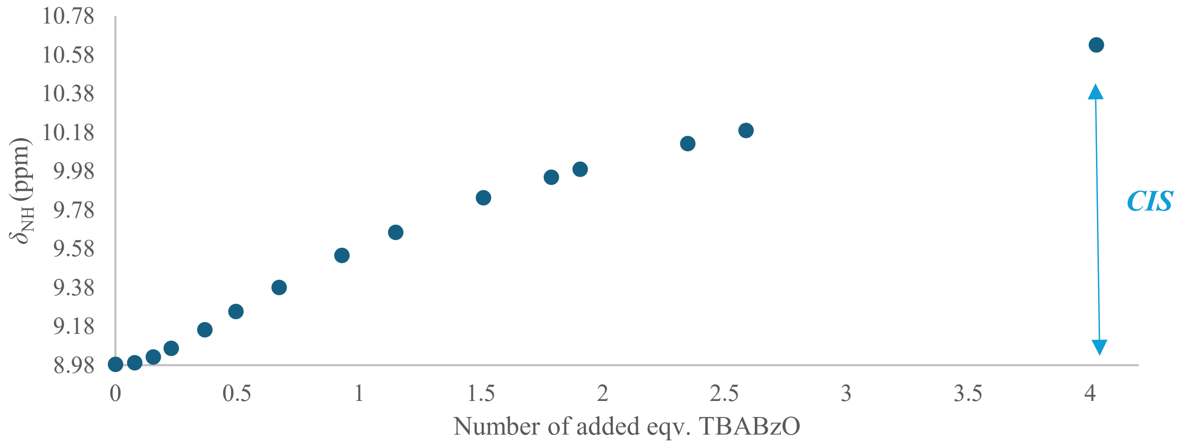

Fig. S24a: Binding isotherm obtained during ^{1}H NMR titration of **7** (2.4 mM, DMSO-d_6) with solution of guest (containing **7**: 2.4 mM; TBABzO: 9.79 mM, DMSO-d_6).

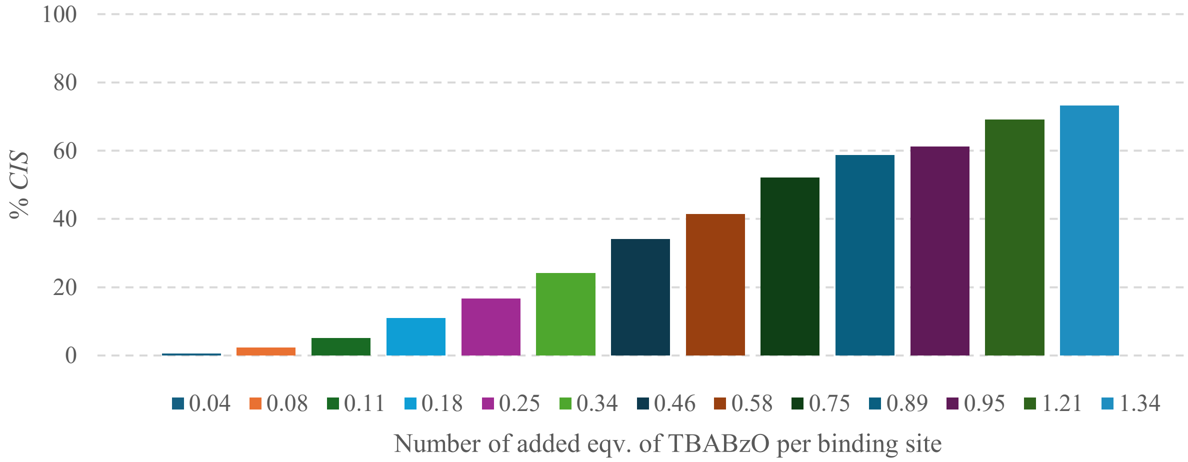

Fig. S24b: The evaluation of binding efficiency of receptor **7** with TBABzO. The complexation ability is expressed by % of *CIS* (y-axes), which depends on number of added equiv. of TBABzO per binding unit.

Link: http://app.supramolecular.org/bindfit/view/d3e2684a-2c4e-4ea0-ace5-3763e2bdbbe5

3. Spectral records

3.1 Platforms

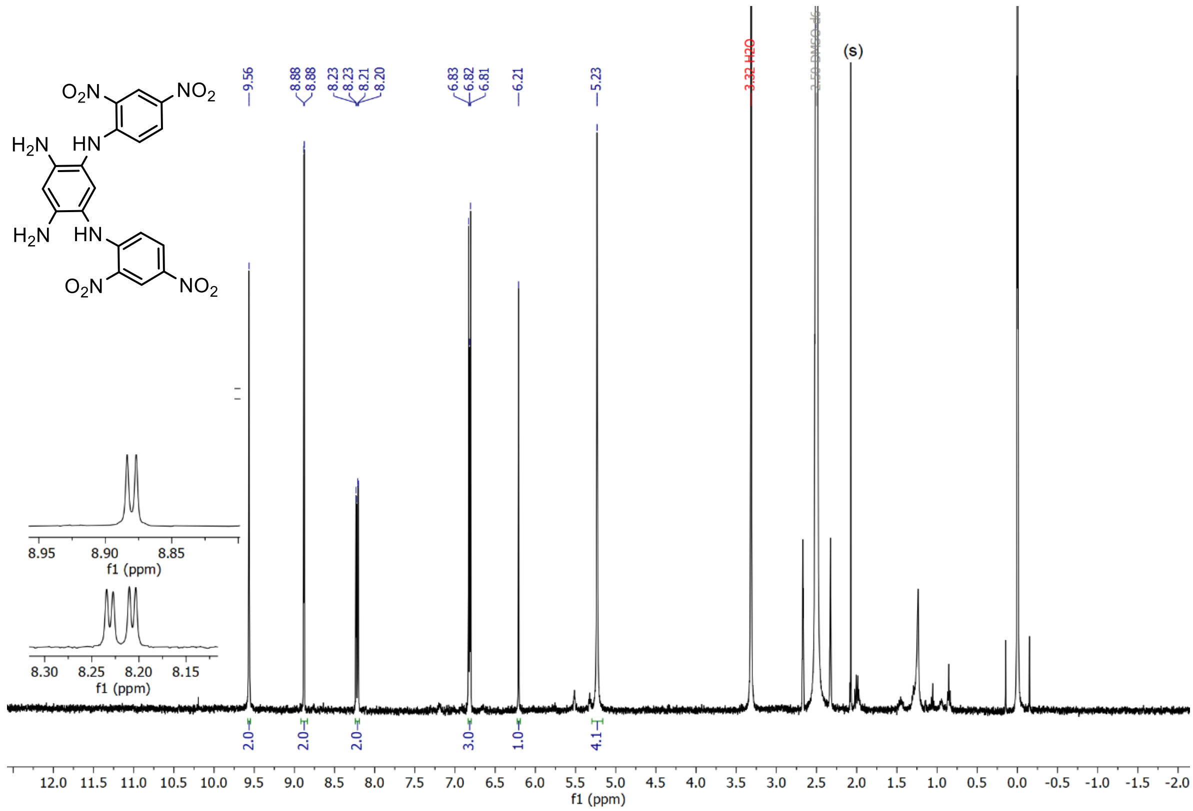

Fig. S25: ^{1}H NMR of compound **3a** (DMSO-d_6, 400 MHz).

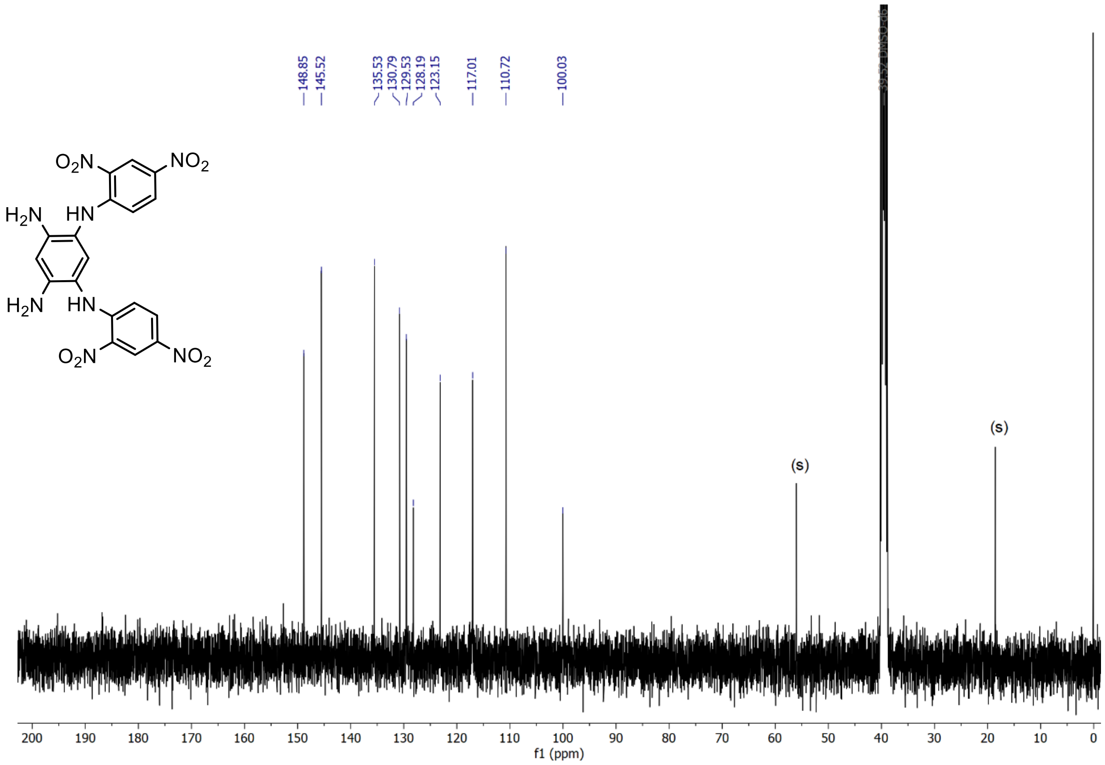

Fig. S26: $^{13}C\{^{1}H\}$ NMR of compound **3a** (DMSO-d_6, 100 MHz).

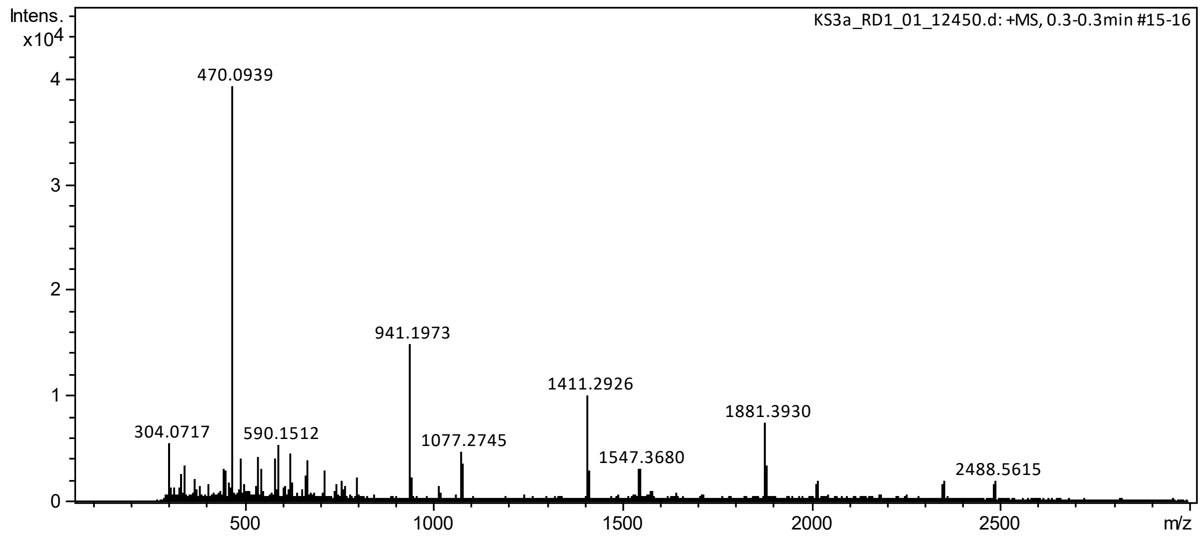

Fig. S27: HRMS (ESI+) of compound **3a** $[C_{18}H_{14}N_8O_8]^+$ calcld. 470.0929; found 470.0939 $[M]^+$.

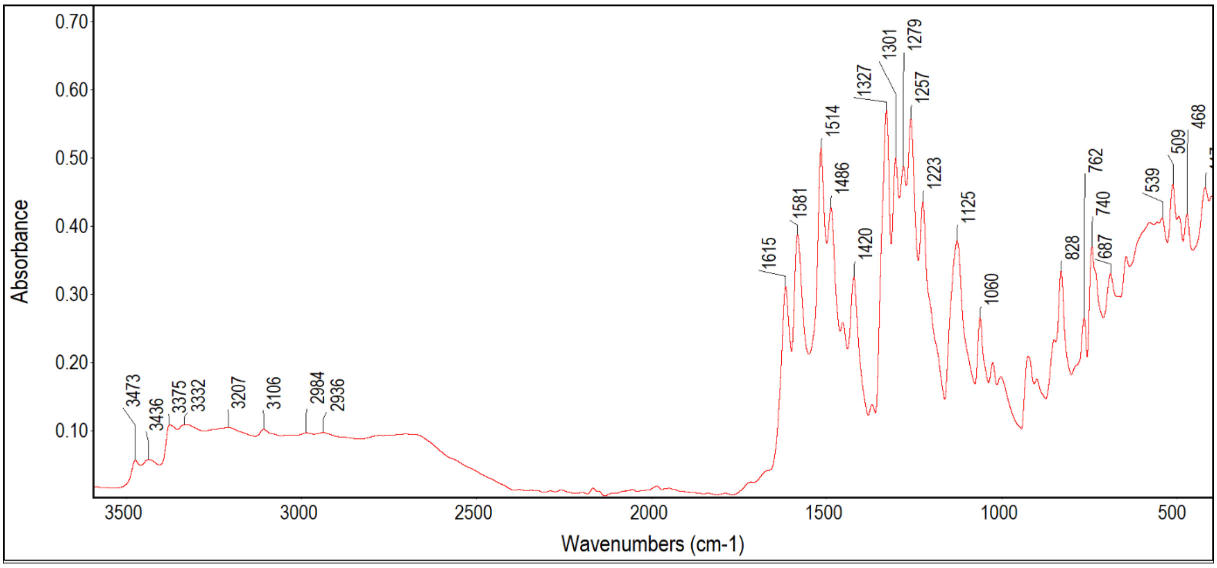

Fig. S28: IR (ATR) spectrum of compound **3a**.

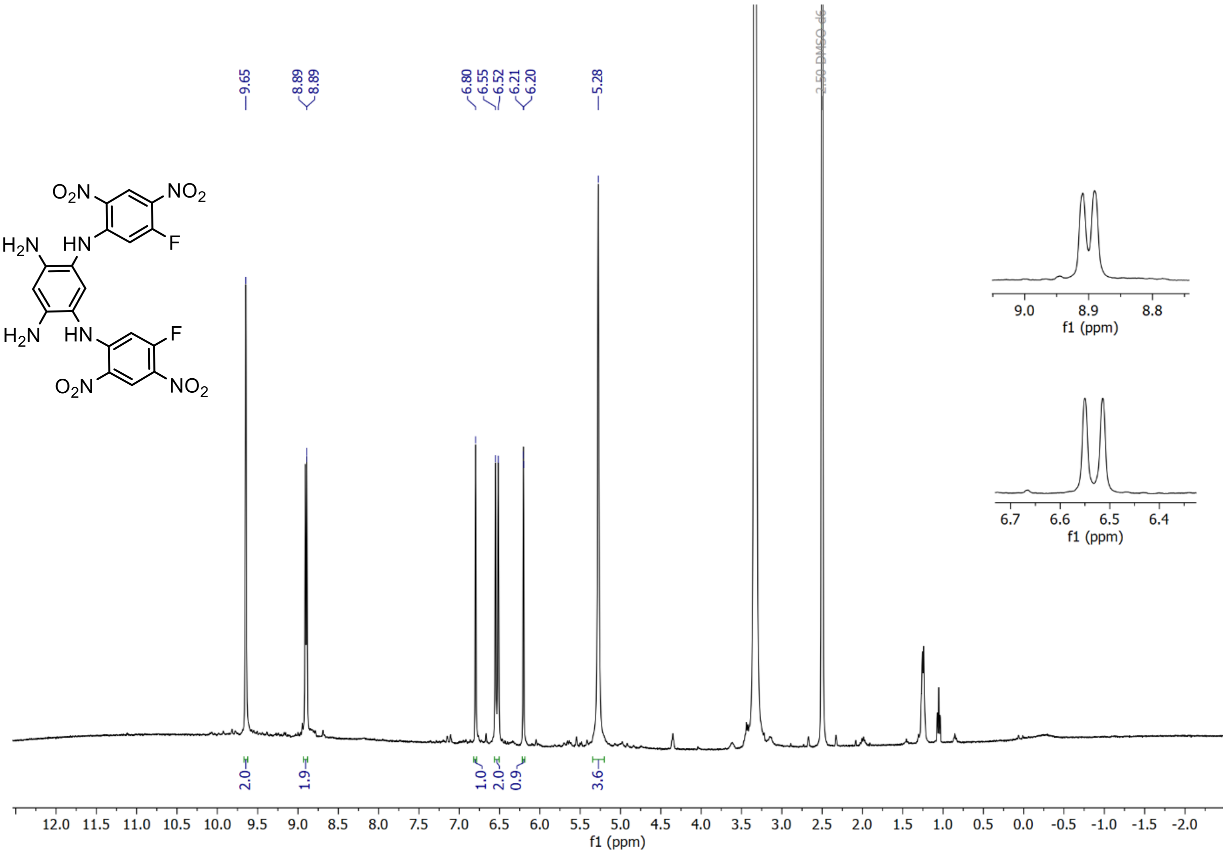

Fig. S29: ^{1}H NMR of compound **3b** (DMSO-d_6, 400 MHz).

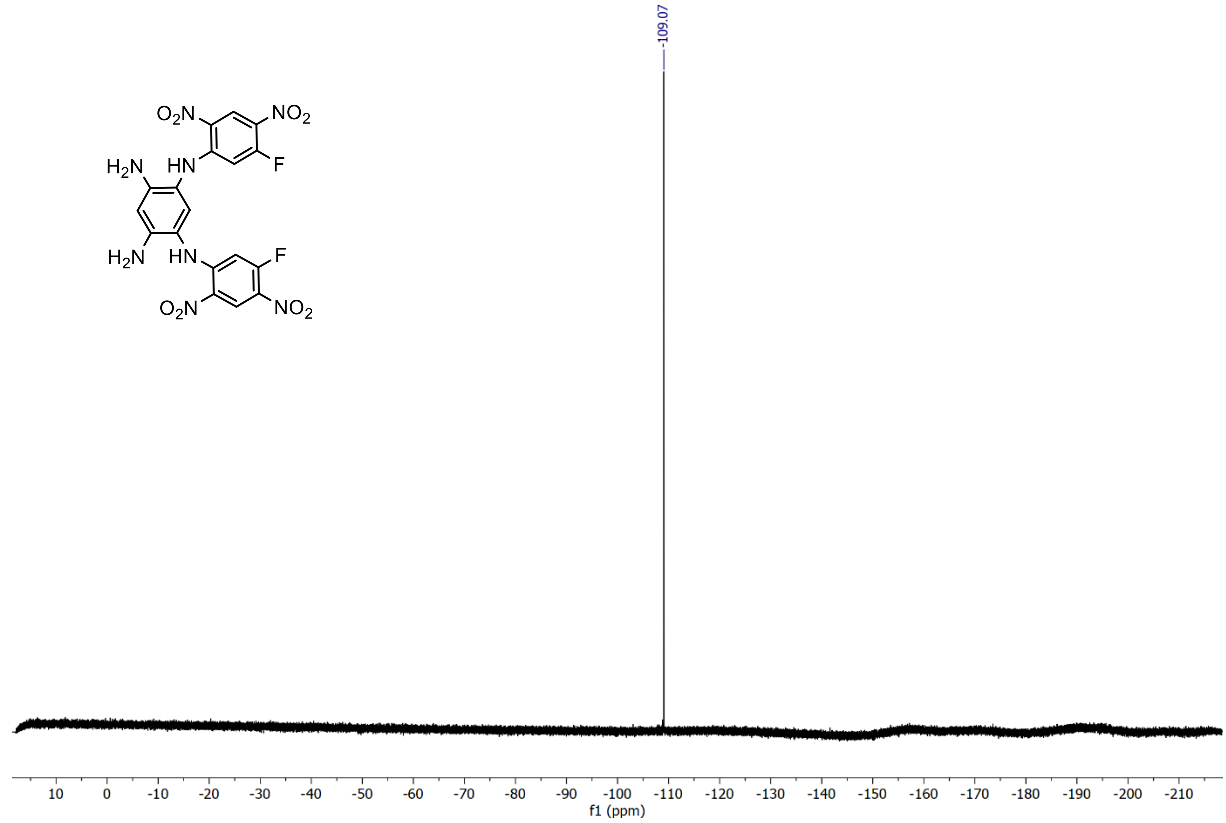

Fig. S30: ^{19}F NMR of compound **3b** (DMSO-d_6, 376 MHz).

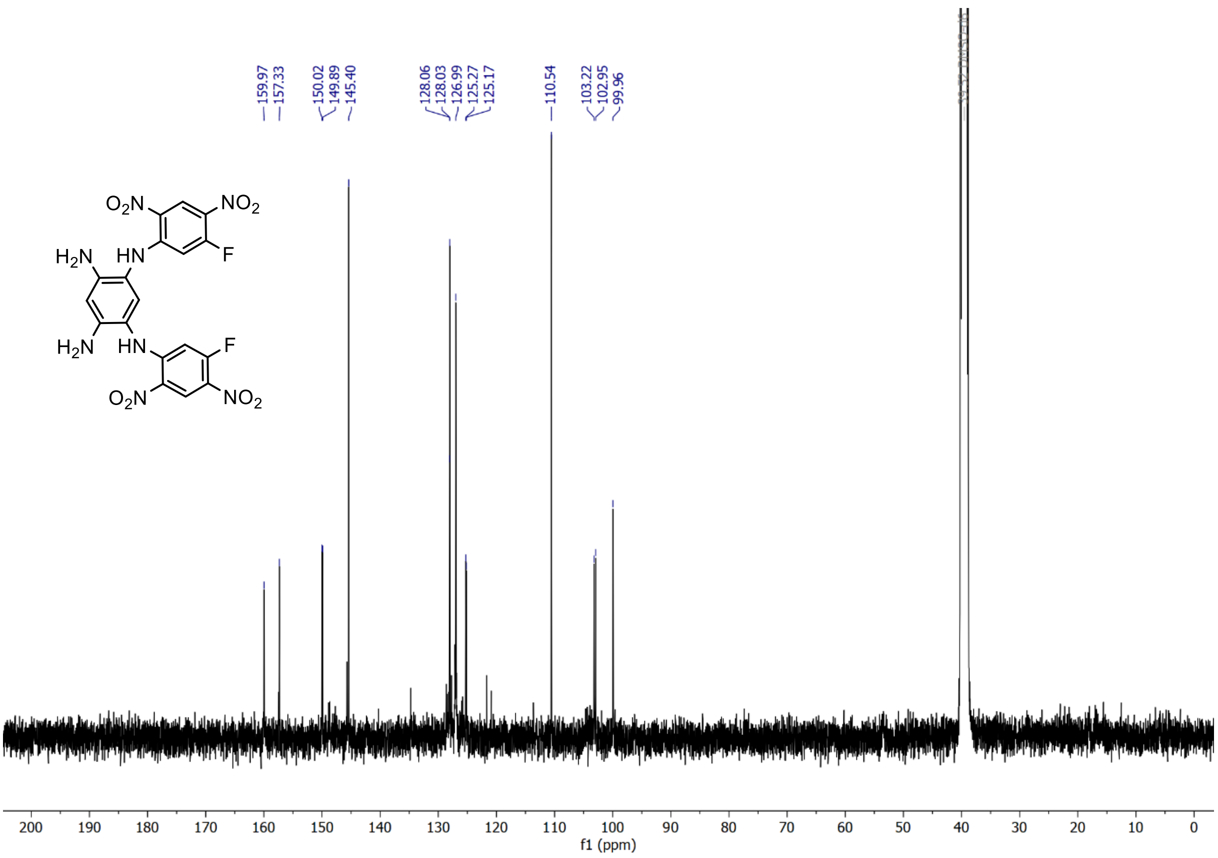

Fig. S31: $^{13}C\{^1H\}$ NMR of compound **3b** (DMSO-d_6, 100 MHz).

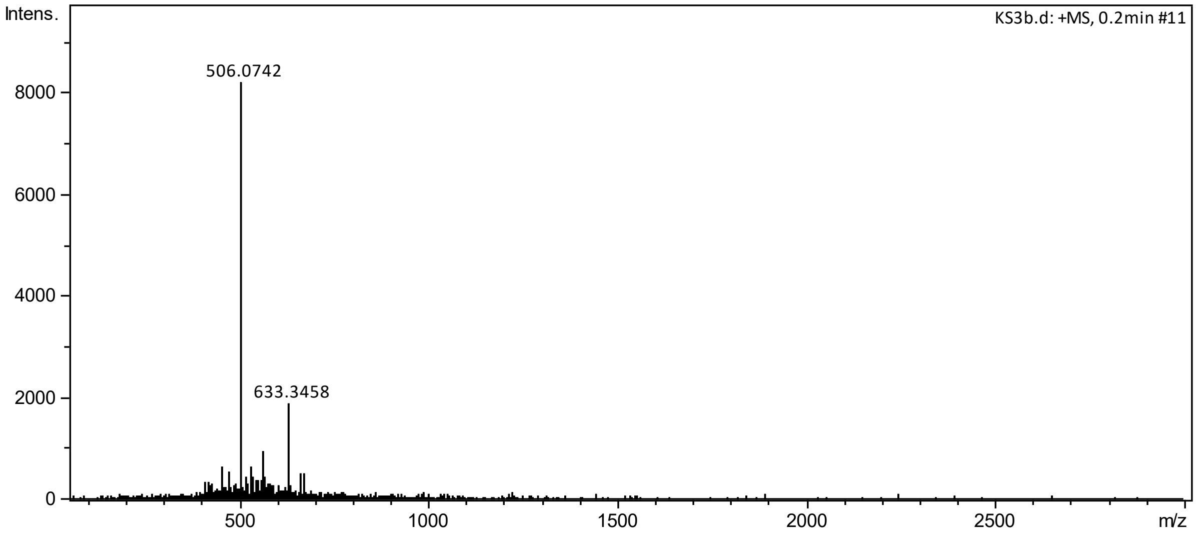

Fig. S32: HRMS (APCI+) of compound **3b** $[C_{18}H_{12}F_2N_8O_8]^+$ calcld. 506.0741; found 506.0742 $[M]^+$.

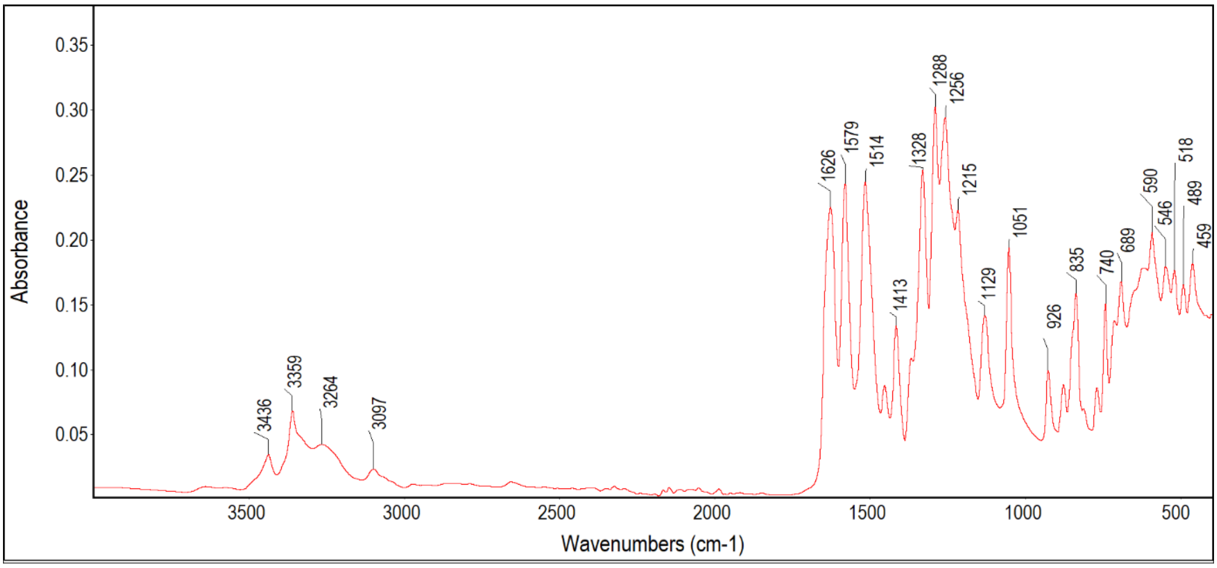

Fig. S33: IR (ATR) spectrum of compound **3b**.

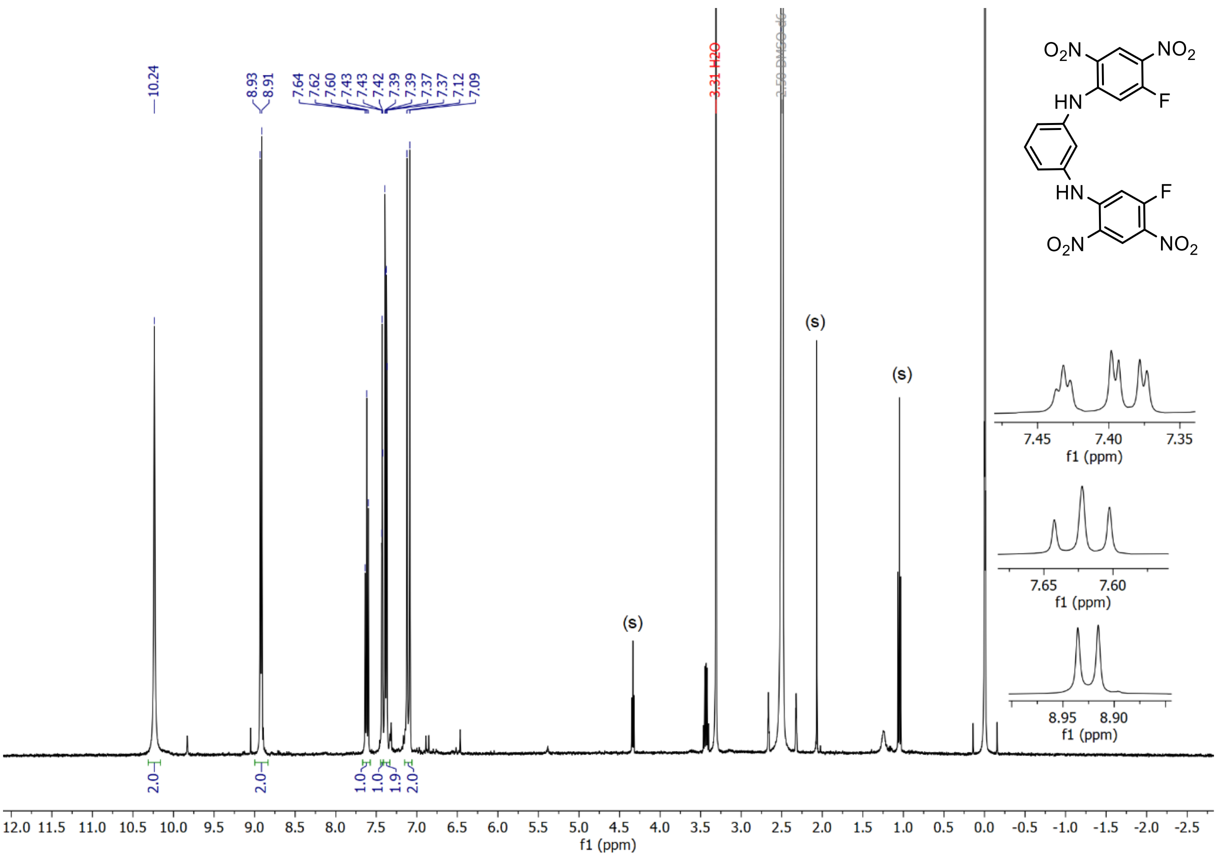

Fig. S34: ^{1}H NMR of compound **3c** (DMSO-d_6, 400 MHz).

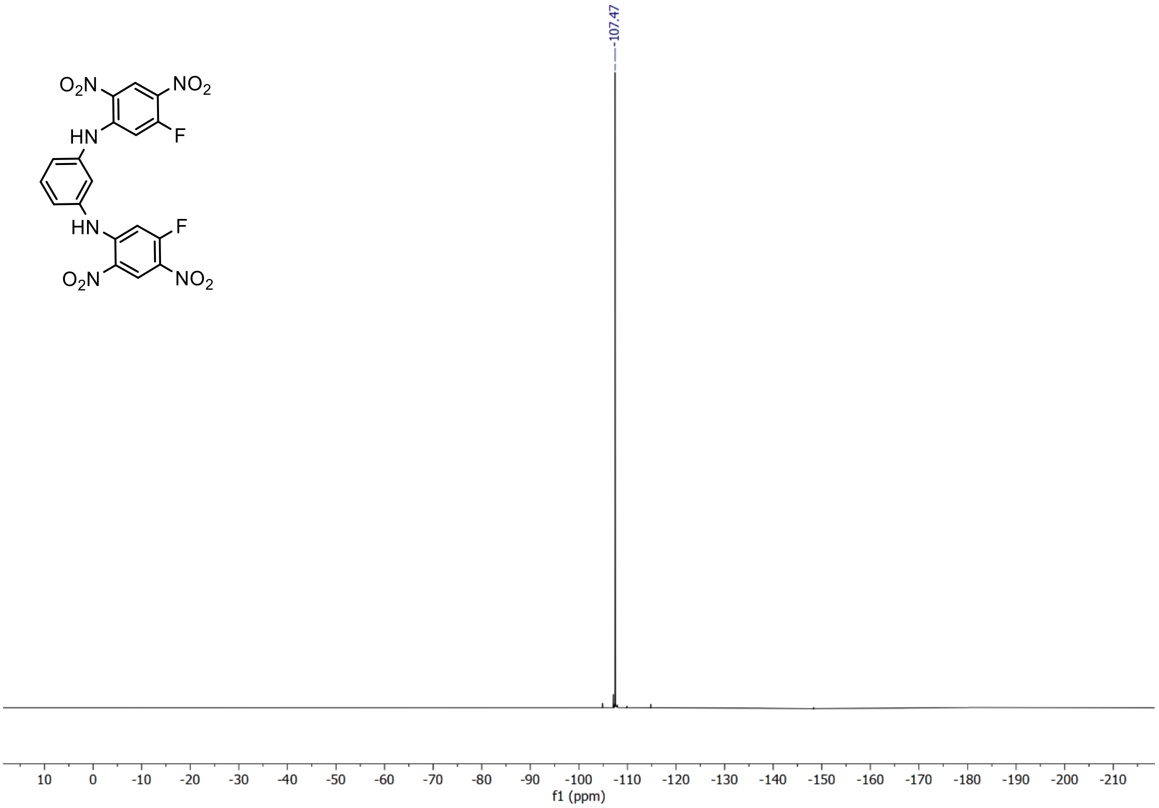

Fig. S35: ^{19}F NMR of compound **3c** (DMSO-d_6, 376 MHz).

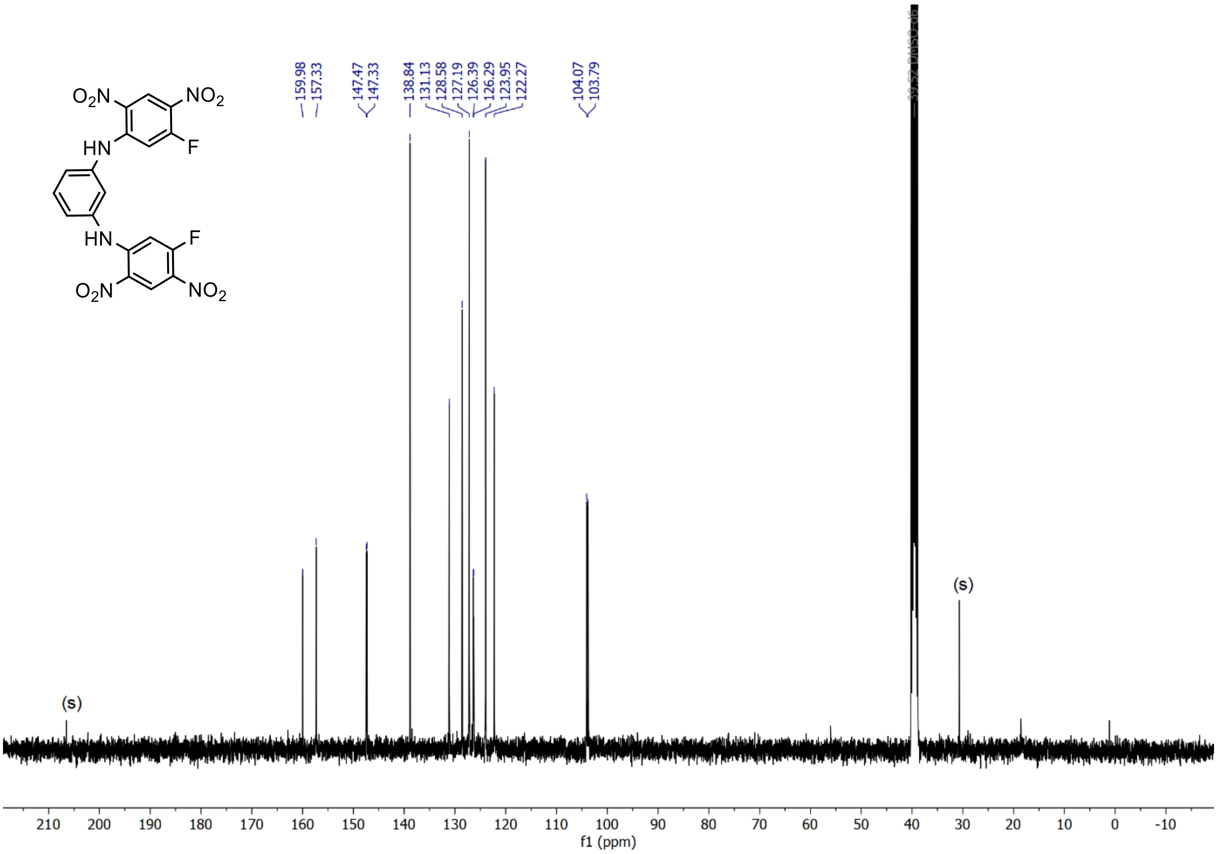

Fig. S36: $^{13}C\{^{1}H\}$ NMR of compound **3c** (DMSO-d_6, 100 MHz).

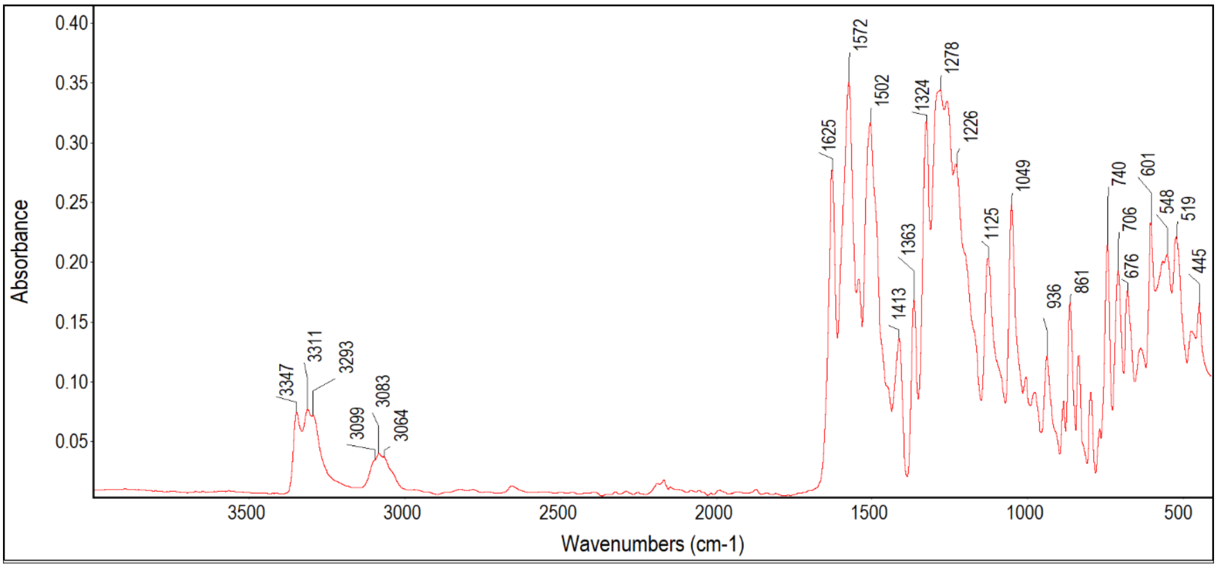

Fig. S37: IR (ATR) spectrum of compound **3c**.

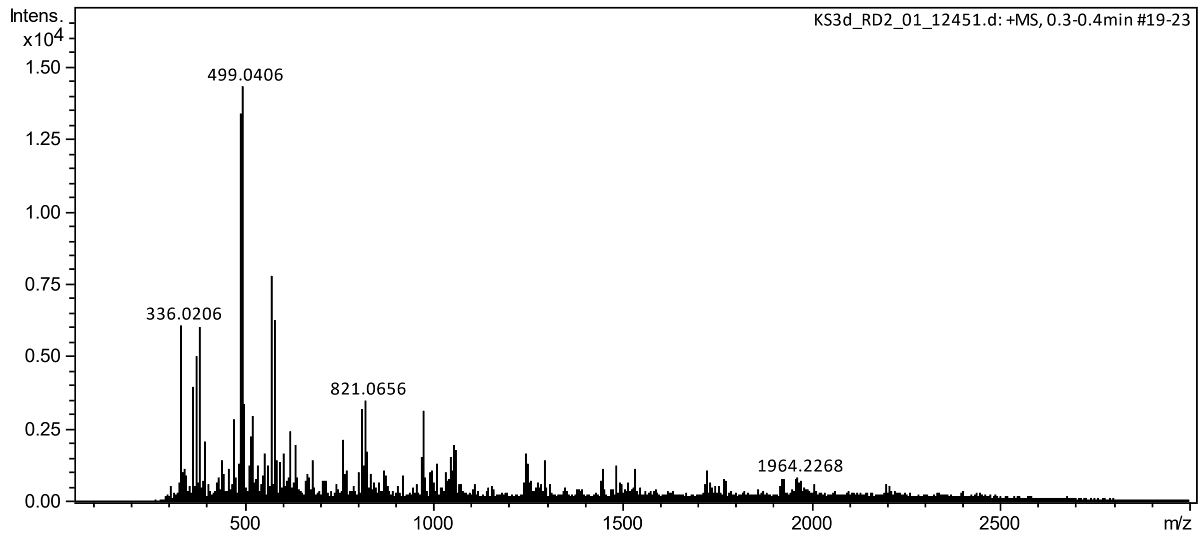

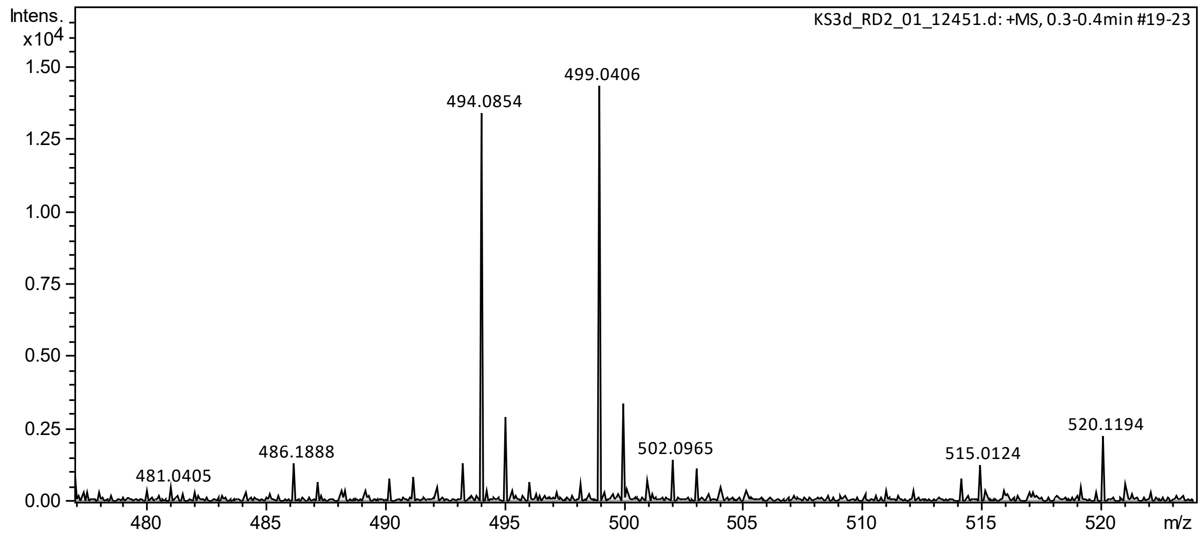

Fig. S38: HRMS (ESI+) of compound **3c** $[C_{18}H_{10}F_2N_6O_8+Na]^+$ calcld. 499.0420; found 499.0406 $[M+Na]^+$.

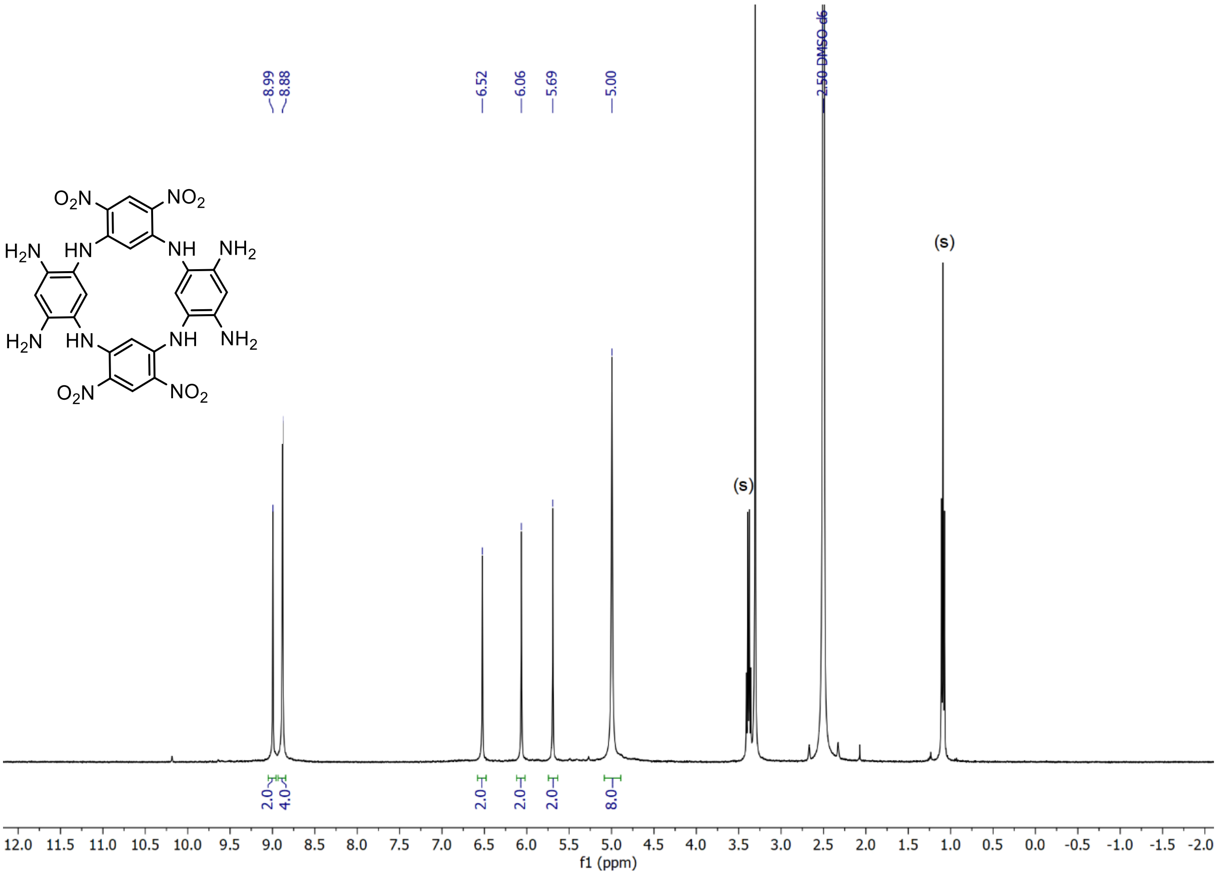

Fig. S39: ^{1}H NMR of compound **4a** (DMSO-d_6, 400 MHz).

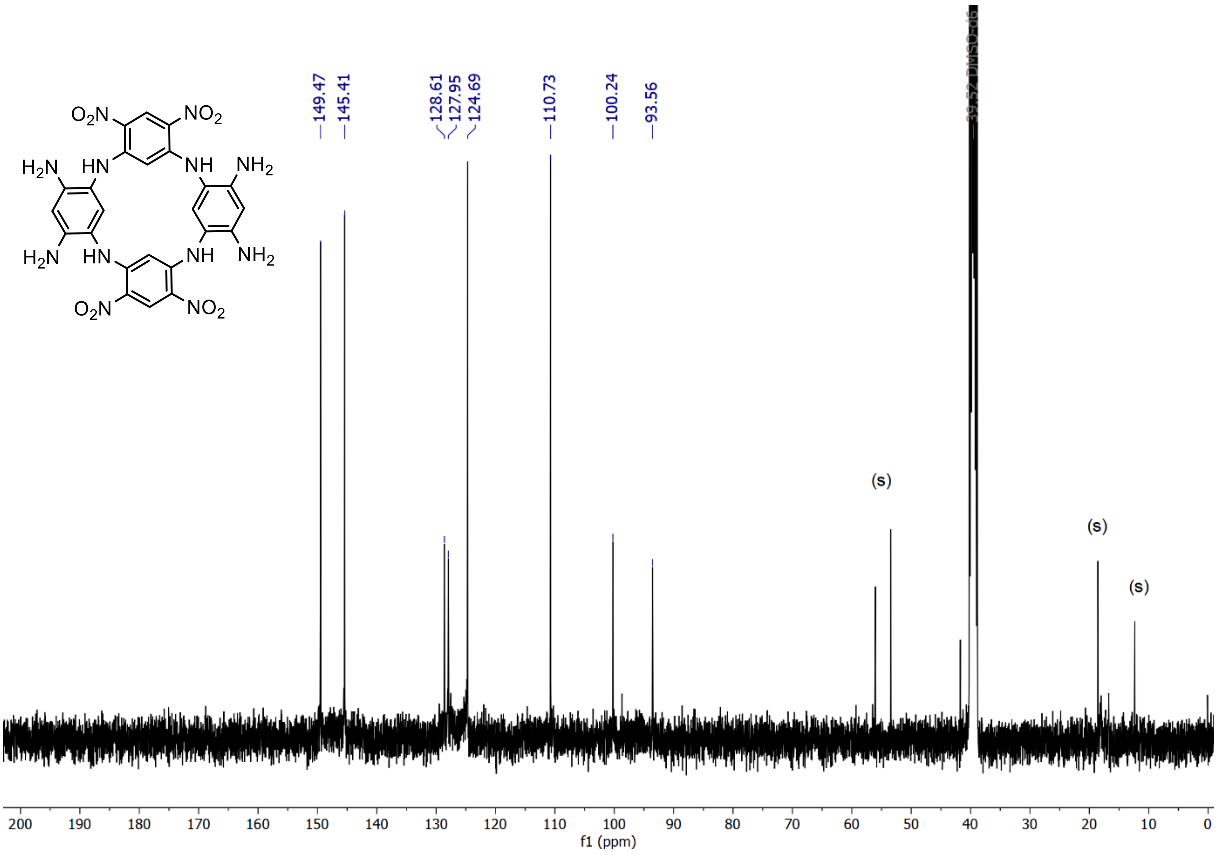

Fig. S40: $^{13}C\{^{1}H\}$ NMR of compound **4a** (DMSO-d_6, 100 MHz).

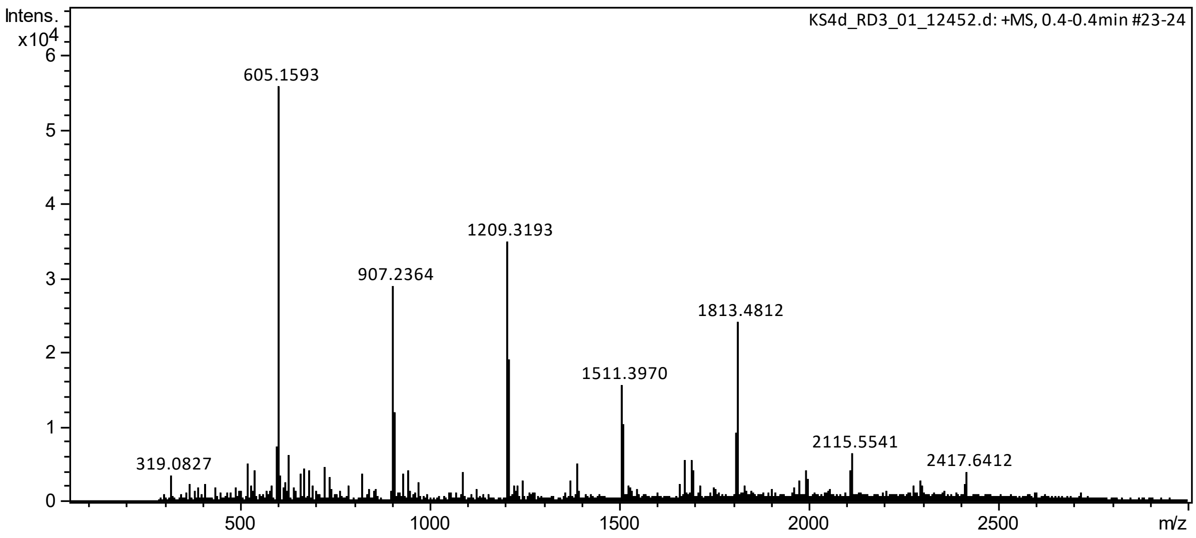

Fig. S41: HRMS (ESI+) of compound **4a** $[C_{24}H_{20}N_{12}O_8+H]^+$ calcld. 605.1600; found 605.1593 $[M+H]^+$.

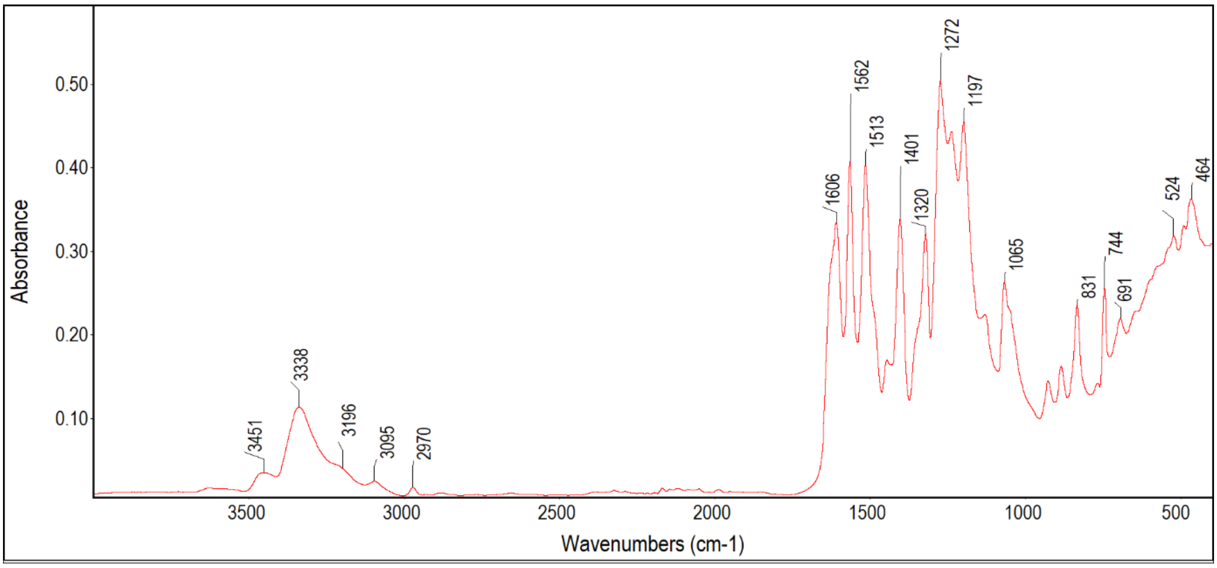

Fig. S42: IR (ATR) spectrum of compound **4a**.

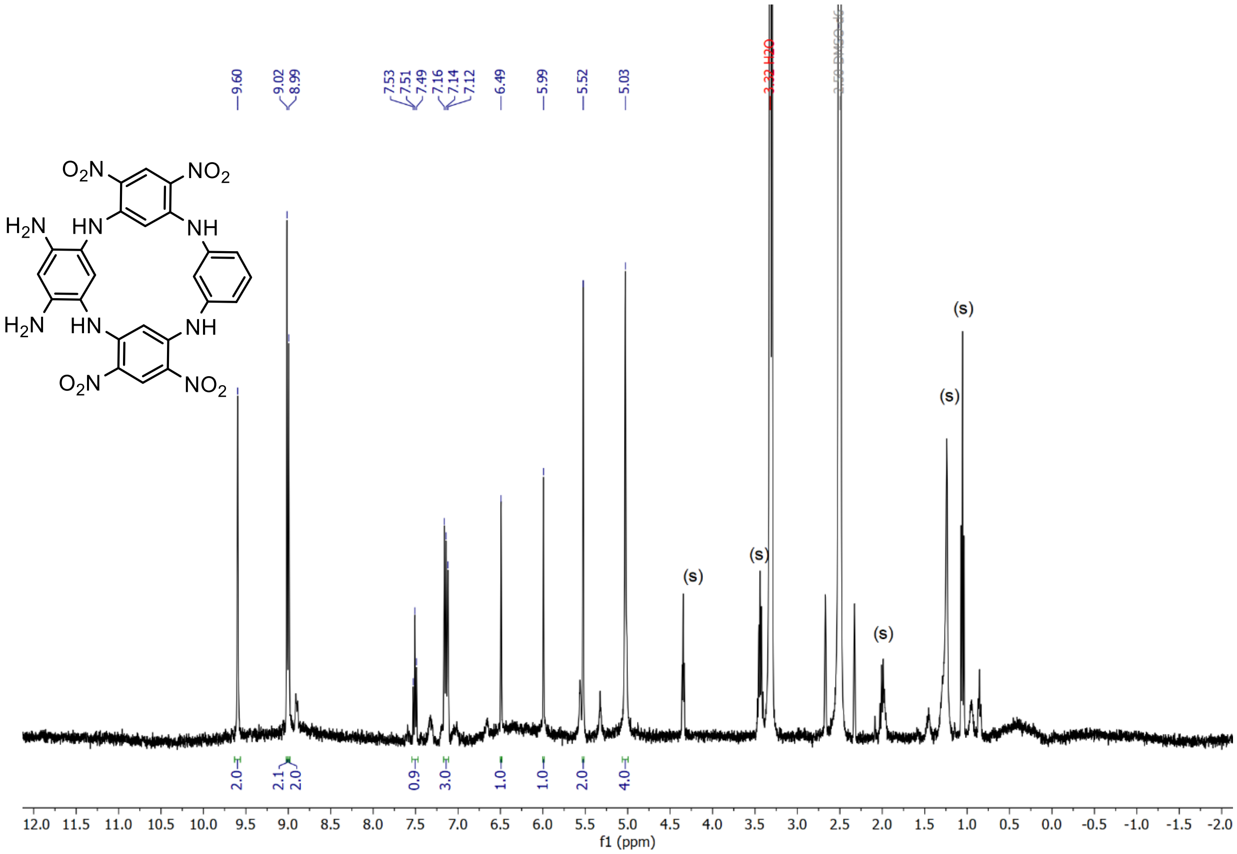

Fig. S43: ^{1}H NMR of compound **4b** (DMSO-d_6, 400 MHz).

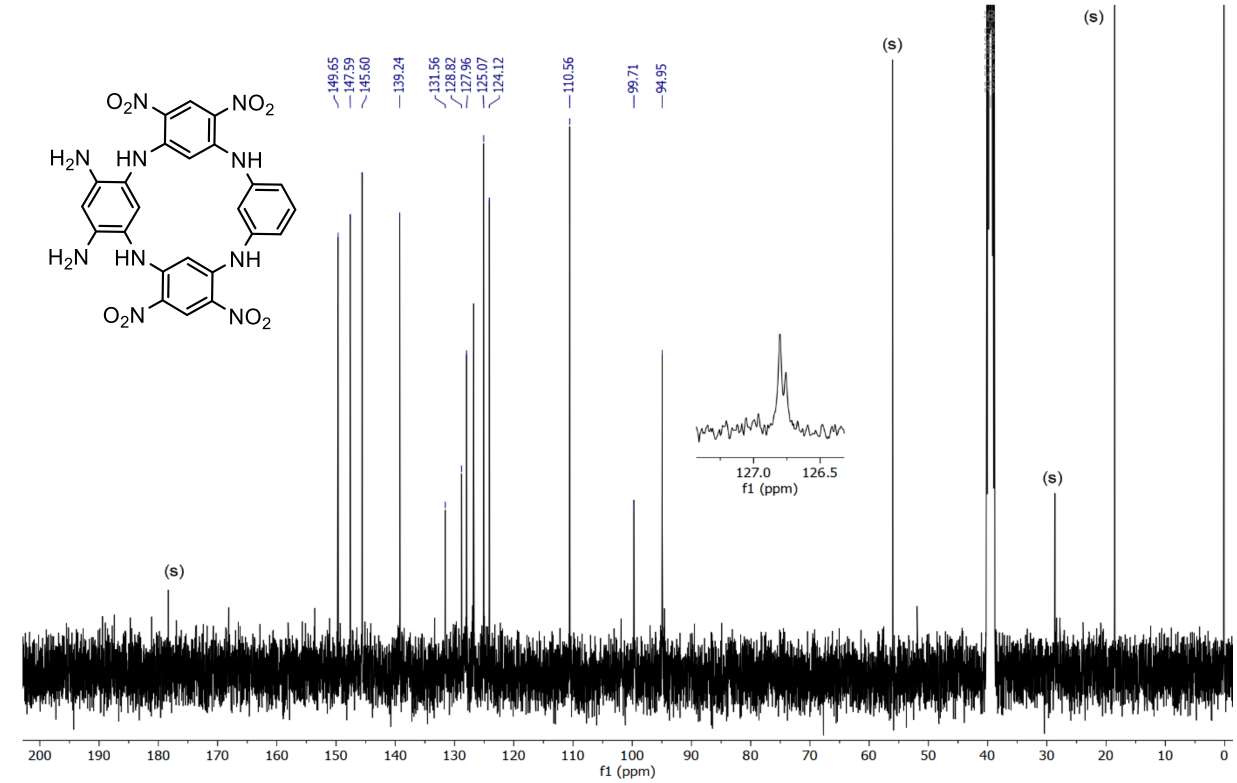

Fig. S44: ^{13}C{^{1}H} NMR of compound **4b** (DMSO-d_6, 100 MHz).

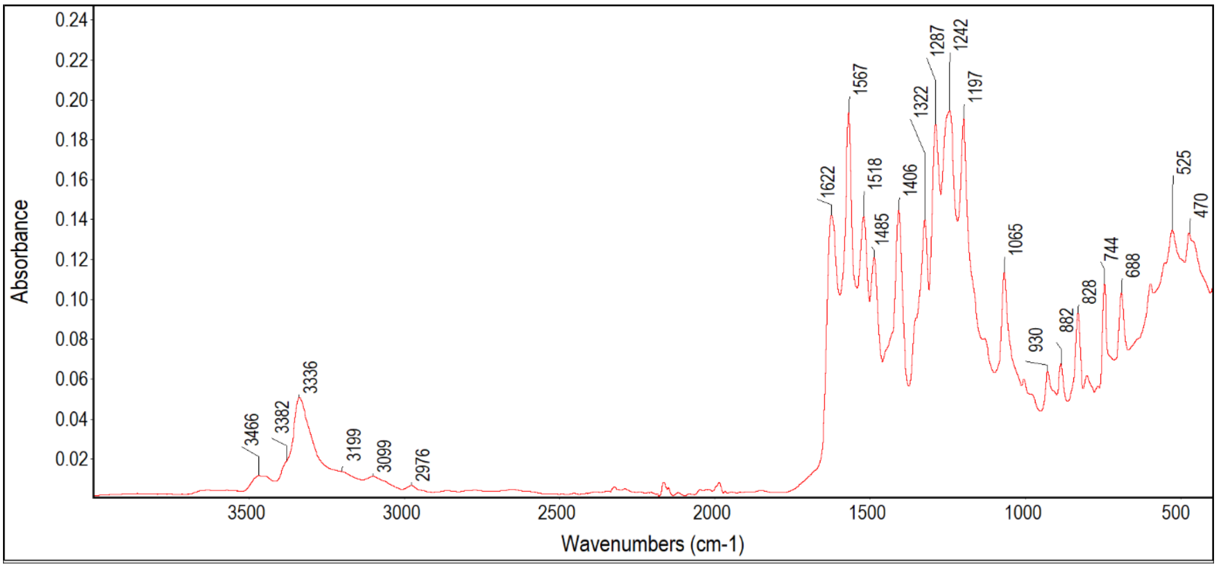

Fig. S45: IR (ATR) spectrum of compound **4b**.

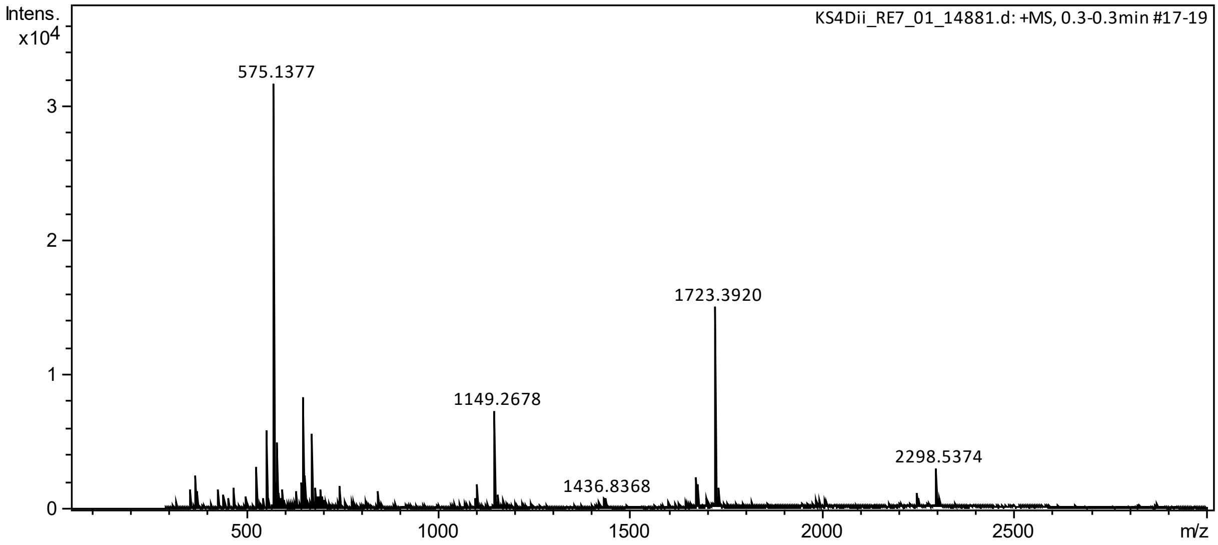

Fig. S46: HRMS (ESI+) of compound **4b** $[C_{24}H_{18}N_{10}O_8+H]^+$ calcld. 575.1382; found 575.1377 $[M+H]^+$.

3.2 Ureido-derivatives

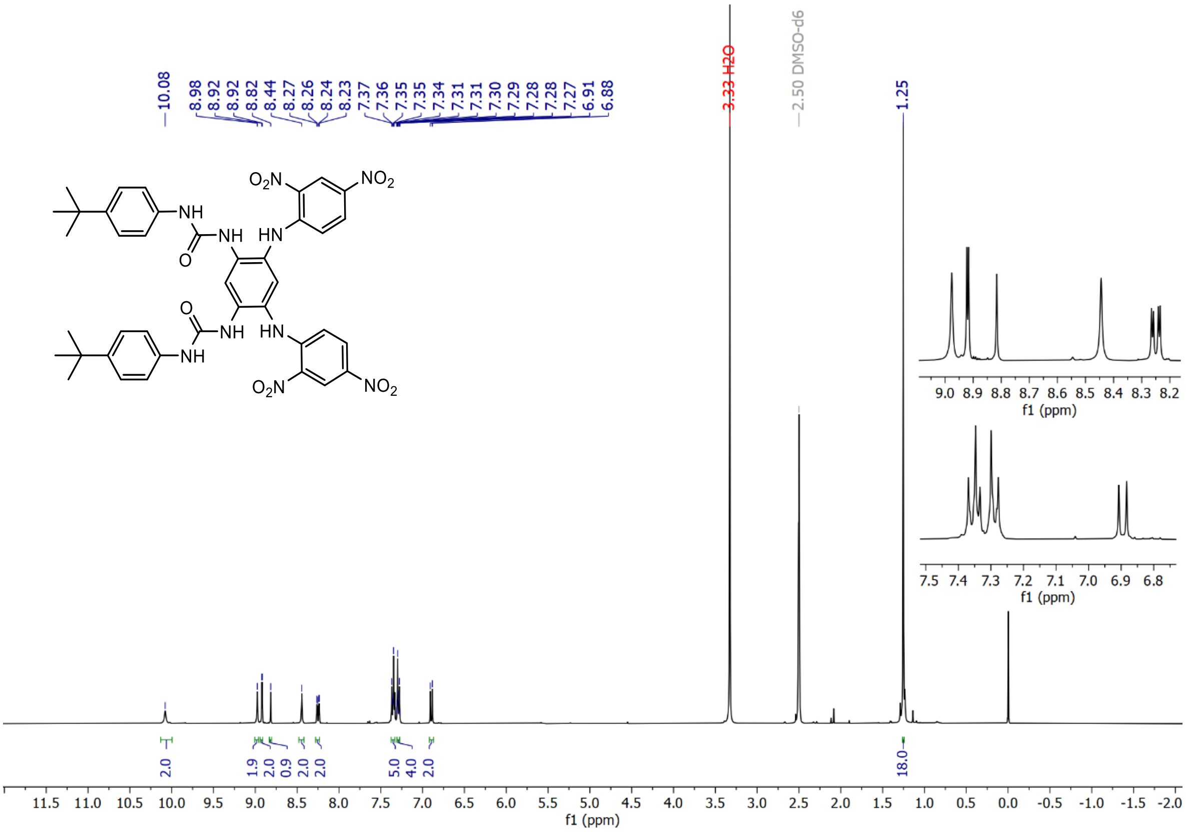

Fig. S47: ^{1}H NMR of compound **5a** (DMSO-d_6, 400 MHz).

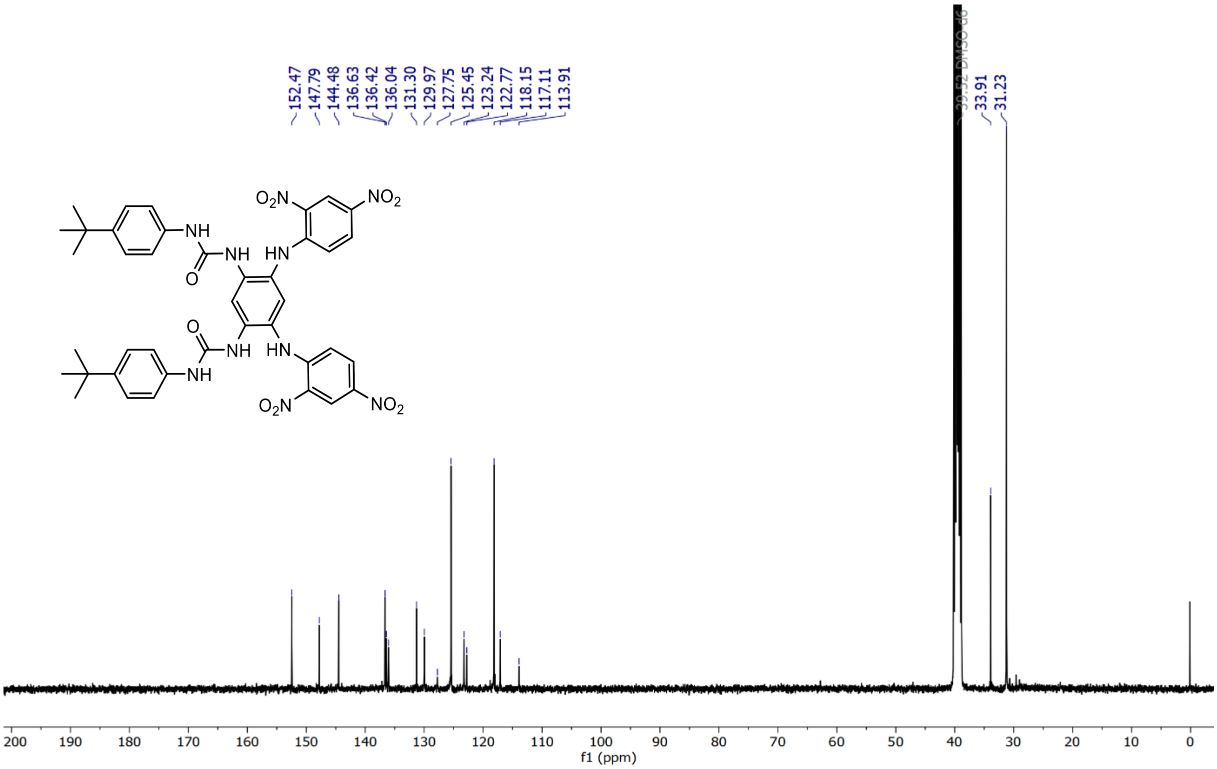

Fig. S48: ^{13}C{^{1}H} NMR of compound **5a** (DMSO-d_6, 100 MHz).

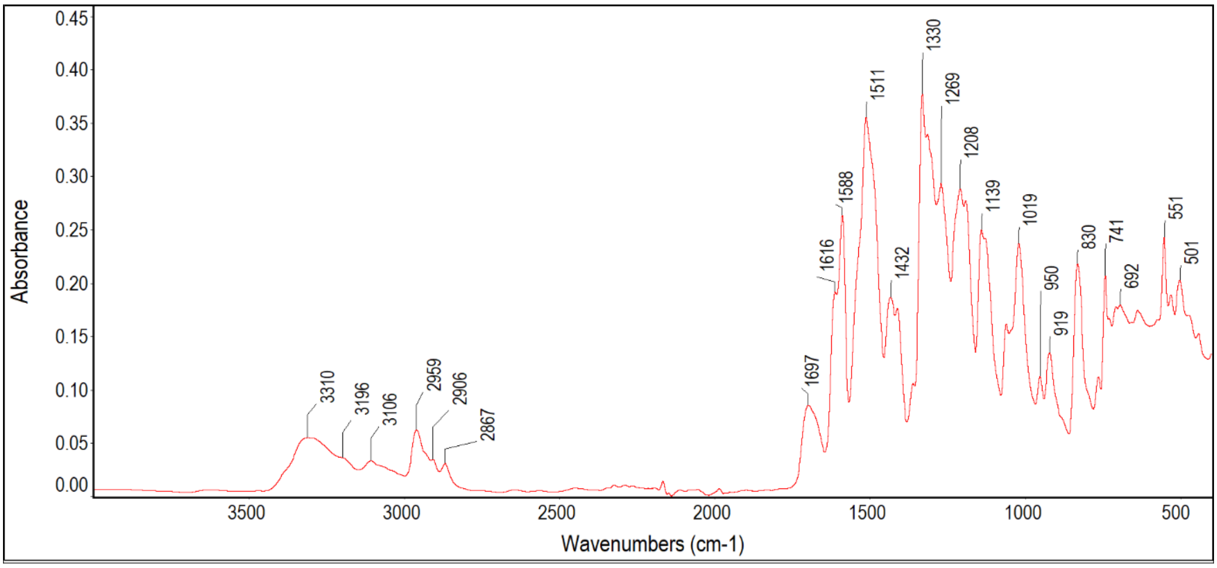

Fig. S49: IR (ATR) spectrum of compound **5a**.

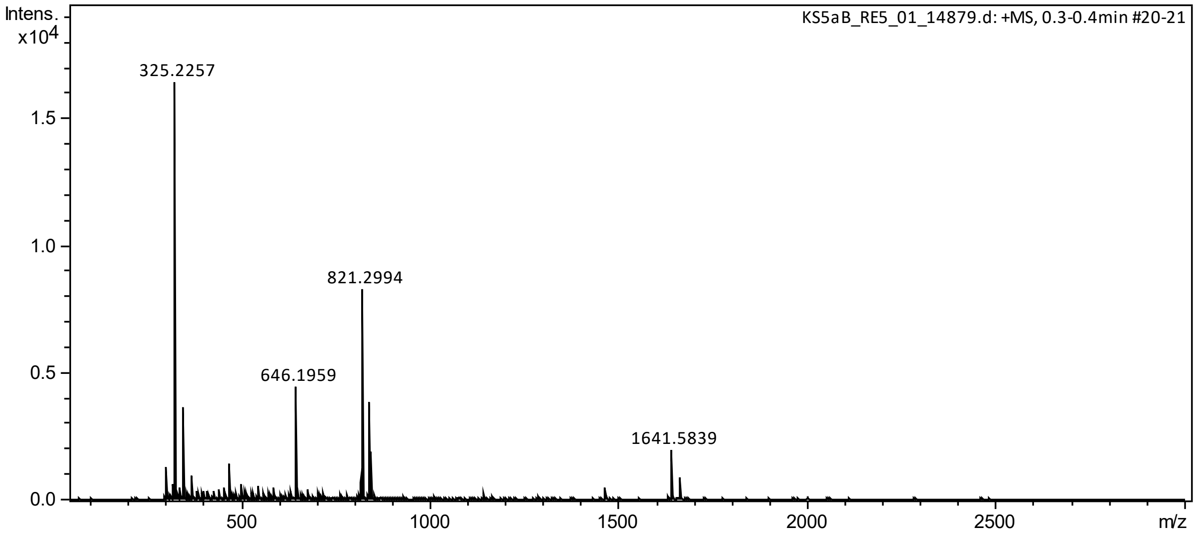

Fig. S50: HRMS (ESI+) of compound **5a** calcld. $[C_{40}H_{40}N_{10}O_{10}+H]^+$ 821.3002; found 821.2994 $[M+H]^+$; calcld. $[C_{80}H_{80}N_{20}O_{20}+H]^+$ 1641.5936; found 1641.5839 $[2M+H]^+$.

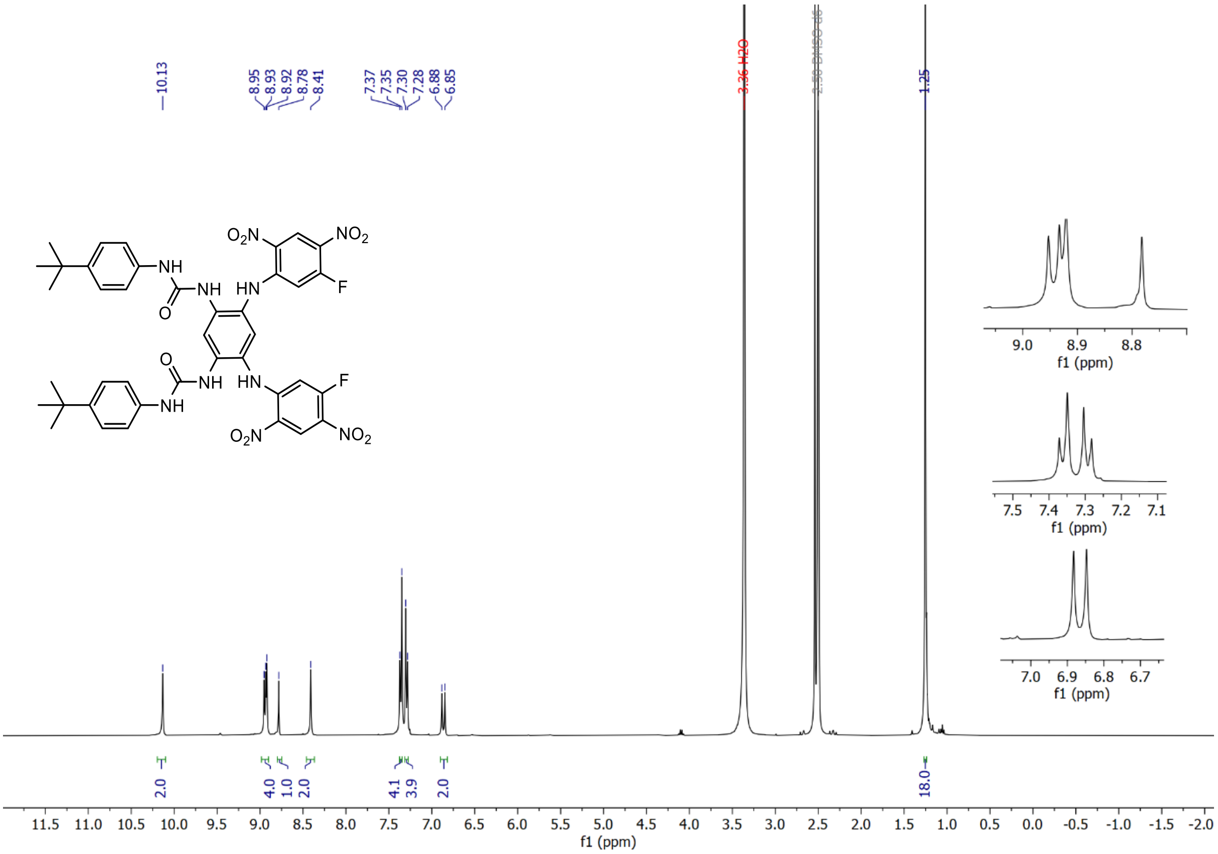

Fig. S51: ^{1}H NMR of compound **5b** (DMSO-d_6, 400 MHz).

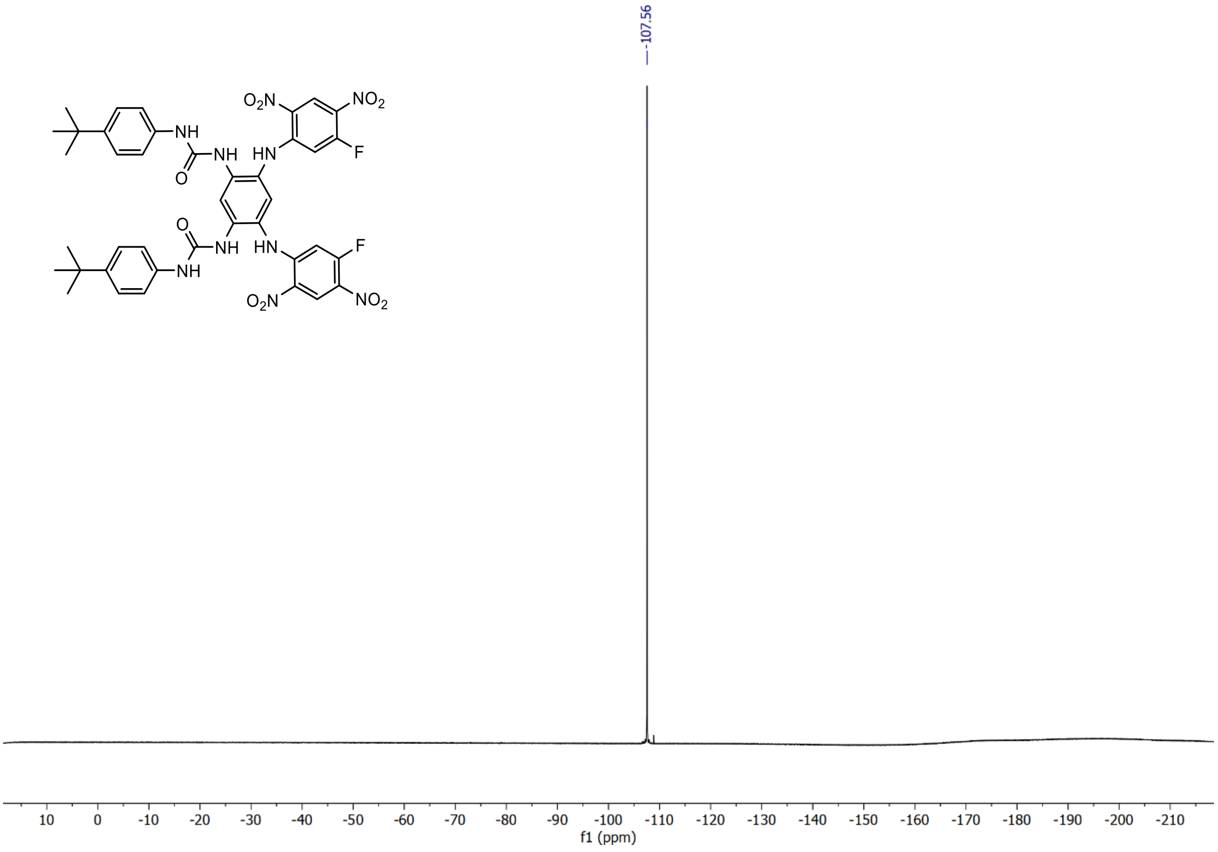

Fig. S52: ^{19}F NMR of compound **5b** (DMSO-d_6, 376 MHz).

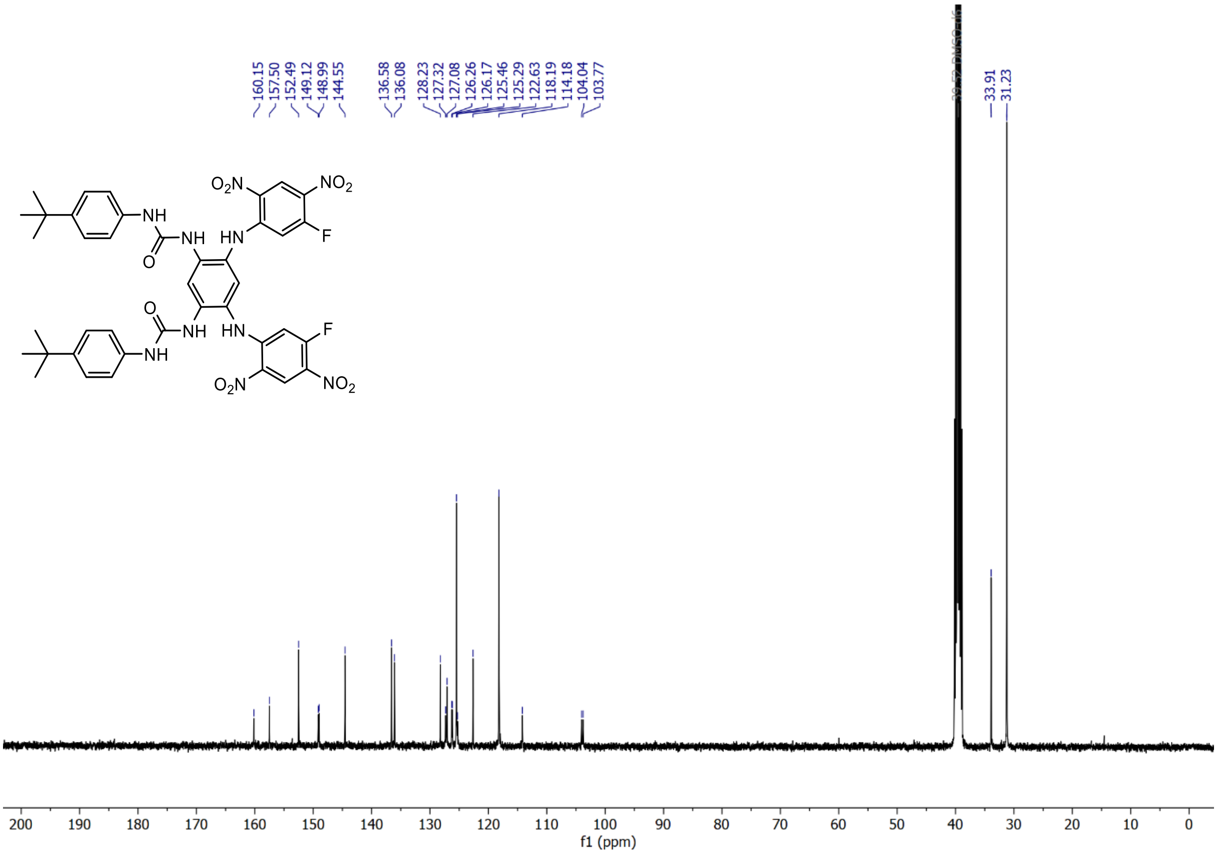

Fig. S53: $^{13}C\{^{1}H\}$ NMR of compound **5b** (DMSO-d_6, 100 MHz).

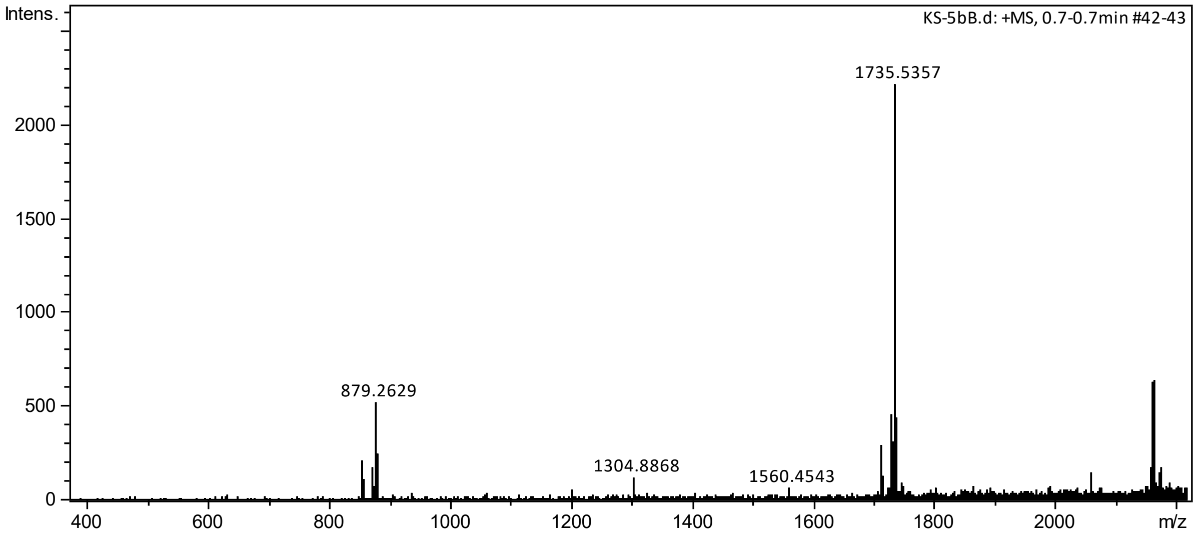

Fig. S54: HRMS (ESI+) of compound **5b** calcld. $[C_{40}H_{38}F_2N_{10}O_{10}+Na]^+$ 879.2632; found 879.2629 $[M+Na]^+$; calcld. $[C_{80}H_{76}F_4N_{20}O_{20}+Na]^+$ 1735.537; found 1735.5357 $[2M+Na]^+$.

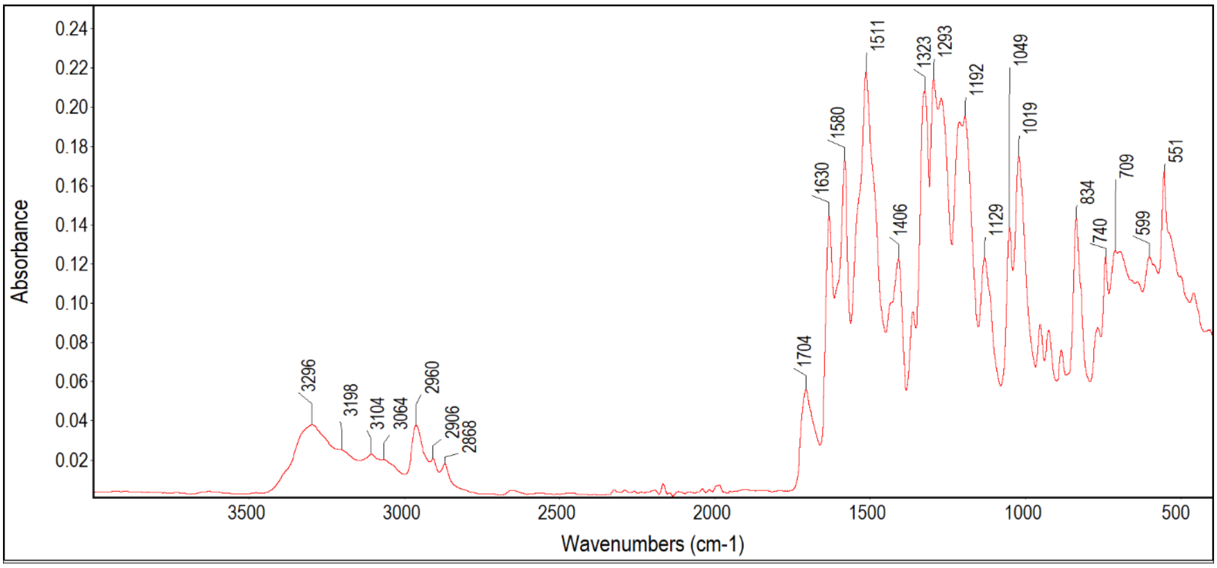

Fig. S55: IR (ATR) spectrum of compound **5b**.

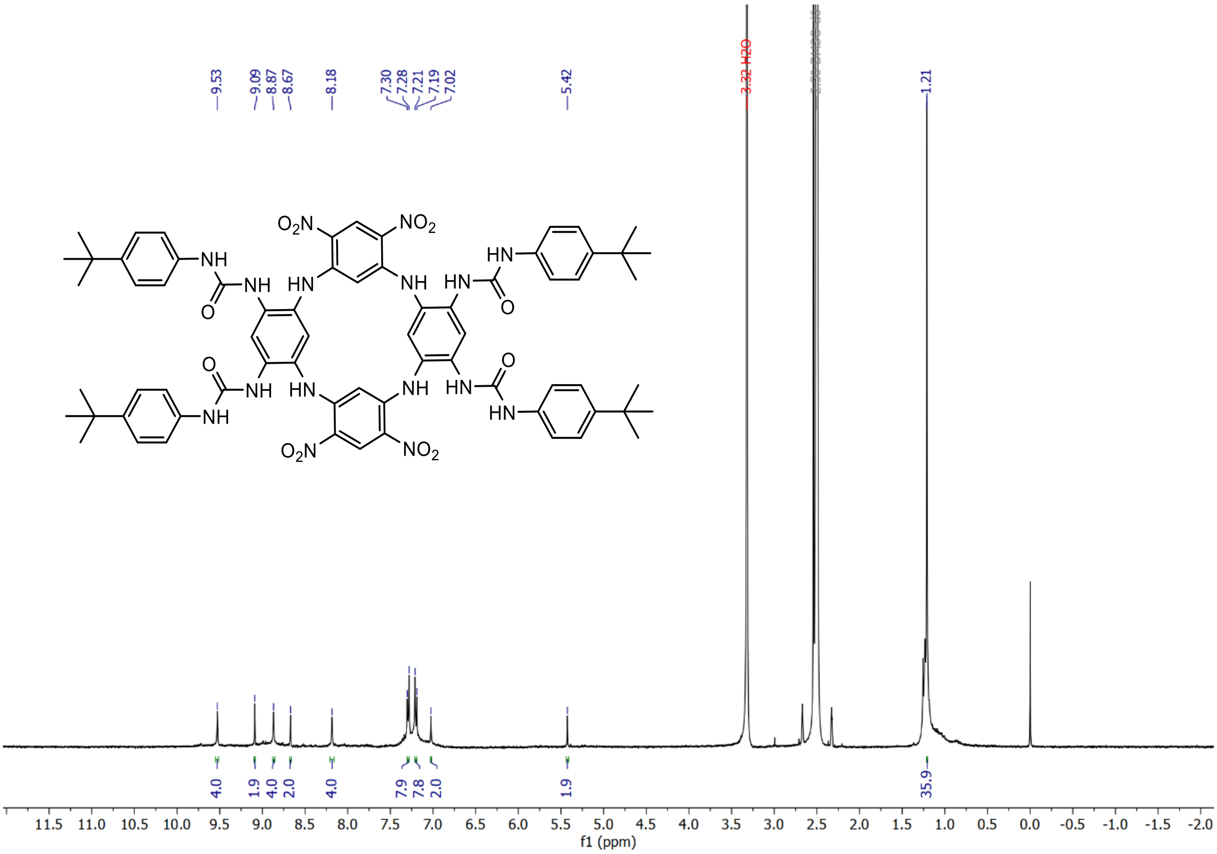

Fig. S56: ^{1}H NMR of compound **6** (DMSO-d_6, 400 MHz).

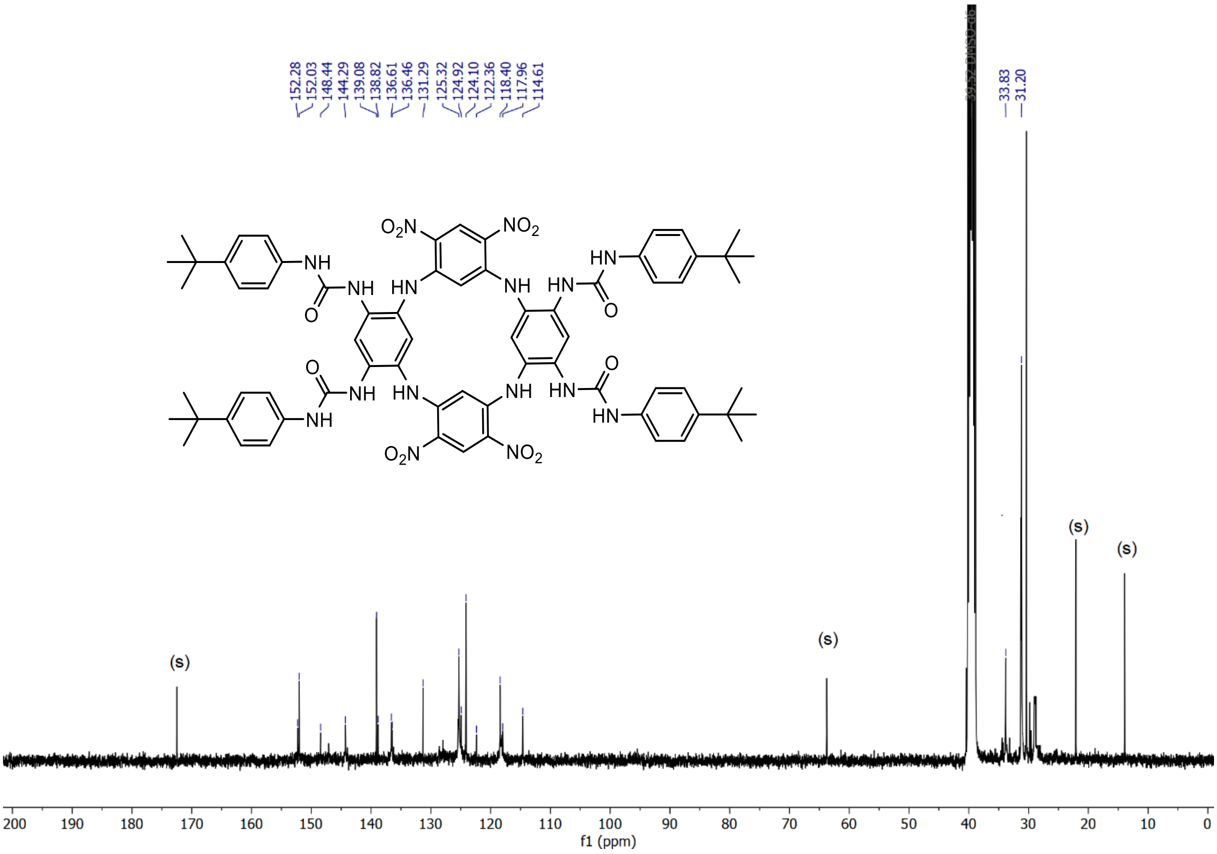

Fig. S57: ^{13}C{^{1}H} NMR of compound **6** (DMSO-d_6, 100 MHz).

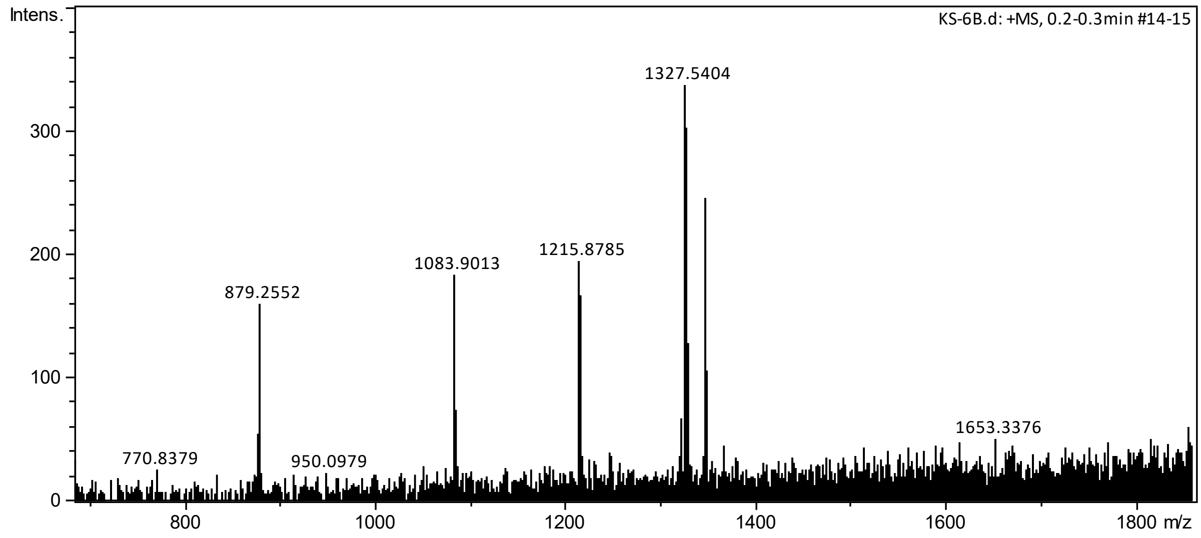

Fig. S58: HRMS (ESI+) of compound **6** calcld. $[C_{68}H_{72}N_{16}O_{12}+Na]^+$ 1327.5408; found 1327.5404 $[M+Na]^+$.

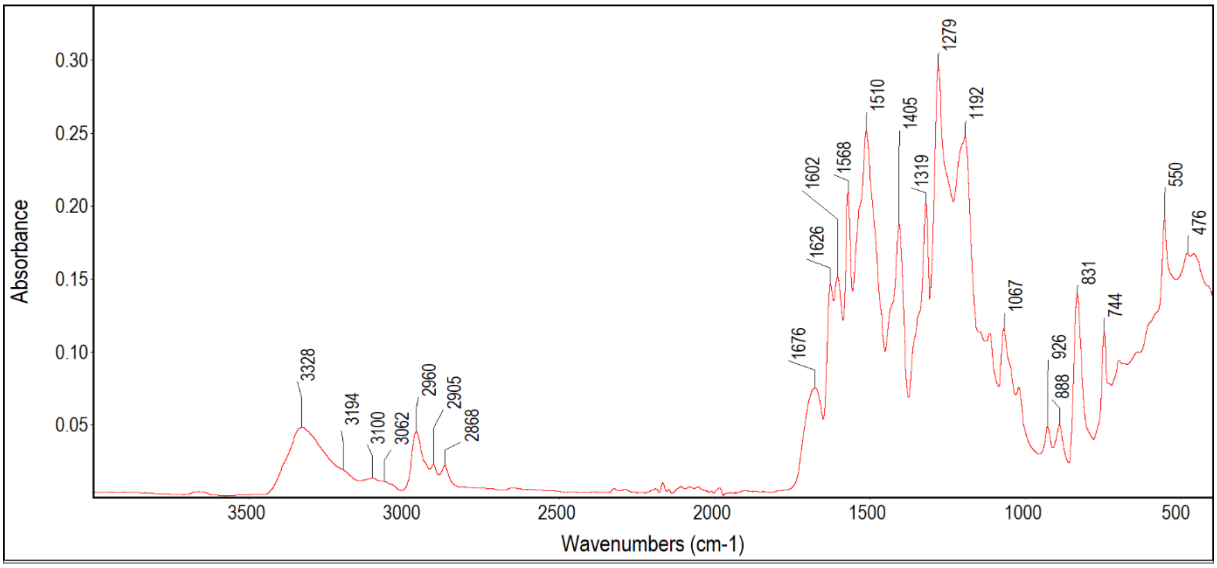

Fig. S59: IR (ATR) spectrum of compound **6**.

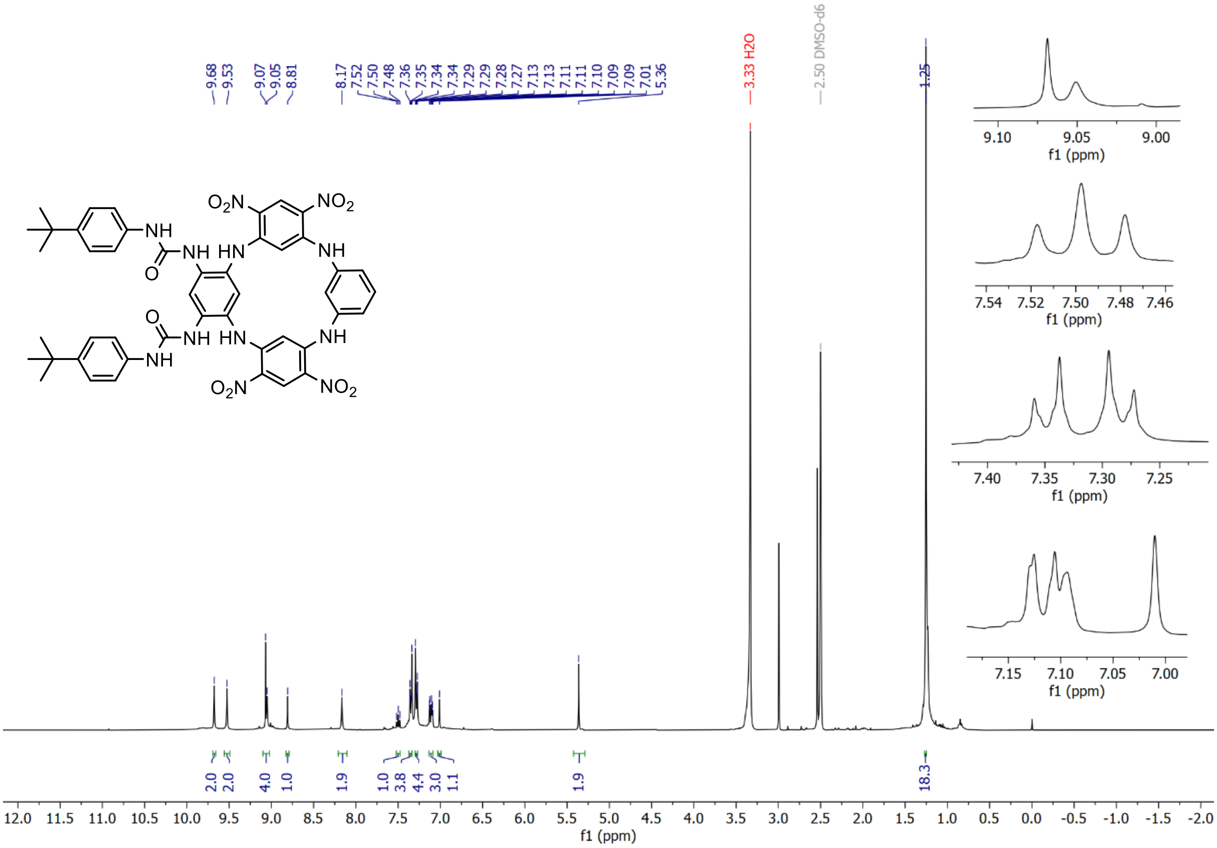

Fig. S60: ^{1}H NMR of compound **7** (DMSO-d_6, 400 MHz).

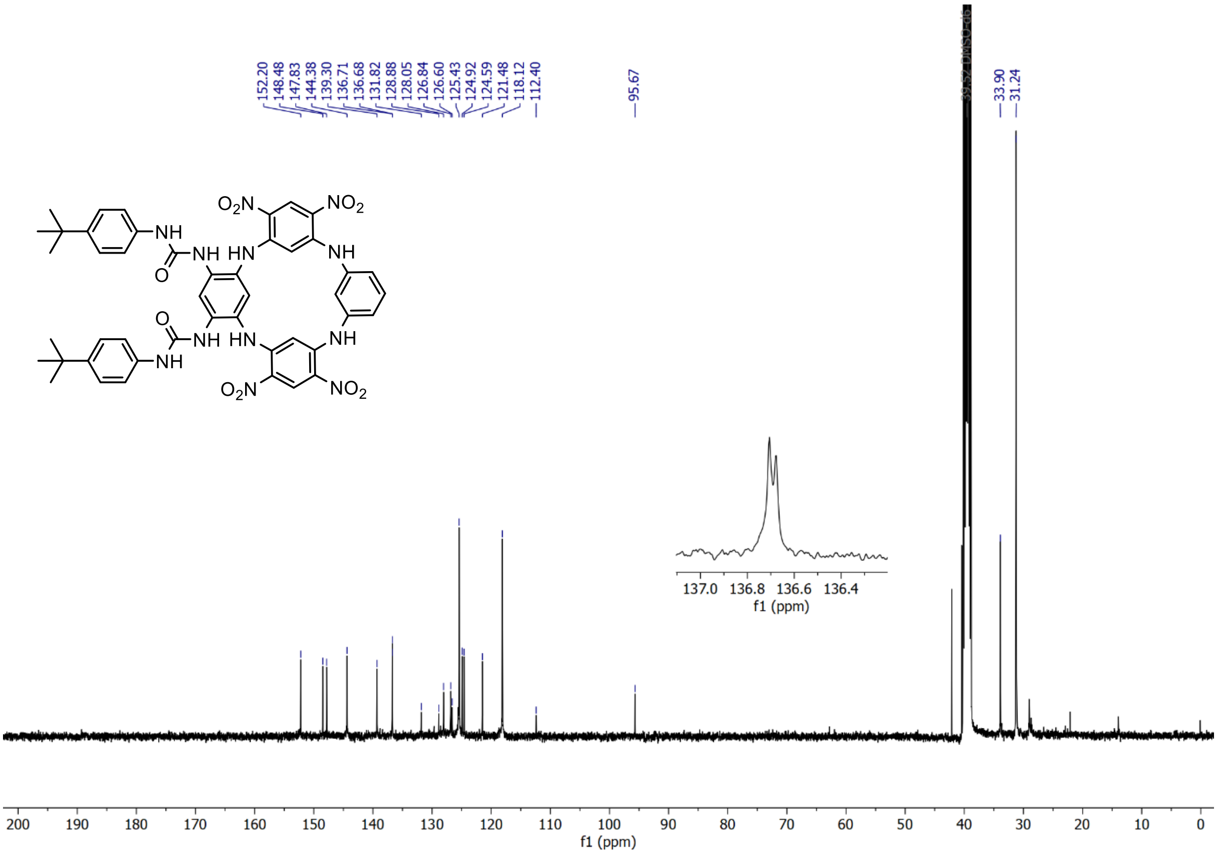

Fig. S61: ^{13}C{^{1}H} NMR of compound **7** (DMSO-d_6, 100 MHz).

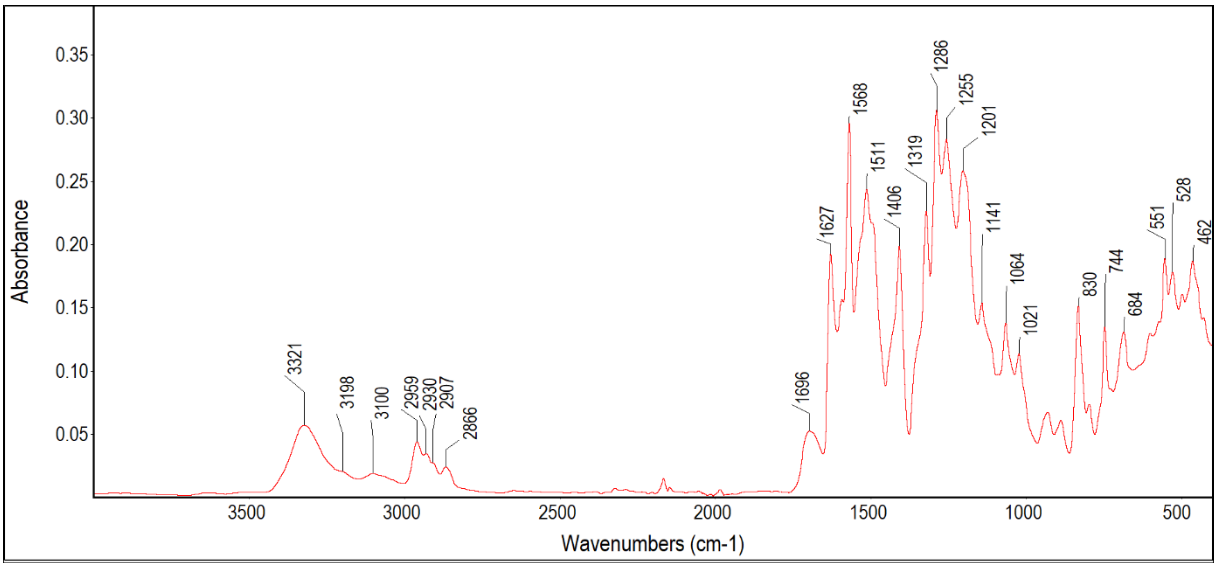

Fig. S62: IR (ATR) spectrum of compound **7**.

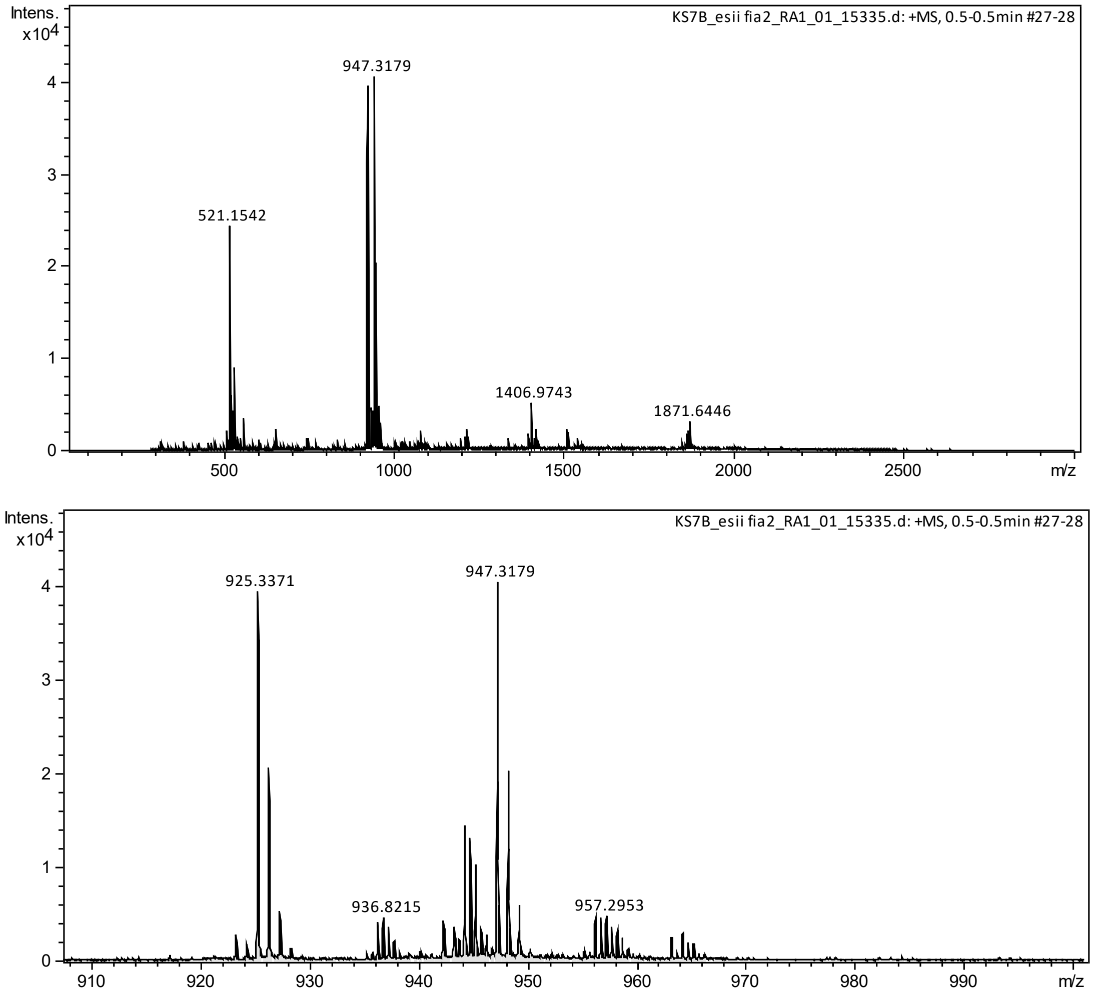

Fig. S63: HRMS (ESI+) of compound **7** calcld. $[C_{46}H_{44}N_{12}O_{10}+H]^+$ 925.3376; found 925.3371 $[M+H]^+$; calcld. $[C_{46}H_{44}N_{12}O_{10}+Na]^+$ 947.3196; found 947.3179 $[M+Na]^+$.
